# Printed magnetoresistive sensors for recyclable magnetoelectronics†

**DOI:** 10.1039/d4ta02765e

**Published:** 2024-08-15

**Authors:** Xiaotao Wang, Lin Guo, Olha Bezsmertna, Yuhan Wu, Denys Makarov, Rui Xu

**Affiliations:** a Helmholtz-Zentrum Dresden-Rossendorf e.V., Institute of Ion Beam Physics and Materials Research Bautzner Landstrasse 400 01328 Dresden Germany r.xu@hzdr.de d.makarov@hzdr.de; b School of Environmental and Chemical Engineering, Shenyang University of Technology Shenyang China

## Abstract

We have developed an innovative recyclable printed magnetoresistive sensor using GMR microflakes and AMR microparticles as functional fillers, with PECH as the elastomer binder. Under saturation magnetic fields of 100 mT and 30 mT, these sensors respectively exhibit magnetoresistance values of 4.7% and 0.45%. The excellent mechanical properties and thermal stability of the PECH elastomer binder endow these sensors with outstanding flexibility and temperature stability. This flexibility, low cost, and scalability make these sensors highly suitable for integration into flexible electronic devices, such as smart security systems and home automation. Moreover, these sensors are fully recyclable and reusable, allowing the materials to be separated, reused, and remanufactured without loss of performance. The low energy consumption of the production process and the recyclability of the materials significantly reduce the environmental impact of these magnetic field sensors.

## Abstract 2

We validate the concept of fully recyclable printed magnetoelectronics, which not only enables low-cost high-throughput fabrication of magnetic sensors, but also importantly reduces the environmental burden related to electronic wastes.

## Graphical abstract

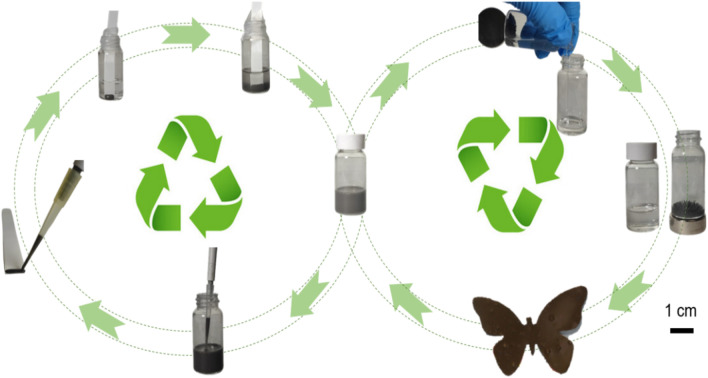

## 1. Introduction

With the development of consumer electronics and Internet of Things (IoT), multifarious electronics have become an indispensable companion of our society, enormously reshaping the way we work, live, are entertained, consume, *etc.* The rapid expansion of the electronics industry has given rise to the skyrocketing volume of electronic waste (e-waste), making it one of the fastest-growing waste streams over the past few decades.^1^ According to the latest survey report of the United Nations in 2020, about 53.6 million metric tons of e-waste were generated worldwide in 2019 (approximately 7 kg per person per year), with less than 20% being sent for recycling.^2,3^ As a result, many constituent materials, along with all associated inputs in energy and investment, are squandered, leading to resource wastage, shortage of raw materials, and pollution issues.^1–7^ E-wastes have become a significant environmental burden, casting a shadow of immense instability over future development.

As a fundamental component of electronics, magnetic field sensors find wide application in the automotive industry, IoT, biomedicine, and consumer electronics,^8–13^ benefiting from their capabilities for measuring positions, angles, orientations and movements, as well as touchless interactions characterized by high reliability and sensitivity.^14–18^ The typical fabrication process of the magnetic field sensors relies on energy-intensive techniques and expensive facilities, due to the demand for intricate structures necessary for precise sensing capabilities (*e.g.*, multiple nanoscaled stacks for a giant magnetoresistance effect).^19–24^ Compared with the conventional lithography based fabrication, the energy consumption of printing techniques is substantially lower.^25^ The bulky configurations of conventional magnetic field sensors further limit their recycling capability due to the critical requirements for complex processes and harsh treatments (*e.g.*, high temperature, high pressure, combustion, strong acid/alkali, *etc.*).^2,3^ In addition, their component materials typically contain hazardous metals of cobalt and nickel and their alloys,^22,26–30^ which pose potential risks to the ecology and public health if not properly recycled. Given that the adverse impacts of magnetic field sensors may arise from the entire lifecycle, holistic considerations should be implemented in different stages of their lifespan.

To address this systemic problem, printed magnetoresistive sensors have been developed to simplify the fabrication process,^26,30–33^ thereby reducing energy consumption and equipment investments. Given their susceptibility to mechanical deformation or damage, particularly in applications such as wearable electronics,^34,35^ self-healing capabilities^36^ have been integrated to prolong the operational lifespan of printed magnetic field sensors. However, the challenge of how to handle discarded sensors after their service life, especially in a convenient manner without the assistance of harmful chemicals^32^ or harsh treatments,^34,35^ remains an open question. Considerable efforts have been dedicated to advancing recyclable electronics.^37–39^ Although a wide spectrum of recyclable sensors,^40,41^ transistors,^42,43^ batteries,^44,45^ optoelectronic devices,^46,47^ and generator devices^48–50^ have been developed, recyclable magnetic field sensors have not been reported yet. To fully mitigate the environmental impacts associated with magnetic field sensors, it is imperative to develop a method that simultaneously reduces energy consumption during initial production and achieves material circularity at the end of their lifecycle.

In this work, we achieved printable magnetic field sensors with full recyclability. To validate the technical feasibility, we printed two types of recyclable sensors with different forms of functional fillers, *i.e.*, giant magnetoresistive (GMR) sensors with [Co/Cu]_50_ multi-stack microflakes and anisotropic magnetoresistive (AMR) sensors with Ni_97_Co_3_ microparticles. The polyepichlorohydrin (PECH) elastomer was selected as the polymer binder. It exhibits significant volume shrinkage during solvent evaporation, alongside a high viscosity capable of driving filler movement. These properties work together to guide the rearrangement of fillers, consequently forming electrical percolation in the printed composites. The formed sensors demonstrate a decent magnetoresistance (MR) response with the saturation MR values of about 4.7% at 100 mT for the printed GMR sensors and 0.45% at 30 mT for the printed AMR sensors. Because of the robust electrical pathways, the printed sensors feature low noise and high operational stability. After 1000 cycles of magnetic interaction, their sensing performances have little variation. The thermal stability of PECH endows our sensors with a reliable MR response even after exposure to thermal treatments up to 80 °C, which meets the requirements of consumer electronics. The repeatable dissolution property of PECH allows our samples to be easily decomposed. The fillers inside can be easily extracted from the dissolved polymers *via* external magnetic fields, and then undergo further processing. By virtue of the energy-effective fabrication and the material circularity that generate a minimal environmental footprint, our sensors exhibit promising potential in IoT applications that require a huge amount of low-cost sensors for real-time information exchange, as evidenced by the implementation of magnetic sensing in smart home systems.

## 2. Experimental section

### 2.1 Printing fabrication of magnetoresistive sensors

The chemicals for the synthesis of the inks, including Ni_97_Co_3_ AMR microparticles (Ni 97%/Co 3%, mean particle size of 50 μm), polyepichlorohydrin (Assay 98%), and acetone (Assay ≥ 99.9%), were sourced from Sigma-Aldrich Co. LLC (Fig. S1a†). The [Co/Cu]_50_ GMR microflakes were fabricated *via* magnetron sputtering deposition. Multilayer GMR stacks composed of [Co (1 nm)/Cu (2.2 nm)]_50_ coupled at the 2nd antiferromagnetic maximum were deposited on AZ 1505 (MicroChemicals GmbH, Germany) coated substrates (Fig. S1b†). The Ni_97_Co_3_ AMR microparticles and [Co/Cu]_50_ GMR microflakes were used as the AMR and GMR magnetoresistive fillers, respectively. Scanning electron microscopy (SEM) and energy dispersive X-ray (EDX) analysis were performed to investigate the morphology and composition of the magnetoresistive fillers by using the Phenom XL Desktop Scanning Electron Microscope (Thermo Fisher Scientific, United States). The elastomer binder solution consists of polyepichlorohydrin (PECH) and acetone with a mass ratio of 1 : 9. The mixture was subjected to stirring at 60 °C for 24 hours on a magnetic stirrer hotplate. PECH is a flexible material with an elastic modulus of 0.5 MPa, tensile strength of 17 MPa, and tear strength of 36 kN m^−1^. It remains elastic at low temperatures (glass transition temperature: −14.46 °C). It can be dissolved in acetone and other organic solvents and has low toxicity.^51–53^ By virtue of these properties, PECH paves the way for applications requiring flexibility, durability, and recyclability. The Ni_97_Co_3_ AMR microparticles with a volume ratio of 50% or [Co/Cu]_50_ GMR microflakes with a concentration of 40 mg mL^−1^ were incorporated into the elastomer binder to produce printable composites. The mixtures were agitated using a digital vortex mixer (VWR) at 2500 rpm for 60 seconds to ensure a homogeneous dispersion of functional fillers. The developed composites were printed onto diverse substrates (*e.g.*, FFC cables, commercially available from TME Germany GmbH), followed by drying at room temperature for 180 minutes. For flexible applications, PET films served as the substrate. The electrodes for measurements were pre-deposited on the substrates using electron beam evaporation and the required patterns for the electrodes were defined by the corresponding shadow masks.

### 2.2 Characterization of magnetoresistance and noise for the printed magnetoresistive sensors

The electrical resistances of the printed magnetoresistive sensors were measured on the base of the Kelvin four-terminal sensing technique, aided with a tensormeter (HZDR Innovation, Germany). To characterize the noise properties, the electrical resistances of the printed sensors were measured for 30 seconds, with the sampling rate set at 50 Hz. After obtaining the time-based resistance changes, a Fast Fourier Transform (FFT) was applied to calculate the noise spectral density of the signal. All measurements were conducted at room temperature. The MR ratio of the sensor is calculated using the formula MR(H) = [(*R*(H) − *R*(H_sat_))/*R*(H_sat_)] × 100%, where *R*(H) refers to the resistance value of the sample under the current magnetic field, and *R*(H_sat_) refers to the resistance value of the sample in the magnetically saturated state. The sensitivity of the sensor is calculated using the formula *S*(H) = [d*R*(H)/dH]/*R*(H), which is defined as the first derivative of the sample's resistance with respect to the external magnetic field divided by the resistance value *R*(H).

### 2.3 Evaluation of the operational stability for the printed magnetoresistive sensors

For the long-term operation test, the electrical resistance values of the samples were continuously monitored using the tensormeter. The variations in magnetoresistance across 1000 cycles of magnetic fields were recorded. Regarding the temperature stability, two types of magnetoresistive sensors were subjected to thermal treatments using a hot plate. The sensors were securely mounted on the hot plate, and the temperature settings were adjusted accordingly. Once the hot plate achieved the specified temperatures, each sample was maintained at these temperatures for 10 minutes. Subsequently, the samples were removed and cooled at room temperature for 10 minutes. The magnetoresistance of each sensor was then measured to assess the effects of thermal exposure. The thermal effects at three temperatures of 40 °C, 60 °C, and 80 °C were systematically evaluated. To assess the mechanical stability, the printed sensors were affixed onto lab-made gadgets with a curved surface of various diameters (*i.e.*, 30 mm, 20 mm, and 15 mm). The measured magnetoresistance in the bent states was compared with that for the planar sensor.

### 2.4 Application of the printed magnetoresistive sensors in smart home systems

The flexible sensors printed on the PET foil were affixed to a door handle, with a small magnet positioned nearby on the door. When the handle is rotated, the distance between the sensor and the magnet varies, causing changes in magnetoresistance in response to the fluctuating magnetic fields surrounding the sensors. Real-time signal acquisition was performed using a data acquisition system (DAQ, National Instruments, United States), with the output voltage signals monitored through LabVIEW software. When the door handle ceased to rotate and the output signal stabilized for a duration of three seconds, this stability was interpreted as confirmation of a signal input. The magnitude of the signal was compared against a pre-calibrated value to determine the numerical value entered.

## 3. Results and discussion

### 3.1 Fabrication of recyclable printed magnetoresistive sensors

**Fig. 1:**
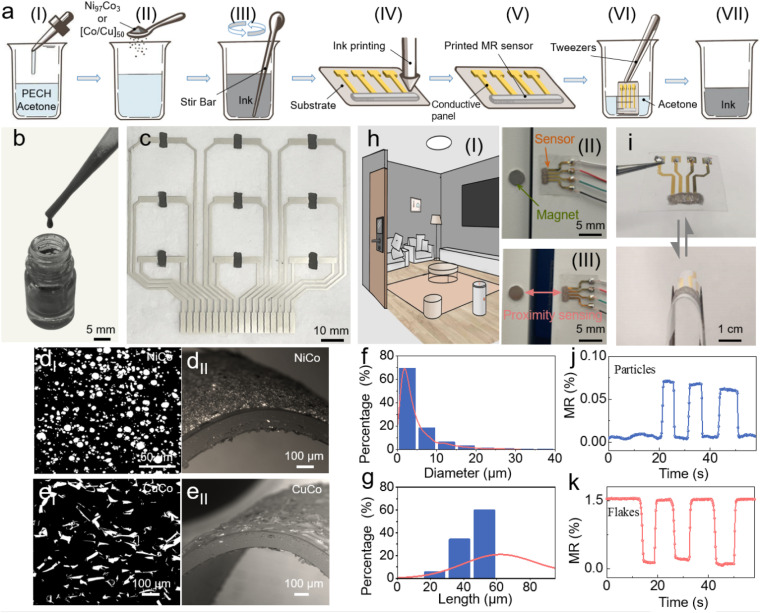
Recyclable and printable magnetoresistive sensors. (a) Schematic illustration of the fabrication and recycling steps for the printable and recyclable magnetoresistive sensors. (a(I)) Form binder solutions by dissolving PECH into acetone; (a(II)) add magnetoresistive fillers into the binder solutions to form printable inks; (a(III)) stir the ink to ensure uniform distribution of functional fillers; (a(IV)) print the ink; (a(V)) cure the ink to obtain a sensor; (a(VI)) place the discarded sensor into acetone; (a(VII)) obtain the new ink. Photographs of (b) the prepared ink and (c) the printed magnetoresistive sensors. SEM images of MR sensors in flat (d(I) and e(I)) and bent (d(II) and e(II)) states, based on the material of (d) Ni_97_Co_3_ AMR microparticles and (e) [Co/Cu]_50_ multilayer GMR microflakes. Size distribution of (f) AMR microparticles and (g) GMR microflakes. (h) Application of the printed magnetoresistive sensor in smart home systems. (h(I)) Schematic illustration; (h(II) and h(III)) sensor and magnet attached onto furniture surfaces for proximity sensing. (i) Photograph of a printed sensor with mechanical flexibility. Magnetoresistance response of the printed sensors with (j) AMR microparticles and (k) GMR microflakes as fillers.

The printing fabrication of the magnetoresistive sensors is schematically illustrated in Fig. 1a(I–V), mainly comprising two steps, *i.e.*, ink preparation and printing. After reaching the end of their lifespan, the printed sensor can be decomposed (Fig. 1a(VI-VII)), allowing for the retrieval of both the magnetic fillers and dissolved polymeric binders for future processing. To prepare the printable ink, the polyepichlorohydrin (PECH) elastomer, serving as the binder, is dissolved into acetone. After complete dissolution, the magnetoresistive fillers are added into the PECH solution. To enhance the printing success rate and performance consistency of the printed sensors, meticulous mixing of the inks before printing is essential, ensuring uniform distribution of the fillers throughout the entire volume (Fig. 1b). Afterwards, the ink is printed onto the substrate with predefined electrodes (Fig. 1c). Following solvent evaporation and consequently a volume shrinkage of 46.7% for the printed composite (Fig. S2†), the fillers form electrical pathways within the composite, imparting the printed traces with the ability to detect magnetic fields. The printed sensors tightly adhere on diverse substrates and the adhesion reliability depends on the substrate material, surface roughness, and magnetic filler concentration (Table S1†). In contrast to conventional magnetoresistive sensors, which rely on energy-intensive processes and expensive high-vacuum equipment for fabrication, our sensors can be easily processed at room temperature under natural conditions.^21–23,28^ This significantly reduces the environmental footprint associated with the fabrication process. Here, Ni_97_Co_3_ AMR microparticles (Fig. 1d) or [Co/Cu]_50_ multilayer GMR microflakes (Fig. 1e) are selected to serve as functional fillers to show the general applicability of our technique to a wide range of fillers regardless of their compositions, geometries and structural configurations. The GMR microflakes can be sourced from residual waste generated during traditional GMR sensor production, such as GMR microflakes peeled off after photolithography in order to form specific patterns, or remnants left on the inner walls of the sputtering equipment during deposition processes. This approach is viable for enhancing the utilization efficiency of raw materials and effectively transforming waste into treasure, thus alleviating environmental burden. However, the supply of GMR microflake wastes is limited and in small quantities. In order to address the high demand for massive sensor deployment in the IoT era, ensuring a stable, reliable, and cost-effective source of magnetoresistive fillers is crucial. Alternative fillers in the form of AMR microparticles are readily available and require minimal processing, rendering them low-cost, low-energy, and low-carbon-footprint raw materials compared to other fillers with nanoscale characteristics.^54,55^Fig. 1j and k show the MR curves of the printed GMR sensor and the AMR sensor during magnetic interactions. The successful achievement of a stable binding state and electrical percolation in both sensors confirms the extensive compatibility of our technique regardless of the filler dimensions in terms of size and shape (Fig. 1d and e). Adequate material supply, coupled with relatively low environmental impact and cost-effective large-scale manufacturing processes, paves the way for the widespread adoption of magnetic sensors to provide touchless interaction in the IoT applications. For instance, the printed sensors can be seamlessly integrated into household furniture (*e.g.*, doors, drawers) to enable smart homes. As magnetic sources approach the sensors (Fig. 1h), their MR exhibit obvious variation. Due to the unique spin-dependent transport properties inherent in multilayer GMR microflakes and AMR microparticles, their MR responses exhibit different trends (*i.e.*, negative and positive changes respectively). The GMR is related to the magnetization direction of the ferromagnetic thin film layers separated by a non-magnetic spacer in the multilayer stack. When the magnetization directions of the neighbouring layers are antiparallel, the resistance value is significantly greater than when the magnetization directions are parallel. The applicability of both the typical morphologies (flakes and particles) also exhibits the technique's general utility for the recyclable printed MR sensors based on the PECH elastomer binder. Benefiting from the mechanical flexibility of the binder material PECH, our printed sensors exhibit high resilience (Fig. 1i). This characteristic paves the way for printed magnetoresistive sensors in emerging applications such as wearable electronics and sticker electronics.

### 3.2 Performance characterization and operational stability test for the printed sensor

**Fig. 2:**
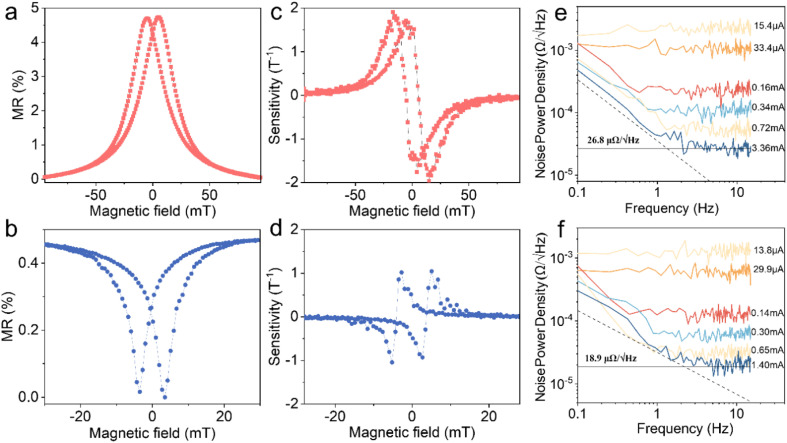
Magnetoelectric performance of printed magnetoresistive sensors. (a) MR, (c) sensitivity and (e) noise of the printed sensor with [Co/Cu]_50_ GMR microflakes as functional fillers. (b) MR, (d) sensitivity and (f) noise of the printed sensor with Ni_97_Co_3_ AMR microparticles as functional fillers. All measurements are performed at room temperature.

To fulfill the touchless sensing functionality mentioned above, the magnetoresistance performance and noise characteristics of the printed sensor play critical roles. The magnetoresistance characterization is carried out with the standard four-point method by measuring the dependence of the electrical resistance variation of the printed active trace on applied magnetic fields. The printed GMR sensor with [Co/Cu]_50_ GMR microflakes as fillers shows a saturation MR of about 4.7% at 100 mT (Fig. 2a). Although the printed AMR sensor with Ni_97_Co_3_ AMR microparticles as fillers has lower MR ratios, *e.g.* with a maximum MR of about 0.45%, its saturation field is reduced to about 30 mT (Fig. 2b). The sensitivity further highlights the distinct operational magnetic field ranges of the two sensors. In the case of the GMR sensor, a maximum sensitivity of 1.8 T^−1^ is observed at about 15 mT and 3 mT (Fig. 2c), whereas for the AMR sensor, a maximum sensitivity of 0.9 T^−1^ is observed at about 5 mT and 2 mT (Fig. 2d). By providing a range of operational magnetic fields, our magnetic sensors meet the diverse needs of various applications. These include biomedical devices that operate within narrow magnetic ranges and consumer electronics that necessitate functionality within stronger magnetic fields.^34,56^ To further enhance the measurement accuracy of the sensors, it is essential to reduce the hysteresis in the MR curves. Luckily, the flexible fabrication process of our printed sensors is widely compatible with a wide range of fillers, independent of their compositions, geometries and configurations, as well as with various compensation techniques. Therefore, it is believed that by selecting appropriate materials (*e.g.*, soft magnetic materials),^57,58^ altering the configuration of the fillers,^59,60^ inducing a bias magnetic field or adding antiferromagnetic layer to pin the initial magnetic moments in a predefined direction with an exchange bias effect,^61–63^ or incorporating compensation techniques,^64–66^ the printed sensor will be characterized with reduced hysteresis and enhanced accuracy. Signal noise of the printed sensors can undermine their ability to provide accurate and reliable measurements. It directly determines the sensing performance especially for the printed sensors, in which the conductive pathways are made of fillers, rather than a continuous film. Both types of our sensors exhibit minimal electrical noise, indicating close physical contact between fillers. For example, the noise power density is 26.8 μΩ √Hz^−1^ at a current of 3.36 mA and 1.32 Hz for the sensor with GMR microflake fillers (Fig. 2e), and 18.9 μΩ √Hz^−1^ at a current of 1.40 mA and 2.18 Hz for the sensor with AMR microparticle fillers (Fig. 2f). These low electrical noises allow the sensors to detect magnetic fields with a maximum resolution of about 0.2 μT and 1.1 μT, respectively, enabling precise measurement in various applications.

**Fig. 3:**
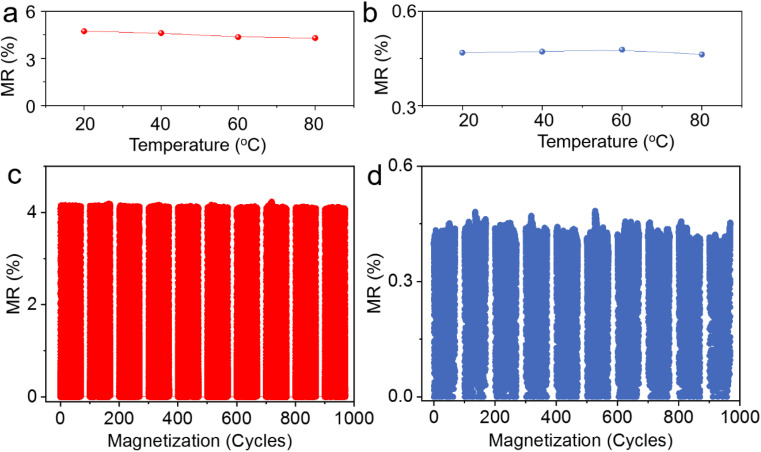
Operational stability of printed magnetoresistive sensors. The saturation MR values of (a) the printed GMR microflake-based sensors and (b) the AMR microparticle-based sensors after exposure to high temperatures. MR variation of the printed sensors with (c) GMR microflakes and (d) AMR microparticles as fillers during cycled magnetic interaction.

In order to evaluate the practical reliability of the printed sensors, we conduct temperature stability tests on their MR performance across a range from room temperature up to 80 °C. This temperature range is selected to align with the operational requirements of typical consumer electronics. Our findings reveal that both the printed GMR microflake and AMR microparticle sensors maintain their functionality effectively even after exposure to thermal treatment at 80 °C (Fig. 3a and b). This robustness can be attributed to the low glass transition temperature (−14 °C) of the elastomer binder PECH, which ensures minimal phase change and volume expansion within this temperature regime, thus preserving electrical conductivity. However, we observed a slight degradation in MR performance (less than 9.2%) in the printed GMR microflake samples, which can likely be attributed to the low volume ratio of GMR microflake fillers. Even a minor expansion of the binder can have a notable impact on electric percolation in such cases. Both types of printed sensors demonstrate remarkable stability in repeated magnetic interactions (Fig. 3c and d). No significant performance deterioration was noted after subjecting them to 1000 magnetic cycles, underscoring their robustness and suitability for prolonged use in real-world applications.

### 3.3 Recycling and reprocessing strategies

**Fig. 4:**
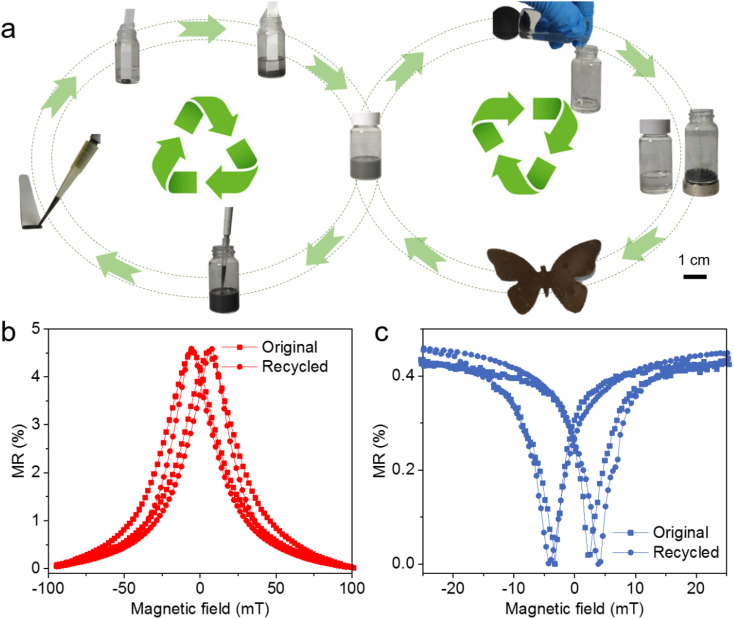
Recyclable properties of printed magnetoresistive sensors. (a) The left loop illustrates the recycling and reproduction process of printed magnetoresistive sensors: discarded sensors are immersed in solvent to decompose magnetoresistive fillers and polymer binders, after which the recycled ink is used to fabricate new sensors. The right loop illustrates the separation of component materials and the fabrication of a magnetic soft robot using the separated polymers and magnetic fillers. (b and c) MR response of the printed sensors with the recycled materials. The sensors exploit (b) GMR microflakes and (c) AMR microparticles as fillers.

Once the printed magnetoresistive sensors have completed their function or are no longer in use, valuable materials such as polymers and magnetic fillers can undergo a simple recycling process to be reprocessed, as illustrated in the left cycle of Fig. 4a, thereby preventing the generation of new electronic waste. For instance, a certain quantity of discarded sensors is placed into acetone according to the concentration requirements for the new ink. The PECH binder demonstrates excellent solubility in acetone, enabling the sensors to fully decompose within a few minutes, *e.g.*, less than 10 minutes (Fig. S3†). The resulting ink can then be utilized to print sensors of various configurations (*e.g.*, size, shape, concentration) to meet diverse practical needs. It's noteworthy that the recycled sensors, whether composed of [Co/Cu]_50_ GMR microflakes (Fig. 3b) or Ni_97_Co_3_ AMR microparticles (Fig. 3c), exhibit no performance degradation, even after undergoing multiple recycling and re-fabrication processes (Fig. S4†), validating the reliability of our recycling method. This recycling procedure is straightforward and swift, achieving 100% material reuse. If the materials constituting these sensors are not intended for manufacturing sensors again, the recycling strategy can be altered accordingly. Due to their ferromagnetic properties, functional fillers can be effortlessly extracted and separated from the ink. The separated PECH elastomer binder boasts excellent mechanical properties, allowing it to be transformed into magnetic soft robots with self-sensing capabilities by adjusting the filler composition and concentration,^67^ as depicted in the left cycle of Fig. 4a. Furthermore, the recycled materials can also find utility in other fields such as 3D printing or additive manufacturing. As this technology reduces the demand for raw materials, it holds promise for lowering manufacturing costs and promoting sustainable development.

### 3.4 Sticker electronics application for smart homes and smart security

**Fig. 5:**
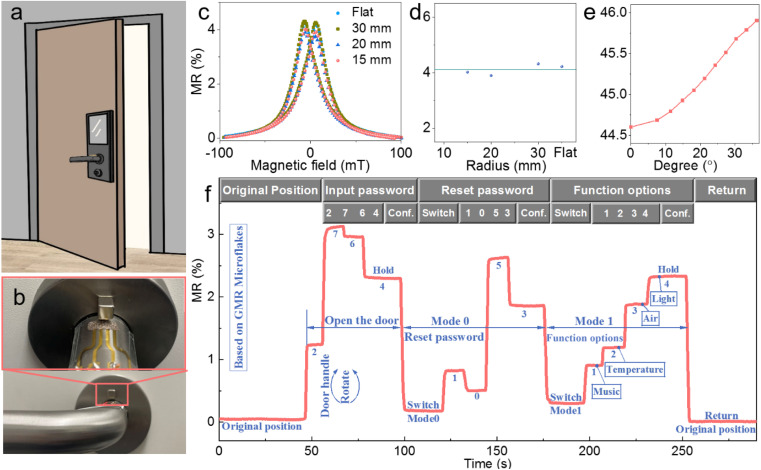
Sticker electronics application for smart homes and security. (a) Schematic illustration and (b) photograph of a printed magnetoresistive sensor for the application of sticker electronics in smart homes and security. In (b), the printed magnetoresistive sensor adheres conformably to a door handle, and can sense the magnetic fields generated by a permanent magnet placed around the sensor. (c) Magnetoresistance curves and (d) maximum values of printed sensors bent to different curvatures. (e) Electrical resistance changes in response to the rotation angle of the door handle. As the handle rotates, the spatial position of the sensor relative to the magnet changes, along with the magnetic field experienced by the sensor. (f) Conceptual realization of smart homes with a sensor-equipped door handle.

By virtue of the aforementioned advantages such as low cost, flexibility, recyclability, temperature stability, and reliability, printed magnetoresistive sensors hold significant promise in the realm of sticker electronics. Here, we showcase the application of printed magnetoresistive stickers in smart security and home automation systems. In our everyday lives, numerous scenarios demand the attachment of electronic devices to curved surfaces (Fig. 5a). Typically, for curved surface applications, electronic devices must undergo specific design and fabrication tailored to each product, given the variations in the size and curvature radius of such surfaces. Customized sensors entail high costs and inconvenience for users. The outstanding flexibility of printed magnetoresistive sensors allows for their pre-design and fabrication as flat, sticker-like electronic devices. In practical applications, they can conform precisely to the required shape, maintaining adherence to object surfaces without altering the magnetic response performance (Fig. 5c). For instance, we printed a magnetoresistive sensor on a flexible PET substrate serving as a sticker electronic device. We then affixed the sticker to the curved surface of a door handle and secured a permanent magnet on the door (Fig. 5b). Rotating the door handle alters the distance between the magnetoresistive sensor and the permanent magnet, enabling the sensor to detect the rotation state and position of the door handle (Fig. 5d). We encoded the rotation of the door handle using the MR value of the magnetoresistive sensor, dividing the movement of the door handle into different regions and assigning functions such as “0 to 9”, “Reset Password”, and “Function Options” (Fig. 5e). If movement within a region persists for more than 3 seconds, it is defined as “confirmation”. Following the preset protocol, the magnetoresistive door handle can be utilized in smart security and home automation systems. As shown in Fig. 5f, we demonstrated applications such as inputting and resetting passwords for smart security, as well as controlling music playback, temperature adjustment, air purification, and lighting through door handle rotation for home automation. Considering that opening a door is typically the initial action when entering a room, integrating multifunctional features into the door holds significant potential for enhancing user convenience.

## Conclusions

We have introduced a strategy for recyclable printed magnetoresistive sensors. To validate their practical feasibility, we fabricated two types of sensors using GMR microflakes and AMR microparticles as functional fillers, respectively. They exhibit MR values of 4.7% and 0.45% under saturation magnetic fields of 100 mT and 30 mT, respectively. Thanks to the outstanding mechanical properties and temperature stability of the PECH elastomer binder, our printed magnetoresistive sensors exhibit remarkable flexibility, enabling operation over a wide temperature range. Due to the advantages of mechanical flexibility, low cost, and mass production capability, these sensors hold tremendous potential in the field of sticker electronics. We exemplify their application in a smart door system, where printed magnetoresistive sensors are utilized for smart security and home automation. While the magnetoresistance performance of our printed sensors may not match that of their thin-film counterparts, their low raw material and manufacturing costs render them valuable for applications where high performance and sensitivity are not paramount but cost-effectiveness is crucial. Leveraging the magnetic properties of the functional fillers and the repeatable solubility of the PECH binder in acetone, our printed magnetoresistive sensors demonstrate excellent recyclable properties: constituent materials can be separated, reused, and refabricated without degradation in magnetoresistance performance. The low energy consumption during fabrication and their recyclable nature hold significant potential for reducing the environmental impact associated with magnetic field sensors. Ultimately, this recycling strategy can be extended to other magnetic functional elements, such as printed magnets and printed magnetic soft robots.

## Data availability

The data supporting this article have been included as part of the ESI.†

## Author contributions

X. W. and L. G. contributed equally to this work. R. X. conceived the concept. X. W. designed and fabricated the sensors and conducted the experiments and data collection. L. G. designed and performed the recycling experiments. X. W., L. G., O. B., Y. W., R. X., and D. M. analyzed the data. L. G. and R. X. wrote the manuscript with comments from all authors. All co-authors edited the manuscript. R. X. and D. M. supervised the project.

## Conflicts of interest

There are no conflicts to declare.

## Supplementary Material

TA-012-D4TA02765E-s001
