# Supplementary material for: Printed magnetoresistive sensors for recyclable magnetoelectronics
Source: J Mater Chem A Mater. 2024 Aug 15;12(37):24906–15. doi: 10.1039/d4ta02765e (PMC11367592; doi:10.1039/d4ta02765e)
Supplement: TA-012-D4TA02765E-s001 [file TA-012-D4TA02765E-s001.pdf]

Supplementary Information (SI) for Journal of Materials Chemistry A.
This journal is © The Royal Society of Chemistry 2024

Supplementary information:

Printed magnetoresistive sensors for recyclable magnetoelectronics

Xiaotao Wang,‡[a] Lin Guo,‡[a] Olha Bezsmertna,[a] Yuhan Wu,[b] Denys Makarov*[a] and Rui Xu*[a]

[a]Helmholtz-Zentrum Dresden-Rossendorf e.V., Institute of Ion Beam Physics and Materials Research, Bautzner Landstrasse 400, 01328 Dresden, Germany. Email: r.xu@hzdr.de; d.makarov@hzdr.de

[b]School of Environmental and Chemical Engineering, Shenyang University of Technology, Shenyang, China

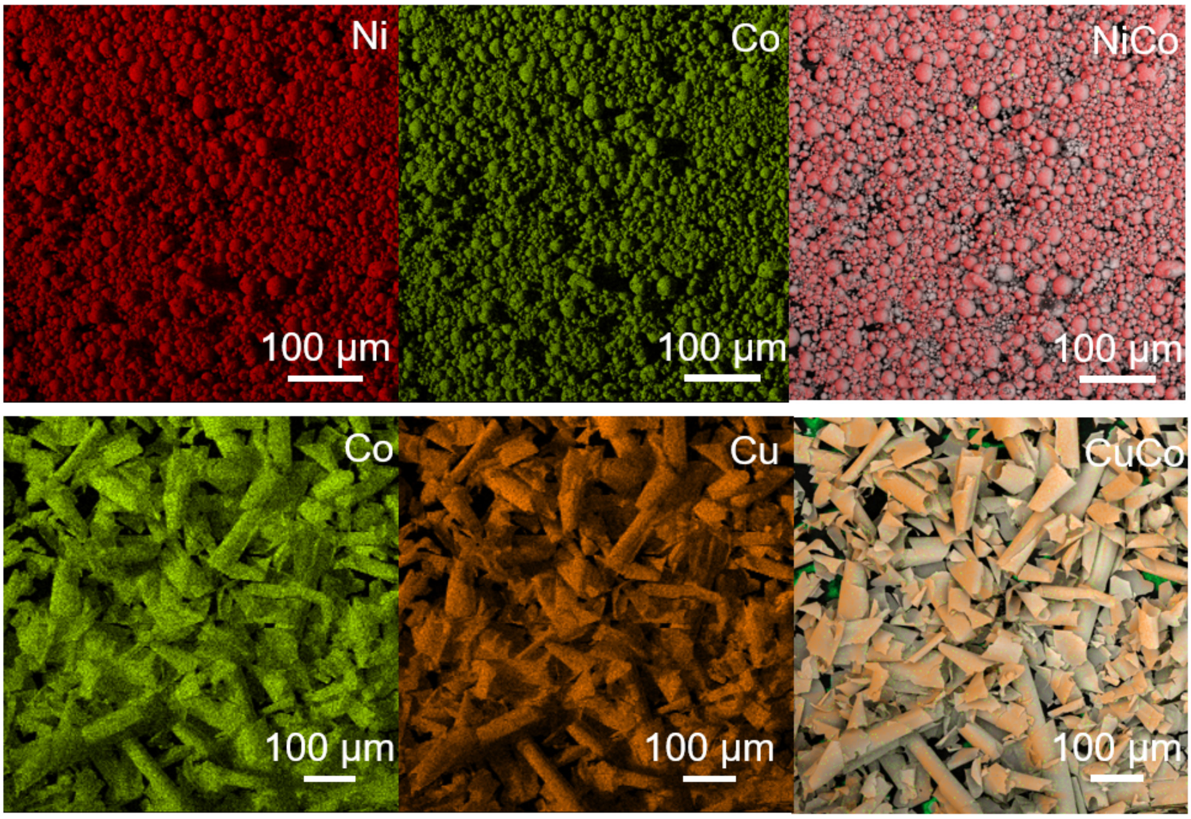

Fig. S1. EDX element mapping of (a) $Ni_{97}Co_3$ microparticles, (b) $[Co/Cu]_{50}$ multilayer microflakes.

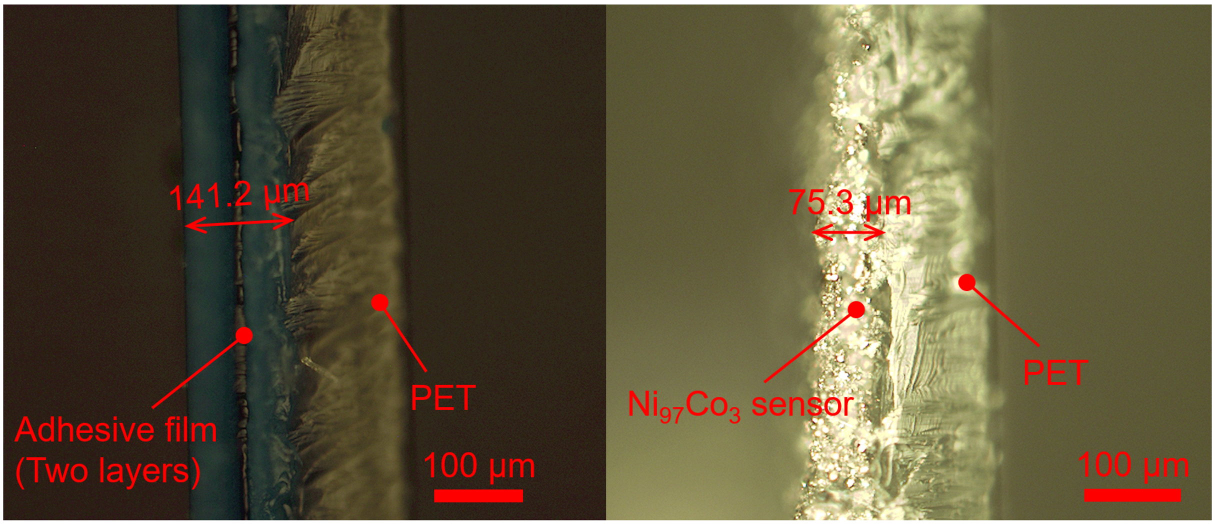

Fig. S2. Optical microscope images of the printed sensor: (left) before and (right) after curing.

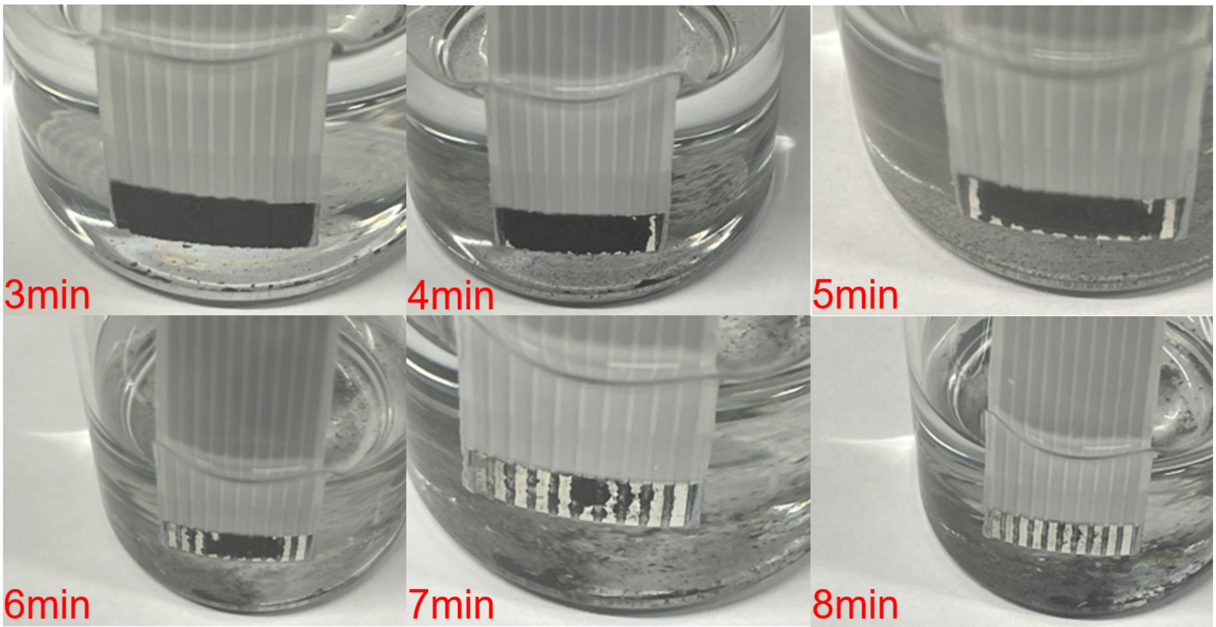

Fig. S3. The decomposition process of the printed sensor in acetone.

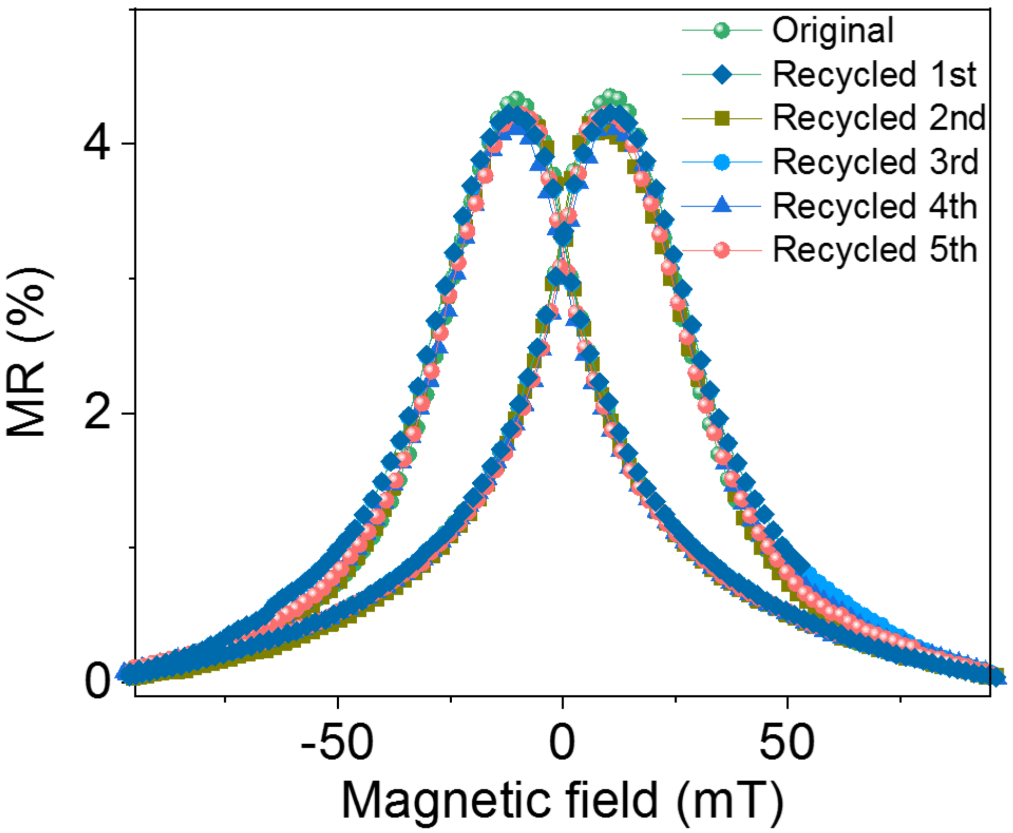

Fig. S4. Variation of the MR performance during five recycling experiments.

Table S1. Classification of the adhesion force. According to the ASTM D3359 standard, we evaluated the adhesion force of magnetic field sensors based on $[Co/Cu]_{50}$ microflakes and $Ni_{97}Co_3$ microparticles using the tape test method. We tested the adhesion force of the printed sensors on various surfaces. It was found that adhesion force is influenced by factors such as substrate material, surface roughness, and magnetic filler concentration.

	Glass Sheet	PET Film	Aluminum Plate
Ink($[Co/Cu]_{50}$ microflakes)	0B	3B	2B
Ink($Ni_{97}Co_3$ microparticles)	0B	3B	2B

Data/Fig. 1f:

Diameter (μm)	Percentage (%)
0-5	69.45
5-10	18.21
10-15	6.55
15-20	3.07
20-25	1.25
25-30	0.72
30-35	0.43
35-40	0.32

Data/Fig. 1g:

Length (μm)	Percentage (%)
15-30	5.71
30-45	34.29
45-60	60

Data/Fig. 1j:

Time (s)	MR (%)
0	0.00455
0.20047	0.00436
0.39996	0.00503
0.6014	0.0054
0.80187	0.00589
1.00235	0.00593
1.20379	0.00612
1.40425	0.00642
1.60572	0.00631
1.80618	0.00608
2.00671	0.00642
2.20614	0.00533
2.40658	0.00574
2.60704	0.00576
2.80751	0.00572
3.00697	0.00584
3.20746	0.00607
3.40696	0.00571
3.60837	0.00553
3.80886	0.00542
4.00833	0.00551
4.20976	0.00561
4.41023	0.00506
4.61069	0.00501
4.81016	0.00448
5.01065	0.00451
5.21015	0.00479
5.41155	0.00454
5.61102	0.00388
5.8126	0.00432
6.013	0.00358
6.21345	0.00332
6.4129	0.00359
6.61334	0.00363
6.81483	0.00402
7.0143	0.00385
7.21573	0.00385
7.41526	0.00425
7.61566	0.00436
7.81513	0.00419
8.0166	0.00477
8.21706	0.00452
8.41755	0.00495
8.61702	0.00481
8.81746	0.00604
9.01792	0.00697
9.21944	0.00631
9.4209	0.00664
9.62133	0.00747
9.82178	0.00718
10.02224	0.0081
10.2227	0.00752
10.42417	0.00859
10.62565	0.00828
10.82516	0.00834
11.02556	0.00886
11.22503	0.00848
11.42552	0.0089
11.62597	0.0087
11.82744	0.00908
12.02888	0.00948
12.22935	0.00928
12.43081	0.00901
12.63027	0.00965
12.83077	0.00926
13.03023	0.00972
13.23167	0.00933
13.43113	0.00961
13.63261	0.00949
13.83312	0.00899
14.03355	0.00938
14.23405	0.00838
14.43546	0.00791
14.63596	0.00805
14.83753	0.00799
15.03887	0.00801
15.23836	0.00722
15.43884	0.00714
15.63925	0.00737
15.83971	0.00704
16.03918	0.00726
16.24063	0.00764
16.44115	0.00741
16.64158	0.00774
16.84203	0.00785
17.04349	0.00743
17.24401	0.00754
17.44445	0.00725
17.64494	0.00687
17.84636	0.00661
18.04682	0.00607
18.2463	0.00602
18.44777	0.00564
18.6482	0.00545
18.84867	0.00532
19.04813	0.00444
19.24866	0.00387
19.44807	0.00424
19.64953	0.00447
19.85	0.00481
20.05147	0.00509
20.25096	0.00551
20.45141	0.00523
20.65186	0.00693
20.85135	0.01023
21.05286	0.02397
21.25327	0.04267
21.45471	0.06427
21.6552	0.07043

21.85665	0.07037
22.0571	0.07031
22.25756	0.07101
22.45803	0.07104
22.65849	0.07048
22.85896	0.07107
23.05942	0.07042
23.26088	0.07121
23.46135	0.07121
23.66188	0.07125
23.86233	0.07088
24.06375	0.07095
24.26421	0.07009
24.4657	0.07009
24.66514	0.06983
24.8656	0.06965
25.06606	0.07007
25.26653	0.0699
25.466	0.06973
25.66646	0.06009
25.86693	0.01815
26.06642	0.00734
26.26785	0.00587
26.46746	0.00556
26.66779	0.00552
26.86728	0.00585
27.06875	0.00571
27.26818	0.00545
27.46865	0.00573
27.66812	0.00528
27.86858	0.00542
28.06904	0.00488
28.27056	0.00505
28.46997	0.00475
28.67144	0.00486
28.87292	0.00476
29.07336	0.00469
29.27485	0.00464
29.47529	0.0037
29.67579	0.00414
29.87622	0.004
30.0767	0.00416

30.27715	0.00436
30.47761	0.00423
30.67708	0.00374
30.87854	0.00416
31.07801	0.00508
31.27849	0.01298
31.47895	0.02988
31.67841	0.0458
31.87987	0.06308
32.07936	0.06705
32.27986	0.06691
32.47929	0.06625
32.67976	0.06688
32.8792	0.06674
33.08067	0.06655
33.28113	0.06681
33.48259	0.06729
33.68206	0.06691
33.88252	0.06769
34.08299	0.06755
34.28251	0.06743
34.48294	0.06787
34.68338	0.06776
34.88387	0.06811
35.08331	0.06824
35.28478	0.06807
35.48524	0.06817
35.68573	0.0683
35.88617	0.06772
36.08663	0.06766
36.28712	0.06776
36.48856	0.06767
36.68903	0.05134
36.89051	0.01
37.09195	0.00602
37.29242	0.00617
37.4939	0.00671
37.69337	0.00694
37.89385	0.00648
38.09329	0.00661
38.2938	0.00716
38.49321	0.00655

38.69473	0.00671
38.89415	0.00645
39.0946	0.00677
39.29409	0.00643
39.49457	0.0061
39.69506	0.00602
39.89546	0.00591
40.09593	0.00551
40.29641	0.00531
40.49691	0.00564
40.69633	0.00539
40.89679	0.00547
41.09631	0.00513
41.29772	0.00567
41.49719	0.00591
41.69771	0.00596
41.89816	0.00659
42.0986	0.00626
42.29907	0.00536
42.50051	0.0058
42.70097	0.00576
42.90143	0.00682
43.1029	0.00911
43.30339	0.01818
43.50383	0.03132
43.70429	0.05185
43.90578	0.05672
44.10522	0.06164
44.30668	0.06172
44.50717	0.06126
44.70866	0.06148
44.90808	0.06171
45.10854	0.06189
45.30902	0.06177
45.5085	0.06252
45.71	0.06183
45.9104	0.06159
46.11187	0.06169
46.31133	0.06163
46.51183	0.06118
46.71226	0.06031
46.91275	0.06167

47.11319	0.06119
47.31365	0.06075
47.51315	0.06136
47.71361	0.06088
47.9141	0.06154
48.11559	0.06119
48.31698	0.06042
48.51747	0.06044
48.7179	0.06073
48.9174	0.06018
49.11886	0.0604
49.31932	0.06045
49.52076	0.06044
49.72025	0.06059
49.92169	0.05972
50.12215	0.05948
50.32262	0.05947
50.52208	0.05908
50.72258	0.05685
50.92301	0.01711
51.12248	0.00767
51.32296	0.00577
51.52341	0.00552
51.72487	0.00604
51.92435	0.0061
52.1248	0.00653
52.32428	0.00658
52.5248	0.00648
52.72423	0.00648
52.92472	0.00707
53.12416	0.00721
53.3256	0.0075
53.52606	0.00788
53.72653	0.00855
53.92799	0.0085
54.12845	0.0087
54.32898	0.00748
54.52839	0.0082
54.72985	0.00798
54.93036	0.00795
55.13078	0.00838
55.33027	0.00837

55.53171	0.00852
55.7322	0.00869
55.9327	0.00843
56.13313	0.00831
56.33362	0.00786
56.53406	0.00809
56.73449	0.00774
56.93596	0.00738
57.13645	0.00739
57.3369	0.0074
57.53635	0.0068
57.73684	0.00673
57.93628	0.00707
58.13681	0.00667
58.33727	0.00644
58.53868	0.00667
58.73917	0.0083
58.93964	0.01372
59.14007	0.02545
59.34054	0.03857
59.54202	0.04582
59.74146	0.05475
59.94195	0.06758
60.14239	0.06759
60.34292	0.06752
60.54239	0.06723
60.74279	0.06712
60.94325	0.06707
61.14378	0.06745
61.34421	0.06693
61.54565	0.06745
61.74611	0.06706
61.94757	0.06756
62.14904	0.06723
62.3485	0.06739
62.54903	0.06723
62.74849	0.06709
62.94992	0.06733
63.14936	0.06759
63.35083	0.06771
63.55129	0.06776
63.75275	0.06779

63.95224	0.0676
64.15271	0.06754
64.35215	0.06786
64.55264	0.06717
64.75408	0.06684
64.95354	0.0664
65.15401	0.06637
65.35348	0.0662
65.55397	0.0664
65.75341	0.06607
65.95487	0.06626
66.15434	0.06649
66.3558	0.06642
66.55527	0.0668
66.75673	0.0549
66.9572	0.0158
67.15769	0.00601
67.35715	0.00421
67.55761	0.00343
67.75712	0.00353
67.95858	0.00329
68.15901	0.00291
68.35848	0.00265
68.55894	0.00203
68.75844	0.00221
68.95885	0.00259
69.15934	0.00287
69.36078	0.00289
69.56029	0.00259
69.76176	0.00315
69.96217	0.00342
70.16265	0.00262
70.3631	0.0024
70.56356	0.00277
70.76403	0.00308
70.96356	0.00324
71.16499	0.00306
71.36545	0.00338
71.56694	0.00332
71.76738	0.00359
71.96782	0.00347
72.1683	0.00385

72.36977	0.0039
72.5702	0.00373
72.77068	0.00429
72.97113	0.00471
73.17163	0.00521
73.37312	0.00522
73.57355	0.00538
73.77505	0.00581
73.97448	0.00594
74.17495	0.00604
74.37442	0.00617
74.57587	0.00617
74.77631	0.00653
74.97778	0.00671
75.17724	0.00631
75.37771	0.00625
75.57718	0.00648
75.77866	0.00687
75.97814	0.00627
76.17857	0.00556
76.37904	0.00538
76.57955	0.00532
76.78102	0.00558
76.98046	0.00503
77.18089	0.00487
77.38038	0.0051
77.58082	0.00461
77.78032	0.00505
77.98175	0.00521
78.18128	0.00532
78.38271	0.00535
78.58218	0.00516
78.78262	0.00519
78.98408	0.00526
79.18354	0.00463
79.38403	0.00448
79.58447	0.00494
79.78507	0.00451
79.98441	0.00412
80.18487	0.00372
80.38534	0.00323
80.58686	0.00292

80.78726	0.00237
80.98872	0.0026
81.18819	0.00394
81.38868	0.00768
81.58913	0.01436
81.78862	0.03682
81.98905	0.0519
82.18952	0.05806
82.38998	0.05788
82.59048	0.05805
82.79092	0.05892
82.99041	0.05835
83.19088	0.05947
83.39034	0.05953
83.5908	0.05901
83.79024	0.05854
83.99071	0.05869
84.19018	0.05868
84.39164	0.05941
84.5931	0.05961
84.79262	0.05897
84.99309	0.05857
85.1925	0.0585
85.39396	0.05885
85.59343	0.05853
85.79395	0.05856
85.99342	0.05834
86.19388	0.05827
86.39332	0.05846
86.59379	0.05814
86.79425	0.05761
86.99471	0.05794
87.19515	0.05819
87.39561	0.05775
87.59613	0.05797
87.7956	0.05805
87.99707	0.0586
88.1965	0.05877
88.39694	0.05852
88.59743	0.05824
88.79893	0.05849
88.99839	0.05794

89.19883	0.05845
89.3983	0.05779
89.59973	0.05775
89.80022	0.04163
90.00068	0.00814
90.20215	0.00244
90.40159	0.00149
90.60205	0.00135
90.80154	0.0014
91.00204	0.00163
91.20151	6.37E-04
91.40194	8.80E-04
91.60238	4.85E-04
91.80285	7.48E-05
92.00234	4.70E-04
92.20278	4.03E-04
92.40324	1.86E-04
92.60477	0
92.80523	0
93.00563	3.22E-05
93.20715	6.01E-04
93.40669	5.83E-04
93.60802	5.71E-04
93.80852	7.40E-04
94.00995	0.00102
94.20942	0.00104
94.41088	7.60E-04
94.61037	8.21E-04
94.81184	8.26E-04
95.01128	6.60E-04
95.21177	4.61E-04
95.41221	0.00136
95.61267	9.46E-04
95.81314	0.00104
96.01263	0.00137
96.21308	0.00115
96.41353	0.00132
96.614	0.00149
96.81347	0.00153
97.01393	0.00185
97.2134	0.00196
97.41386	0.00167

97.61333	0.00172
97.81379	0.00196
98.01326	0.00289
98.21375	0.00235
98.41419	0.00245
98.61366	0.00255
98.81412	0.002
99.01463	0.00289
99.21505	0.00265
99.41453	0.00453
99.61501	0.01119
99.81545	0.03103
100.01594	0.06117
100.21538	0.07119
100.41586	0.07209
100.61633	0.07205
100.81778	0.0717
101.01824	0.07242
101.2197	0.07259
101.42119	0.0727
101.62065	0.07301
101.8221	0.07258
102.02157	0.07319
102.22205	0.07339
102.42252	0.07296
102.62395	0.07345
102.82341	0.07442
103.02388	0.07397
103.2244	0.07419
103.42593	0.07393
103.62527	0.07433
103.82673	0.07389
104.0282	0.07458
104.22866	0.0742
104.42919	0.07298
104.62862	0.06432
104.83006	0.0644
105.03052	0.06453
105.231	0.0639
105.43048	0.06422
105.63191	0.0618
105.83139	0.01557

106.03185	0.0078
106.23134	0.00731
106.43278	0.00734
106.63326	0.00735
106.83473	0.00766
107.0352	0.00798
107.23563	0.00821
107.4371	0.00741
107.63657	0.00739
107.83802	0.00762
108.03849	0.00749
108.23995	0.00775
108.44046	0.0076
108.64088	0.00812
108.8404	0.00811
109.04186	0.00875
109.24227	0.00864
109.44274	0.00851
109.6442	0.00866
109.84466	0.00887
110.04613	0.00905
110.24559	0.00925
110.44706	0.00888
110.64659	0.00925
110.84702	0.00928
111.04745	0.00967
111.24794	0.01003

111.44838	0.00976
111.64887	0.01033
111.84931	0.01046
112.04977	0.01085
112.25025	0.01111
112.45173	0.01057
112.65218	0.0107
112.85166	0.01042
113.05215	0.01042
113.25256	0.01033
113.45303	0.0103
113.6535	0.01033
113.85396	0.01062
114.05445	0.01032
114.25591	0.0103
114.45635	0.01008
114.65682	0.01061
114.85631	0.01031
115.05675	0.01005
115.25627	0.01025
115.45768	0.0101
115.65817	0.01026
115.8586	0.0101
116.06007	0.01032
116.25954	0.0105
116.46	0.01055
116.65947	0.01071

116.86	0.01094
117.06039	0.01033
117.26098	0.01116
117.46033	0.01147
117.66182	0.01036
117.86131	0.01045
118.06175	0.01039
118.26223	0.01086
118.46265	0.01122
118.66315	0.01143
118.86358	0.01127
119.06404	0.01124
119.26451	0.01122
119.46597	0.01132
119.66545	0.0109
119.86591	0.01011
120.0654	0.01021
120.26583	0.00957
120.46633	0.00905
120.66778	0.00929
120.86829	0.00876
121.06869	0.00881
121.27015	0.00874
121.46968	0.00854
121.67008	0.00883
121.87055	0.00876
122.07102	0.00928

122.27148	0.00769
122.47194	0.00783
122.67144	0.00705
122.87187	0.00681
123.07237	0.00587
123.2738	0.00559
123.47427	0.00493
123.67377	0.00445
123.87422	0.00421
124.07466	0.00351
124.27613	0.00389
124.47559	0.00317
124.67606	0.00304
124.87653	0.00318
125.07701	0.003
125.27749	0.00296
125.47797	0.00259
125.67738	0.00252
125.87791	0.00256
126.07734	0.00272
126.27781	0.00314
126.47824	0.00208
126.67871	0.00234
126.88023	0.00227
127.07965	0.00211
127.2811	0.00265
127.48158	0.00272

127.68305	0.00207
127.88349	0.00266
128.08495	0.0031
128.28442	0.00305
128.48491	0.00293
128.68441	0.00311
128.88581	0.00267
129.08728	0.00272
129.28787	0.00192
129.48823	0.00183
129.6877	0.00199
129.88916	0.00176
130.08863	0.00137
130.2891	0.00171
130.48953	0.00133
130.69003	0.00145
130.8896	0.00122
131.08993	0.00133
131.2904	9.48E-04
131.49188	8.95E-04
131.69232	8.90E-04
131.89385	7.36E-04
132.09425	7.37E-04
132.29473	5.11E-04
132.49617	0

Data/Fig. 1k:

Time (s)	MR (%)
0	1.5408
0.20046	1.53776
0.40192	1.536
0.60239	1.54563
0.80385	1.54411
1.00437	1.55453
1.20492	1.54437
1.40427	1.54089
1.60471	1.54384
1.8042	1.55781
2.00464	1.54345
2.20511	1.54572

2.40457	1.53824
2.60603	1.54411
2.8055	1.54557
3.00602	1.54985
3.20556	1.53999
3.40689	1.54053
3.60738	1.55198
3.80882	1.54151
4.00928	1.5389
4.20981	1.54848
4.40934	1.54407
4.6097	1.55167
4.81114	1.53843

5.01061	1.54222
5.21112	1.55336
5.4106	1.53066
5.61104	1.551
5.81047	1.5392
6.01193	1.55375
6.21142	1.54638
6.41286	1.54562
6.61332	1.54347
6.81481	1.54721
7.01428	1.53986
7.21572	1.55003
7.41524	1.5406

7.61667	1.54825
7.81718	1.53873
8.01663	1.54308
8.21806	1.55457
8.41753	1.54167
8.61902	1.55159
8.8185	1.54603
9.0189	1.54879
9.21836	1.55106
9.41887	1.55656
9.61929	1.54347
9.81978	1.54533
10.01924	1.54411

10.22068	1.55702
10.42215	1.53759
10.62261	1.5486
10.82407	1.55062
11.02455	1.55537
11.226	1.54414
11.42548	1.55447
11.62693	1.55038
11.82739	1.53962
12.02885	1.54735
12.22933	1.53512
12.42978	1.55159
12.62925	1.54297

12.83071	1.53287
13.03123	1.49893
13.23165	1.44073
13.43216	1.34679
13.63259	1.21258
13.83403	0.95659
14.03452	0.61884
14.23496	0.33071
14.43542	0.18632
14.63689	0.15463
14.83635	0.16584
15.03783	0.15507
15.23728	0.15628
15.43777	0.14025
15.63723	0.14229
15.83867	0.14276
16.03917	0.13251
16.23866	0.13293
16.43913	0.14064
16.63856	0.13764
16.839	0.14191
17.03846	0.12523
17.23995	0.1324
17.43939	0.13397
17.64085	0.13609
17.84132	0.13484
18.04178	0.12767
18.24227	0.13874
18.44273	0.13136
18.64417	0.13246
18.84464	0.46228
19.0451	0.88536
19.24557	1.28124
19.44708	1.4401
19.64649	1.48945
19.84801	1.49989
20.04842	1.51208
20.24888	1.50898
20.44935	1.51458
20.64984	1.50726
20.85028	1.52009
21.05177	1.50638

21.2522	1.51235
21.45268	1.51418
21.65413	1.51964
21.8546	1.51179
22.05506	1.51544
22.25455	1.51592
22.45602	1.50966
22.65545	1.51469
22.85694	1.51938
23.05738	1.52511
23.2579	1.51902
23.45733	1.51777
23.65777	1.51623
23.85724	1.52646
24.05771	1.51761
24.25817	1.51614
24.45764	1.53117
24.6591	1.52395
24.85959	1.53424
25.06003	1.51933
25.25949	1.4961
25.46098	1.47347
25.66042	1.43731
25.86188	1.4015
26.06137	1.30054
26.26183	1.06659
26.4623	0.83218
26.66377	0.54021
26.86426	0.28406
27.06473	0.2401
27.26616	0.23985
27.46673	0.23494
27.66812	0.23151
27.86758	0.22722
28.06803	0.21792
28.26748	0.21668
28.46895	0.2165
28.66838	0.21243
28.86885	0.21068
29.06931	0.21677
29.27078	0.20738
29.47024	0.20644

29.67171	0.20376
29.87217	0.2106
30.07263	0.20605
30.27409	0.21532
30.47356	0.21466
30.67508	0.20675
30.87449	0.20582
31.07496	0.20653
31.27542	0.19775
31.47688	0.19724
31.67635	0.20482
31.87681	0.18725
32.07634	0.19809
32.27674	0.20565
32.4782	0.26244
32.67867	0.90422
32.88013	1.29671
33.07961	1.43639
33.28006	1.48392
33.47955	1.50861
33.68099	1.51521
33.88149	1.52143
34.08291	1.52222
34.28338	1.53021
34.48484	1.53171
34.68433	1.52132
34.88477	1.52365
35.0843	1.52953
35.28473	1.53714
35.48522	1.52524
35.68467	1.52783
35.88612	1.53171
36.08656	1.52878
36.28703	1.52882
36.48749	1.52533
36.68798	1.53078
36.88842	1.52762
37.08891	1.52608
37.28835	1.52942
37.48884	1.52871
37.68831	1.53498
37.88976	1.52577

38.09021	1.52819
38.28967	1.52212
38.49015	1.5328
38.68961	1.53472
38.89007	1.52914
39.09053	1.53565
39.29199	1.53398
39.49146	1.5351
39.69292	1.52322
39.8934	1.5289
40.09388	1.52626
40.29338	1.53624
40.49482	1.53163
40.69425	1.53935
40.89471	1.53788
41.09524	1.52836
41.29564	1.51561
41.4971	1.49098
41.69757	1.44977
41.89904	1.39654
42.0985	1.29278
42.29896	1.11332
42.49942	0.81388
42.6999	0.48318
42.89936	0.22184
43.09982	0.12788
43.29929	0.11514
43.49978	0.11964
43.70021	0.09928
43.89974	0.10246
44.10017	0.08984
44.29961	0.09696
44.5001	0.09101
44.70054	0.08452
44.901	0.08155
45.10047	0.06937
45.30195	0.08564
45.50141	0.0801
45.70288	0.07598
45.90239	0.08898
46.10379	0.10023
46.30428	0.10229

46.50474	0.10901
46.70618	0.10993
46.9067	0.11481
47.10714	0.11009
47.30757	0.11706
47.50905	0.09826
47.70853	0.10338
47.90996	0.10807
48.11043	0.10545
48.31189	0.10627
48.51139	0.10256
48.71282	0.11401
48.91228	0.11272
49.11377	0.111
49.31521	0.10561
49.51467	0.10653
49.71616	0.1034
49.91561	0.10473
50.11706	0.29932
50.31667	0.82868
50.517	1.28918
50.71649	1.46647
50.91792	1.4957
51.11844	1.5105
51.31988	1.52429
51.51935	1.52423
51.72078	1.52961
51.92127	1.52623
52.12283	1.52186
52.32317	1.52111
52.52266	1.53205
52.7231	1.53339
52.92257	1.53156
53.12403	1.52779
53.3235	1.53146
53.52402	1.53546
53.72445	1.52771
53.9259	1.5285
54.12537	1.5335
54.32585	1.52878
54.52528	1.53696
54.72576	1.53355

54.92621	1.53056
55.12573	1.52593
55.32615	1.53022
55.52661	1.53623
55.72711	1.53346
55.92655	1.5336
56.12706	1.53758
56.32647	1.53769
56.52698	1.53797
56.7274	1.53808
56.92789	1.53221
57.12739	1.53728
57.32779	1.52828
57.52927	1.53
57.73074	1.53567
57.93121	1.53717
58.13066	1.52974
58.33114	1.53755
58.53059	1.53764
58.73106	1.53464
58.93054	1.53538
59.13203	1.54391
59.33244	1.53608
59.53293	1.54056
59.73337	1.53502
59.93383	1.52077
60.13432	1.49799
60.33576	1.4565
60.53724	1.37882
60.73671	1.21332
60.93817	0.99364
61.13761	0.65316
61.33808	0.38556
61.5376	0.26479
61.73801	0.21953
61.93749	0.2055
62.13897	0.20558
62.3384	0.17272
62.53987	0.1256
62.7394	0.08178
62.9398	0.0589
63.14029	0.04901

63.34075	0.04045
63.54125	0.0523
63.74168	0.03414
63.94212	0.04286
64.1426	0.04136
64.34406	0.03356
64.54454	0.02411
64.74602	0.03129
64.94644	0.02301
65.14792	0.02566
65.34739	0.02865
65.54783	0.0303
65.74832	0.01863
65.94876	0.02496
66.15022	0.02567
66.34969	0.02264
66.55019	0.00964
66.74962	0.01893
66.9511	0.01241
67.15055	0.01993
67.35103	0.00733
67.55148	0.01801
67.75293	0.0145
67.95246	0.01539
68.15387	0.01285
68.35433	0.02237
68.55579	0.01223
68.75628	0.00783
68.95572	0.00324
69.15624	0.00918
69.35665	0.00609
69.55711	0.01009
69.75761	0.00975
69.95806	0.00827
70.15751	0.01364
70.358	0.01377
70.55844	0.00109
70.75891	0.00307
70.95939	0.0105
71.15983	0
71.36035	0.32068
71.56178	0.99641

71.76322	1.34176
71.96268	1.40387
72.16321	1.43232
72.36361	1.46154
72.56408	1.46155
72.76357	1.464
72.96405	1.46719
73.16348	1.47638
73.36496	1.48508
73.56441	1.48696
73.7649	1.48872
73.96537	1.49837
74.16586	1.48734
74.36629	1.49172
74.56775	1.49041
74.76921	1.48492
74.96966	1.49913
75.17117	1.48884
75.37058	1.49064
75.57204	1.48608
75.7725	1.49082
75.97396	1.48568
76.17346	1.48631
76.37489	1.48025
76.57436	1.47626
76.77583	1.49487
76.9764	1.46865
77.17675	1.49663
77.37821	1.49034
77.57768	1.48528
77.77914	1.49102
77.9796	1.48826
78.18107	1.49387
78.38053	1.48622
78.582	1.48941
78.78146	1.4966
78.98292	1.48492
79.18239	1.49846
79.38385	1.49424
79.58332	1.50271
79.78378	1.48981
79.98524	1.49975

80.18471	1.50063
80.38518	1.49671
80.58464	1.49038
80.7861	1.49774
80.98557	1.49849
81.18704	1.48833
81.3865	1.49128
81.58796	1.49371
81.78744	1.49493
81.98889	1.49738
82.18836	1.50486
82.38982	1.50343
82.59128	1.49047
82.79174	1.49121
82.99321	1.49717
83.19267	1.48582
83.39414	1.49115
83.5936	1.48626
83.79506	1.46541
83.99553	1.41917
84.19599	1.32485
84.39646	1.09246
84.59692	0.7991
84.79738	0.48526
84.99885	0.37401
85.19833	0.35646
85.39978	0.33903
85.60024	0.33649
85.8007	0.3298
86.00117	0.31151
86.20163	0.25536
86.4021	0.23664
86.60256	0.23588
86.80402	0.22995
87.00449	0.19324
87.20495	0.15403
87.40442	0.14624
87.60489	0.14833
87.80535	0.13882
88.00581	0.14597
88.20628	0.12802
88.40674	0.05386

88.60821	0.03658
88.80767	0.03727
89.00813	0.04153
89.2076	0.03647
89.40906	0.02707
89.60953	0.02029
89.80999	0.02239
90.00946	0.01638
90.20992	0.01467
90.40939	0.02258
90.60985	0.01145
90.80932	0.02141
91.01078	0
91.21125	0
91.41071	0
91.61118	0.00719
91.81064	0.00879
92.01211	0.00508
92.21157	0.00581
92.41303	0.01994
92.6125	0.02882
92.81296	0.01607
93.01244	0.0169
93.2129	0.0184
93.41336	0.01463
93.61383	0.01838
93.81429	0.01814
94.01376	0.01831
94.21522	0.0182
94.41468	0.02228
94.61515	0.01498
94.81561	0.01474
95.01608	0.0212
95.21655	0.01297
95.418	0.01861
95.61747	0.02173
95.81894	0.01757
96.0184	0.0118
96.21986	0.00975
96.42032	0.01111
96.62179	0.01152
96.82325	0.01679

97.02271	0.01373
97.22418	0.02086
97.42464	0.01681
97.6261	0.01989
97.82657	0
98.02803	0.01212
98.22749	0.01078
98.42896	0.01733
98.62942	0.01225
98.82989	0.04455
99.02935	0.61449
99.23081	1.13078
99.43228	1.34596
99.63174	1.40703
99.8332	1.43171
100.0337	1.43511
100.2341	1.44698
100.4336	1.45543
100.6351	1.45791
100.8355	1.45461
101.036	1.46405
101.2355	1.46154
101.4369	1.45932

101.6374	1.45398
101.8388	1.45916
102.0393	1.46594
102.2388	1.45824
102.4402	1.46096
102.6397	1.46417
102.8402	1.46337
103.0397	1.46495
103.2401	1.45806
103.4406	1.46747
103.642	1.46854
103.8415	1.47162
104.042	1.46486
104.2414	1.46561
104.4429	1.46884
104.6424	1.47344
104.8428	1.46521
105.0433	1.47836
105.2428	1.4665
105.4442	1.46871
105.6437	1.46534
105.8441	1.46586
106.0446	1.47228

106.2461	1.46754
106.4455	1.46484
106.646	1.48385
106.8455	1.46784
107.0459	1.46752
107.2454	1.47451
107.4469	1.47549
107.6473	1.47484
107.8468	1.45897
108.0483	1.47081
108.2477	1.47568
108.4492	1.46016
108.6496	1.47724
108.8511	1.47176
109.0516	1.46415
109.252	1.47102
109.4515	1.4728
109.653	1.46978
109.8534	1.47276
110.0549	1.47107
110.2544	1.47411
110.4548	1.47629
110.6553	1.47531

110.8548	1.47297
111.0552	1.46893
111.2557	1.47813
111.4572	1.48002
111.6576	1.47945
111.8591	1.47617
112.0585	1.472
112.259	1.47202
112.4595	1.47515
112.6609	1.48536
112.8604	1.46281
113.0619	1.43866
113.2623	1.37556
113.4628	1.22073
113.6632	0.9441
113.8637	0.57466
114.0642	0.23736
114.2646	0.10754
114.4661	0.0883
114.6666	0.08361
114.868	0.06829
115.0685	0.06166
115.269	0.06165

115.4684	0.05165
115.6689	0.04855
115.8694	0.04301
116.0708	0.0389
116.2723	0.03492
116.4717	0.02444
116.6722	0.02164
116.8727	0.01729
117.0741	0.02407
117.2746	0.01558
117.4751	0.02077
117.6745	0.01646
117.8751	0.01996
118.0745	0.00728
118.275	0.0116
118.4754	0.01348
118.6759	0.01221
118.8774	0.00336
119.0778	0.01172
119.2782	0.00444
119.4777	0.0031

Data/Fig. 2a:

Magnetic field (mT)	MR (%)
94.91328	0.0531
93.84174	0.05725
92.74648	0.0621
91.77263	0.06454
90.69894	0.07025
89.69698	0.07716
88.67176	0.08169
87.67789	0.09098
86.65827	0.09496
85.61667	0.1021
84.6386	0.10409
83.64674	0.10945
82.56681	0.12046
81.63532	0.12457
80.57959	0.13373

79.56911	0.13772
78.56959	0.14462
77.56226	0.15825
76.57551	0.15757
75.52825	0.16895
74.60259	0.17649
73.51663	0.18217
72.52235	0.19171
71.52782	0.19673
70.52908	0.20441
69.52781	0.21982
68.45425	0.22305
67.49792	0.23521
66.47658	0.24236
65.47705	0.25117
64.46086	0.26297

63.47186	0.27117
62.44425	0.28604
61.44143	0.29699
60.42169	0.30552
59.42412	0.32067
58.37138	0.33479
57.41741	0.34511
56.35039	0.35542
55.37626	0.36969
54.37246	0.38264
53.38442	0.39831
52.39028	0.41375
51.32894	0.42894
50.39154	0.44557
49.30312	0.46286
48.29991	0.48283

47.30071	0.49581
46.28111	0.52044
45.36701	0.53877
44.28949	0.56279
43.34312	0.58519
42.28134	0.61133
41.3022	0.63742
40.30518	0.65729
39.27316	0.68657
38.32643	0.71463
37.25312	0.73924
36.29543	0.77031
35.24366	0.80398
34.30502	0.83843
33.24622	0.87479
32.32366	0.91166

31.2331	0.94901
30.28981	0.99593
29.23316	1.03854
28.26003	1.08636
27.20643	1.14322
26.22497	1.19661
25.18263	1.25358
24.23332	1.3112
23.21996	1.37647
22.22268	1.44862
21.16697	1.52306
20.22804	1.59963
19.17852	1.68048
18.19256	1.7681
17.19097	1.86271
16.18679	1.96094

15.19185	2.06553
14.15313	2.17421
13.18802	2.29161
12.13127	2.41721
11.20356	2.54732
10.15254	2.68417
9.1981	2.82308
8.14441	2.96994
7.16114	3.13205
6.16572	3.29635
5.11887	3.45614
4.1882	3.62591
3.14318	3.78334
2.15739	3.92448
1.16711	4.07596
0.12025	4.21976
-0.86747	4.36921
-1.85261	4.51182
-2.84023	4.62295
-3.82264	4.68695
-4.89034	4.70043
-5.82413	4.6884
-6.88228	4.64274
-7.82715	4.57532
-8.87448	4.48455
-9.81302	4.34236
-10.88131	4.1747
-11.8799	4.00414
-12.86959	3.8235
-13.85867	3.64721
-14.83462	3.46344
-15.90185	3.26832
-16.84665	3.08264
-17.89549	2.92189
-18.8793	2.75862
-19.89355	2.5834
-20.89332	2.42562
-21.8979	2.27636
-22.89362	2.14082
-23.89086	2.02014
-24.9048	1.89244
-25.90809	1.77754

-26.89394	1.65285
-27.83889	1.55139
-28.9143	1.44792
-29.90516	1.36282
-30.89574	1.28326
-31.90295	1.21364
-32.90166	1.15184
-33.94238	1.08164
-34.87444	1.0191
-35.93638	0.96293
-36.8811	0.91036
-37.93416	0.86148
-38.91741	0.8183
-39.89069	0.77697
-40.96503	0.73905
-41.91732	0.70492
-42.96874	0.67039
-43.90244	0.63806
-44.93023	0.60852
-45.91105	0.5776
-46.92092	0.55451
-47.90313	0.52873
-48.95232	0.51053
-49.94109	0.48703
-50.97989	0.46798
-51.92406	0.44835
-52.9746	0.43191
-53.90357	0.41441
-54.95918	0.39713
-55.94522	0.37601
-56.92342	0.36745
-57.96401	0.34854
-58.92549	0.33662
-59.98557	0.31833
-60.96742	0.30991
-61.96426	0.30088
-62.9415	0.28407
-63.93495	0.27537
-64.99246	0.26421
-65.9444	0.25462
-67.0003	0.23837
-67.94411	0.23064

-69.011	0.22158
-69.98408	0.21134
-70.96863	0.20595
-71.94915	0.19631
-72.92252	0.18582
-73.99645	0.18221
-74.97928	0.17195
-75.97463	0.16782
-76.97249	0.16094
-77.95198	0.152
-79.01227	0.14714
-80.0025	0.13595
-81.00068	0.13109
-82.03231	0.1205
-83.00768	0.11795
-83.97143	0.11315
-85.03984	0.10585
-85.98348	0.10047
-87.07201	0.09383
-88.01358	0.08957
-89.01087	0.08722
-90.00905	0.08065
-91.07657	0.07482
-91.98087	0.07161
-93.03716	0.06487
-94.02625	0.06036
-94.98283	0.05714
-94.32591	0.06
-93.50407	0.06764
-92.48922	0.0702
-91.43936	0.07646
-90.39576	0.0801
-89.29465	0.08629
-88.22885	0.0924
-87.07532	0.0976
-86.08343	0.10309
-84.94576	0.10703
-83.96178	0.11982
-82.85066	0.12558
-81.83011	0.12885
-80.79027	0.13383
-79.78564	0.14103

-78.75853	0.14833
-77.69509	0.15487
-76.75363	0.16395
-75.64795	0.17047
-74.67775	0.17401
-73.71823	0.18333
-72.69534	0.1959
-71.62183	0.19873
-70.63496	0.20535
-69.69708	0.21525
-68.62259	0.22478
-67.62669	0.23185
-66.56674	0.24223
-65.54671	0.25633
-64.56345	0.26368
-63.5542	0.27196
-62.60118	0.29037
-61.51572	0.30017
-60.50289	0.31009
-59.50718	0.32026
-58.55504	0.33229
-57.47793	0.34485
-56.46869	0.35721
-55.45901	0.37277
-54.44607	0.38505
-53.44912	0.39943
-52.39102	0.41367
-51.42972	0.43064
-50.42557	0.44571
-49.41855	0.46442
-48.3976	0.47914
-47.40983	0.50134
-46.35972	0.5198
-45.36527	0.5388
-44.35619	0.558
-43.35969	0.5823
-42.38787	0.60276
-41.32114	0.62697
-40.33489	0.6509
-39.33019	0.67753
-38.31908	0.70552
-37.32284	0.73523

-36.37809	0.76494
-35.29982	0.79516
-34.32049	0.82926
-33.3316	0.86438
-32.25522	0.90476
-31.30458	0.94253
-30.24724	0.98617
-29.26955	1.02871
-28.27173	1.0765
-27.26784	1.12662
-26.26051	1.18028
-25.25685	1.23591
-24.27253	1.2929
-23.21878	1.35637
-22.25388	1.42344
-21.19958	1.49784
-20.24019	1.57741
-19.17544	1.65645
-18.24564	1.74098
-17.18248	1.83634
-16.22301	1.93561
-15.16497	2.03927
-14.24358	2.15036
-13.16477	2.26363
-12.16562	2.39018
-11.17853	2.52479
-10.19391	2.66052
-9.17174	2.80265
-8.14022	2.95288
-7.17553	3.11917
-6.15573	3.28814
-5.18054	3.46055
-4.13278	3.62795
-3.1827	3.79231
-2.12381	3.93678
-1.13002	4.09741
-0.13785	4.24386
0.9133	4.39892
1.83635	4.54511
2.89153	4.64654
3.89481	4.71349
4.89034	4.73534

5.89181	4.72496
6.88382	4.68202
7.94482	4.60386
8.8865	4.48562
9.93935	4.3535
10.97223	4.19974
11.95982	4.03589
12.96052	3.86081
13.96953	3.66959
14.94474	3.47164
15.99148	3.28158
16.9443	3.10095
17.95501	2.92265
18.96488	2.75065
19.9319	2.58409
21.00434	2.43538
22.0077	2.28076
22.99657	2.14113

23.94833	2.00609
25.02775	1.88094
26.01601	1.77371
27.00566	1.66204
27.98632	1.55494
28.9703	1.45715
30.03485	1.37248
30.99245	1.2904
32.00068	1.21762
33.03637	1.1452
34.04229	1.07928
35.04164	1.01448
36.00521	0.96313
37.04391	0.91095
38.09278	0.86127
39.03829	0.81518
40.03555	0.76693
41.03902	0.73379

42.10322	0.69656
43.03459	0.65993
44.06172	0.63145
45.06133	0.59704
46.08964	0.57268
47.07918	0.54389
48.07579	0.52028
49.08813	0.49586
50.08111	0.46799
51.11874	0.45078
52.10852	0.4298
53.07642	0.41811
54.07637	0.39578
55.07817	0.38045
56.11104	0.36738
57.07129	0.34474
58.11557	0.33654
59.06548	0.31608

60.10743	0.3033
61.04407	0.28987
62.12131	0.28071
63.07644	0.2698
64.14612	0.25952
65.07927	0.24733
66.16156	0.23544
67.10335	0.22448
68.11498	0.21558
69.12271	0.20559
70.07087	0.19895
71.14119	0.18873
72.12301	0.17859
73.12613	0.17094
74.10376	0.16063
75.1029	0.15665
76.11334	0.14784
77.14167	0.14362

78.13895	0.13777
79.1416	0.12946
80.09756	0.11885
81.13042	0.11496
82.13862	0.10985
83.1091	0.10091
84.16039	0.0986
85.0978	0.09122
86.14556	0.08385
87.13978	0.07481
88.13189	0.0714
89.13004	0.06692
90.13743	0.05999
91.11466	0.05311
92.13104	0.04896
93.12065	0.04446
94.16621	0.03745

Data/Fig. 2b:

Time (s)	MR (%)
30.05643	0.4682
29.37502	0.46885
28.681	0.46644
27.95382	0.46711
27.00879	0.46716
26.24791	0.46616
25.47474	0.46641
24.69569	0.46553
23.72096	0.46556
22.92368	0.46434
22.10845	0.45999
21.30723	0.46163
20.34044	0.46033
19.52799	0.45785
18.71162	0.45671
17.91359	0.45744
16.92095	0.45554
16.12825	0.4532
15.30988	0.45037
14.53971	0.44852
13.74106	0.4463

12.85039	0.44144
11.98291	0.43832
11.21898	0.43429
10.42279	0.43035
9.42739	0.4252
8.65314	0.42131
7.88109	0.4144
7.0235	0.40678
6.18513	0.40199
5.43572	0.393
4.65899	0.38354
3.79051	0.37483
2.90881	0.36215
2.13356	0.34767
1.37628	0.32776
0.45669	0.30498
-0.27857	0.2669
-1.02331	0.21951
-1.95148	0.15996
-2.72102	0.08149
-3.48155	0.01639
-4.32618	0.04549

-5.20436	0.13745
-5.93385	0.19645
-6.69771	0.23975
-7.63103	0.27204
-8.3645	0.29387
-9.29737	0.31737
-10.0442	0.33357
-10.7832	0.34245
-11.5639	0.3641
-12.4902	0.37453
-13.2435	0.38379
-13.9958	0.38503
-14.8493	0.39658
-15.6778	0.41014
-16.4262	0.41715
-17.2614	0.42089
-18.0786	0.42169
-18.8285	0.42433
-19.5914	0.42925
-20.5129	0.43261
-21.2601	0.43497
-22.0148	0.43512

-22.9591	0.43786
-23.6916	0.44132
-24.4573	0.44423
-25.3743	0.44584
-26.1141	0.44728
-26.8707	0.44965
-27.7913	0.45227
-28.5158	0.45366
-29.0474	0.45603
-29.4638	0.45555
-29.6145	0.45592
-29.6376	0.45546
-29.49	0.45468
-29.2616	0.45345
-28.9485	0.45211
-28.4122	0.45292
-27.9079	0.45318
-27.2855	0.45135
-26.6161	0.4516
-25.9632	0.44945
-25.2397	0.44869
-24.3938	0.44987

-23.5282	0.44669
-22.74	0.44488
-21.9485	0.44254
-21.1242	0.44098
-20.0842	0.43976
-19.2704	0.43773
-18.4217	0.43453
-17.5756	0.43206
-16.6568	0.4305
-15.7442	0.42771
-14.9046	0.42307
-14.0936	0.4186
-13.108	0.41622
-12.3193	0.41282
-11.4988	0.40852
-10.6819	0.40347
-9.68373	0.39752
-8.88373	0.39307
-8.10605	0.3866
-7.12687	0.38007
-6.34226	0.37176
-5.58493	0.36451

-4.6281	0.35298
-3.86494	0.34099
-3.10934	0.33053
-2.1584	0.31298
-1.37874	0.29491
-0.6023	0.26446
0.18444	0.22541
0.96908	0.17784
1.92176	0.09602
2.71133	0.02249
3.48155	0
4.23522	0.04983
5.081	0.13821
5.96184	0.19826
6.73439	0.26424
7.47866	0.28705
8.41961	0.30513
9.17887	0.33519
9.93606	0.35447
10.86122	0.3686
11.61229	0.38904
12.34522	0.39863
13.19664	0.40401
14.0688	0.41641
14.82359	0.42163
15.60251	0.42631
16.54233	0.43145
17.28784	0.43759
18.04335	0.44106
18.80285	0.44453
19.75507	0.44724
20.52622	0.45086
21.29116	0.45393
22.05341	0.45604
23.01349	0.4609
23.78683	0.46122
24.56325	0.46346
25.3225	0.46406
26.25432	0.46582
27.00757	0.46643
27.75011	0.46741
28.51056	0.46871

Data/Fig. 2c:

Magnetic field (mT)	Sensitivity (T-1)
94.91328	-0.04392
93.84174	-0.03873
92.74648	-0.04423
91.77263	-0.0251
90.69894	-0.05311
89.69698	-0.06894
88.67176	-0.04414
87.67789	-0.09339
86.65827	-0.03895
85.61667	-0.06851
84.6386	-0.02032
83.64674	-0.05394
82.56681	-0.10188
81.63532	-0.04408
80.57959	-0.0866
79.56911	-0.03945
78.56959	-0.06889
77.56226	-0.13515
76.57551	0.00687
75.52825	-0.10848
74.60259	-0.0813
73.51663	-0.0522
72.52235	-0.09579
71.52782	-0.05039
70.52908	-0.07669
69.52781	-0.15362
68.45425	-0.02999
67.49792	-0.12681
66.47658	-0.06983
65.47705	-0.08798
64.46086	-0.11577
63.47186	-0.08269
62.44425	-0.14428
61.44143	-0.10888
60.42169	-0.08344
59.42412	-0.15136
58.37138	-0.13371
57.41741	-0.10776
56.35039	-0.09629
55.37626	-0.14594
54.37246	-0.12855
53.38442	-0.15789
52.39028	-0.1547
51.32894	-0.14254
50.39154	-0.17654
49.30312	-0.15813
48.29991	-0.19816
47.30071	-0.12922
46.28111	-0.24036
45.36701	-0.19943
44.28949	-0.2217
43.34312	-0.23527
42.28134	-0.24467
41.3022	-0.26486
40.30518	-0.19794
39.27316	-0.28179
38.32643	-0.29431
37.25312	-0.22756
36.29543	-0.32199
35.24366	-0.31761
34.30502	-0.36392
33.24622	-0.34045
32.32366	-0.39598
31.2331	-0.33934
30.28981	-0.49248
29.23316	-0.39913
28.26003	-0.48603
27.20643	-0.53358
26.22497	-0.53761
25.18263	-0.53979
24.23332	-0.59912
23.21996	-0.63528
22.22268	-0.71318
21.16697	-0.69449
20.22804	-0.80271
19.17852	-0.75765
18.19256	-0.87325
17.19097	-0.92731
16.18679	-0.95938
15.19185	-1.02995
14.15313	-1.02405
13.18802	-1.18918
12.13127	-1.16048
11.20356	-1.36762
10.15254	-1.26807
9.1981	-1.41546
8.14441	-1.35357
7.16114	-1.59859
6.16572	-1.59784
5.11887	-1.47538
4.1882	-1.76037
3.14318	-1.45155
2.15739	-1.37768
1.16711	-1.46977
0.12025	-1.31804
-0.86747	-1.44969
-1.85261	-1.38519
-2.84023	-1.07548
-3.82264	-0.62233
-4.89034	-0.12055
-5.82413	0.12307
-6.88228	0.41232
-7.82715	0.6824
-8.87448	0.82949
-9.81302	1.45193
-10.8813	1.50654
-11.8799	1.64228
-12.8696	1.75794
-13.8587	1.71971
-14.8346	1.81994
-15.9019	1.77044
-16.8467	1.90652
-17.8955	1.48905
-18.8793	1.6151
-19.8936	1.68404
-20.8933	1.54079
-21.8979	1.4527
-22.8936	1.33267
-23.8909	1.18625
-24.9048	1.23604
-25.9081	1.12517
-26.8939	1.2443
-27.8389	1.05728
-28.9143	0.9484
-29.9052	0.84731
-30.8957	0.79294
-31.903	0.68301
-32.9017	0.61171
-33.9424	0.66728
-34.8744	0.66421
-35.9364	0.52391
-36.8811	0.55142
-37.9342	0.46025
-38.9174	0.43558
-39.8907	0.42135
-40.965	0.35037
-41.9173	0.35594
-42.9687	0.3262
-43.9024	0.34411
-44.9302	0.2856
-45.9111	0.31345
-46.9209	0.22741
-47.9031	0.26114
-48.9523	0.17255
-49.9411	0.2365
-50.9799	0.18258
-51.9241	0.20689
-52.9746	0.15589
-53.9036	0.18756
-54.9592	0.16301
-55.9452	0.21342
-56.9234	0.0872
-57.964	0.18105
-58.9255	0.12361

-59.9856	0.172
-60.9674	0.08547
-61.9643	0.09033
-62.9415	0.17155
-63.935	0.08725
-64.9925	0.10531
-65.9444	0.10044
-67.0003	0.15353
-67.9441	0.08174
-69.011	0.08472
-69.9841	0.105
-70.9686	0.05469
-71.9492	0.09807
-72.9225	0.10762
-73.9965	0.03355
-74.9793	0.10414
-75.9746	0.04144
-76.9725	0.06889
-77.952	0.09107
-79.0123	0.04583
-80.0025	0.11285
-81.0007	0.04863
-82.0323	0.10253
-83.0077	0.02603
-83.9714	0.04977
-85.0398	0.06826
-85.9835	0.05696
-87.072	0.06089
-88.0136	0.04522
-89.0109	0.02361
-90.0091	0.06569
-91.0766	0.05458
-91.9809	0.03553
-93.0372	0.06378
-94.0263	0.04553
-94.9828	0.03363
-94.3259	-0.00278
-93.5041	0.09283
-92.4892	0.02527
-91.4394	0.05952
-90.3958	0.0349
-89.2947	0.05618

-88.2289	0.05724
-87.0753	0.04503
-86.0834	0.05528
-84.9458	0.03464
-83.9618	0.12975
-82.8507	0.05184
-81.8301	0.03194
-80.7903	0.04789
-79.7856	0.0715
-78.7585	0.07096
-77.6951	0.0614
-76.7536	0.09629
-75.648	0.0589
-74.6778	0.0364
-73.7182	0.09694
-72.6953	0.12269
-71.6218	0.02632
-70.635	0.06691
-69.6971	0.10536
-68.6226	0.08851
-67.6267	0.07082
-66.5667	0.09769
-65.5467	0.13782
-64.5635	0.07464
-63.5542	0.08181
-62.6012	0.19261
-61.5157	0.08999
-60.5029	0.0976
-59.5072	0.10186
-58.555	0.12589
-57.4779	0.11626
-56.4687	0.12203
-55.459	0.15349
-54.4461	0.12076
-53.4491	0.14369
-52.391	0.13399
-51.4297	0.17577
-50.4256	0.14944
-49.4186	0.18496
-48.3976	0.14353
-47.4098	0.22363
-46.3597	0.17483

-45.3653	0.19009
-44.3562	0.18916
-43.3597	0.24249
-42.3879	0.20927
-41.3211	0.22551
-40.3349	0.24111
-39.3302	0.26319
-38.3191	0.27492
-37.3228	0.29606
-36.3781	0.31212
-35.2998	0.27803
-34.3205	0.34531
-33.3316	0.35214
-32.2552	0.37178
-31.3046	0.3936
-30.2472	0.40864
-29.2696	0.43076
-28.2717	0.47383
-27.2678	0.49365
-26.2605	0.52646
-25.2569	0.54755
-24.2725	0.57157
-23.2188	0.59424
-22.2539	0.68533
-21.1996	0.69527
-20.2402	0.81652
-19.1754	0.73026
-18.2456	0.89355
-17.1825	0.88076
-16.223	1.01506
-15.165	0.96013
-14.2436	1.18027
-13.1648	1.02673
-12.1656	1.23701
-11.1785	1.33013
-10.1939	1.34276
-9.17174	1.35258
-8.14022	1.41463
-7.17553	1.67159
-6.15573	1.60418
-5.18054	1.70882
-4.13278	1.54172

-3.1827	1.66683
-2.12381	1.31267
-1.13002	1.55266
-0.13785	1.416
0.9133	1.41297
1.83635	1.51491
2.89153	0.91859
3.89481	0.63724
4.89034	0.20959
5.89181	-0.09895
6.88382	-0.41348
7.94482	-0.70427
8.8865	-1.20177
9.93935	-1.20246
10.97223	-1.4287
11.95982	-1.59468
12.96052	-1.68456
13.96953	-1.82803
14.94474	-1.96172
15.99148	-1.75803
16.9443	-1.83873
17.95501	-1.71406
18.96488	-1.65761
19.9319	-1.67899
21.00434	-1.35365
22.0077	-1.50668
22.99657	-1.3824
23.94833	-1.39092
25.02775	-1.13809
26.01601	-1.06606
27.00566	-1.10997
27.98632	-1.07542
28.9703	-0.97956
30.03485	-0.78458
30.99245	-0.84614
32.00068	-0.71318
33.03637	-0.6914
34.04229	-0.64831
35.04164	-0.64192
36.00521	-0.52777
37.04391	-0.49788
38.09278	-0.46955

39.03829	-0.48352
40.03555	-0.48012
41.03902	-0.32786
42.10322	-0.34745
43.03459	-0.39068
44.06172	-0.27557
45.06133	-0.34218
46.08964	-0.23552
47.07918	-0.28939
48.07579	-0.2357
49.08813	-0.24
50.08111	-0.27933
51.11874	-0.16514
52.10852	-0.21109
53.07642	-0.12025
54.07637	-0.22247
55.07817	-0.15246
56.11104	-0.12603
57.07129	-0.23496
58.11557	-0.07824
59.06548	-0.21469
60.10743	-0.12228
61.04407	-0.143
62.12131	-0.08482
63.07644	-0.11387
64.14612	-0.09593
65.07927	-0.13033
66.16156	-0.10955
67.10335	-0.1161
68.11498	-0.08783
69.12271	-0.09892
70.07087	-0.06992
71.14119	-0.09531
72.12301	-0.10311
73.12613	-0.07608
74.10376	-0.10534
75.1029	-0.0397
76.11334	-0.08707
77.14167	-0.04098
78.13895	-0.05859
79.1416	-0.08279
80.09756	-0.11088

81.13042	-0.03759
82.13862	-0.0506
83.1091	-0.09206
84.16039	-0.02198
85.0978	-0.07865
86.14556	-0.07024
87.13978	-0.09084
88.13189	-0.03433
89.13004	-0.0449
90.13743	-0.06875
91.11466	-0.07034
92.13104	-0.04081
93.12065	-0.04545
94.16621	-0.06705

Data/Fig. 2d:

Magnetic field (mT)	Sensitivity (T-1)
27.95382	-0.00923
27.00879	-4.75E-04
26.24791	0.01305
25.47474	-0.00317
24.69569	0.0112
23.72096	-2.47E-04
22.92368	0.01515
22.10845	0.05311
21.30723	-0.02036
20.34044	0.01339
19.52799	0.03035
18.71162	0.01399
17.91359	-0.0091
16.92095	0.01901
16.12825	0.02939
15.30988	0.03442
14.53971	0.02391
13.74106	0.02763
12.85039	0.05438
11.98291	0.03574
11.21898	0.05253
10.42279	0.04932
9.42739	0.05152
8.65314	0.04996
7.88109	0.08924
7.0235	0.08845
6.18513	0.05694
5.43572	0.11942
4.65899	0.12141
3.79051	0.09981
2.90881	0.14335
2.13356	0.18614
1.37628	0.26199
0.45669	0.24699
-0.27857	0.51658
-1.02331	0.63488
-1.95148	0.64054
-2.72102	1.01895
-3.48155	0.85578
-4.32618	-0.34432
-5.20436	-1.04572
-5.93385	-0.80719
-6.69771	-0.56557
-7.63103	-0.34504
-8.3645	-0.29671
-9.29737	-0.25108
-10.0442	-0.2162
-10.7832	-0.11973
-11.5639	-0.27632
-12.4902	-0.11223
-13.2435	-0.12245
-13.9958	-0.01646
-14.8493	-0.13471
-15.6778	-0.16306
-16.4262	-0.09324
-17.2614	-0.04465
-18.0786	-0.00969
-18.8285	-0.03514
-19.5914	-0.06416
-20.5129	-0.03633
-21.2601	-0.03144
-22.0148	-0.00199
-22.9591	-0.02891
-23.6916	-0.047
-24.4573	-0.03783
-25.3743	-0.0175
-26.1141	-0.01928
-26.8707	-0.03118
-27.7913	-0.02835
-27.9079	0.00512
-27.2855	-0.02939
-26.6161	0.0038
-25.9632	-0.03286
-25.2397	-0.01041
-24.3938	0.01393
-23.5282	-0.03664
-22.74	-0.02278
-21.9485	-0.02945
-21.1242	-0.0189
-20.0842	-0.01169
-19.2704	-0.02479
-18.4217	-0.03757
-17.5756	-0.02901
-16.6568	-0.0169
-15.7442	-0.03049
-14.9046	-0.05503
-14.0936	-0.0549
-13.108	-0.024
-12.3193	-0.04292
-11.4988	-0.05221
-10.6819	-0.06158
-9.68373	-0.05938
-8.88373	-0.05541
-8.10605	-0.08287
-7.12687	-0.06641
-6.34226	-0.10554
-5.58493	-0.09538
-4.6281	-0.12007
-3.86494	-0.15656
-3.10934	-0.13807
-2.1584	-0.18393
-1.37874	-0.23107
-0.6023	-0.39116
0.18444	-0.49519
0.96908	-0.60521
1.92176	-0.85805
2.71133	-0.93108
3.48155	-0.29196
4.23522	0.6609
5.081	1.04345
5.96184	0.68037
6.73439	0.85176
7.47866	0.30563
8.41961	0.19157
9.17887	0.39458
9.93606	0.25378
10.86122	0.15214
11.61229	0.27115
12.34522	0.13029
13.19664	0.06294
14.0688	0.14153
14.82359	0.0689
15.60251	0.05983
16.54233	0.05447
17.28784	0.08204
18.04335	0.04573
18.80285	0.04544
19.75507	0.02832
20.52622	0.04672
21.29116	0.04003
22.05341	0.0275
23.01349	0.05041
23.78683	0.00415
24.56325	0.02864
25.3225	0.00782
26.25432	0.01886
27.00757	0.0081
27.75011	0.01307

Data/Fig. 2e:

15.4μA		33.4μA		0.16mA		0.34mA		0.72mA		3.36mA	
Frequency (Hz)	Noise Power Density (Ω/√Hz)	Frequency (Hz)	Noise Power Density (Ω/√Hz)	Frequency (Hz)	Noise Power Density (Ω/√Hz)	Frequency (Hz)	Noise Power Density (Ω/√Hz)	Frequency (Hz)	Noise Power Density (Ω/√Hz)	Frequency (Hz)	Noise Power Density (Ω/√Hz)
0.07726	0.00168	0.07713	0.00129	0.07742	0.00163	0.07707	8.04E-04	0.07809	9.27E-04	0.07729	6.18E-04
0.24895	0.00186	0.24852	0.00113	0.24946	5.53E-04	0.24835	2.95E-04	0.25162	3.05E-04	0.24903	1.96E-04
0.42063	0.00256	0.41991	0.00121	0.4215	2.71E-04	0.41963	2.31E-04	0.42515	1.77E-04	0.42078	1.13E-04
0.59232	0.00202	0.59131	0.00105	0.59354	2.10E-04	0.5909	1.52E-04	0.59868	1.67E-04	0.59252	7.02E-05

0.76401	0.00202	0.7627	0.00119	0.76558	2.65E-04	0.76218	1.14E-04	0.77221	1.05E-04	0.76427	5.36E-05
0.9357	0.00212	0.9341	0.00152	0.93762	2.59E-04	0.93346	1.10E-04	0.94574	1.02E-04	0.93602	4.45E-05
1.10738	0.00249	1.10549	8.72E-04	1.10966	2.62E-04	1.10473	1.15E-04	1.11927	6.77E-05	1.10776	4.22E-05
1.27907	0.0024	1.27688	8.35E-04	1.2817	2.31E-04	1.27601	9.88E-05	1.2928	5.38E-05	1.27951	4.75E-05
1.45076	0.00272	1.44828	0.0011	1.45375	2.34E-04	1.44729	1.15E-04	1.46633	5.58E-05	1.45125	3.71E-05
1.62245	0.00202	1.61967	0.00103	1.62579	1.98E-04	1.61857	1.03E-04	1.63985	6.28E-05	1.623	5.07E-05
1.79413	0.00211	1.79107	0.00103	1.79783	2.01E-04	1.78984	1.32E-04	1.81338	5.58E-05	1.79475	3.65E-05
1.96582	0.00243	1.96246	0.0011	1.96987	2.41E-04	1.96112	9.98E-05	1.98691	5.54E-05	1.96649	4.88E-05
2.13751	0.00194	2.13385	0.00117	2.14191	2.45E-04	2.1324	9.79E-05	2.16044	6.40E-05	2.13824	2.17E-05
2.30919	0.00227	2.30525	9.29E-04	2.31395	2.02E-04	2.30367	8.85E-05	2.33397	4.25E-05	2.30999	2.35E-05
2.48088	0.0017	2.47664	9.91E-04	2.48599	2.29E-04	2.47495	1.25E-04	2.5075	6.51E-05	2.48173	3.38E-05
2.65257	0.00218	2.64803	0.00112	2.65803	1.61E-04	2.64623	1.05E-04	2.68103	6.83E-05	2.65348	3.54E-05
2.82426	0.00201	2.81943	9.06E-04	2.83007	2.17E-04	2.8175	1.15E-04	2.85456	7.25E-05	2.82522	3.46E-05
2.99594	0.0019	2.99082	0.0012	3.00211	1.89E-04	2.98878	1.24E-04	3.02809	5.05E-05	2.99697	2.73E-05
3.16763	0.00233	3.16222	0.00118	3.17415	2.77E-04	3.16006	1.38E-04	3.20162	7.36E-05	3.16872	3.45E-05
3.33932	0.00194	3.33361	0.00108	3.34619	2.45E-04	3.33133	1.02E-04	3.37515	5.58E-05	3.34046	2.79E-05
3.51101	0.00214	3.505	0.00105	3.51824	1.74E-04	3.50261	9.33E-05	3.54868	4.98E-05	3.51221	3.00E-05
3.68269	0.00274	3.6764	8.93E-04	3.69028	2.47E-04	3.67389	6.75E-05	3.72221	6.28E-05	3.68395	2.78E-05
3.85438	0.0023	3.84779	0.00112	3.86232	2.18E-04	3.84516	1.23E-04	3.89574	5.97E-05	3.8557	2.51E-05
4.02607	0.00193	4.01919	0.0012	4.03436	2.18E-04	4.01644	8.22E-05	4.06927	5.79E-05	4.02745	3.55E-05
4.19776	0.00208	4.19058	0.0011	4.2064	2.55E-04	4.18772	1.08E-04	4.2428	5.81E-05	4.19919	2.33E-05
4.36944	0.0025	4.36197	0.00111	4.37844	2.54E-04	4.35899	1.26E-04	4.41633	5.67E-05	4.37094	2.98E-05
4.54113	0.00175	4.53337	0.00143	4.55048	2.31E-04	4.53027	1.08E-04	4.58986	4.29E-05	4.54269	2.41E-05
4.71282	0.00293	4.70476	0.00103	4.72252	2.42E-04	4.70155	6.70E-05	4.76339	5.18E-05	4.71443	3.17E-05
4.8845	0.00191	4.87616	7.38E-04	4.89456	2.53E-04	4.87282	9.81E-05	4.93692	7.28E-05	4.88618	3.04E-05
5.05619	0.0026	5.04755	0.00117	5.0666	2.29E-04	5.0441	9.85E-05	5.11045	4.60E-05	5.05792	2.91E-05
5.22788	0.00233	5.21894	0.00128	5.23864	2.11E-04	5.21538	8.72E-05	5.28398	4.74E-05	5.22967	3.08E-05
5.39957	0.00272	5.39034	0.00108	5.41068	2.92E-04	5.38665	1.07E-04	5.45751	4.14E-05	5.40142	3.08E-05
5.57125	0.00261	5.56173	0.00101	5.58273	1.82E-04	5.55793	1.27E-04	5.63104	4.96E-05	5.57316	2.48E-05
5.74294	0.00261	5.73312	8.60E-04	5.75477	1.80E-04	5.72921	1.21E-04	5.80457	4.49E-05	5.74491	2.58E-05
5.91463	0.00197	5.90452	0.00111	5.92681	2.39E-04	5.90048	1.19E-04	5.97809	4.42E-05	5.91665	2.60E-05
6.08632	0.00217	6.07591	0.00123	6.09885	2.98E-04	6.07176	1.31E-04	6.15162	6.13E-05	6.0884	2.82E-05
6.258	0.00302	6.24731	0.00134	6.27089	1.16E-04	6.24304	1.13E-04	6.32515	5.35E-05	6.26015	2.51E-05
6.42969	0.00219	6.4187	0.00108	6.44293	1.89E-04	6.41431	8.64E-05	6.49868	5.67E-05	6.43189	2.79E-05
6.60138	0.00187	6.59009	8.87E-04	6.61497	1.84E-04	6.58559	1.33E-04	6.67221	4.27E-05	6.60364	2.92E-05
6.77307	0.0021	6.76149	0.00109	6.78701	1.89E-04	6.75687	1.40E-04	6.84574	4.03E-05	6.77539	3.31E-05
6.94475	0.0023	6.93288	0.001	6.95905	1.99E-04	6.92814	8.40E-05	7.01927	4.43E-05	6.94713	2.66E-05
7.11644	0.0023	7.10428	0.00105	7.13109	2.60E-04	7.09942	9.04E-05	7.1928	5.59E-05	7.11888	2.94E-05
7.28813	0.00208	7.27567	0.00132	7.30313	2.27E-04	7.2707	1.27E-04	7.36633	5.84E-05	7.29062	2.81E-05
7.45981	0.00217	7.44706	8.69E-04	7.47517	2.42E-04	7.44197	1.12E-04	7.53986	4.60E-05	7.46237	2.38E-05
7.6315	0.00286	7.61846	0.00113	7.64722	3.22E-04	7.61325	9.91E-05	7.71339	4.00E-05	7.63412	2.09E-05
7.80319	0.00267	7.78985	0.00123	7.81926	2.82E-04	7.78453	1.06E-04	7.88692	5.20E-05	7.80586	2.03E-05

7.97488	0.00203	7.96124	8.78E-04	7.9913	2.17E-04	7.9558	9.94E-05	8.06045	4.97E-05	7.97761	2.30E-05
8.14656	0.00204	8.13264	0.001	8.16334	2.13E-04	8.12708	1.22E-04	8.23398	5.20E-05	8.14935	2.52E-05
8.31825	0.00252	8.30403	9.04E-04	8.33538	2.12E-04	8.29836	1.14E-04	8.40751	5.74E-05	8.3211	2.75E-05
8.48994	0.00174	8.47543	9.33E-04	8.50742	2.05E-04	8.46963	9.92E-05	8.58104	4.47E-05	8.49285	2.71E-05
8.66163	0.00253	8.64682	9.17E-04	8.67946	2.37E-04	8.64091	1.04E-04	8.75457	6.12E-05	8.66459	2.77E-05
8.83331	0.00252	8.81821	0.00109	8.8515	1.73E-04	8.81219	1.47E-04	8.9281	5.33E-05	8.83634	3.14E-05
9.005	0.00293	8.98961	0.0011	9.02354	1.81E-04	8.98346	1.29E-04	9.10163	6.61E-05	9.00809	3.87E-05
9.17669	0.0025	9.161	0.00102	9.19558	1.99E-04	9.15474	1.55E-04	9.27516	6.27E-05	9.17983	2.12E-05
9.34838	0.00189	9.3324	9.72E-04	9.36762	2.23E-04	9.32602	1.21E-04	9.44869	5.52E-05	9.35158	2.81E-05
9.52006	0.00217	9.50379	0.00108	9.53966	1.89E-04	9.4973	9.17E-05	9.62222	3.57E-05	9.52332	2.69E-05
9.69175	0.00246	9.67518	0.00154	9.71171	2.74E-04	9.66857	9.63E-05	9.79575	7.31E-05	9.69507	2.42E-05
9.86344	0.00186	9.84658	8.27E-04	9.88375	2.16E-04	9.83985	1.15E-04	9.96928	6.04E-05	9.86682	1.98E-05
10.03512	0.00219	10.01797	0.00124	10.05579	2.88E-04	10.01113	1.20E-04	10.14281	5.33E-05	10.03856	2.27E-05
10.20681	0.002	10.18936	0.00108	10.22783	1.99E-04	10.1824	8.26E-05	10.31633	6.35E-05	10.21031	1.84E-05
10.3785	0.00257	10.36076	0.00113	10.39987	3.26E-04	10.35368	1.20E-04	10.48986	4.66E-05	10.38205	3.14E-05
10.55019	0.00189	10.53215	8.51E-04	10.57191	2.18E-04	10.52496	1.08E-04	10.66339	4.74E-05	10.5538	2.78E-05
10.72187	0.00241	10.70355	0.00109	10.74395	2.64E-04	10.69623	1.01E-04	10.83692	7.62E-05	10.72555	2.56E-05
10.89356	0.00328	10.87494	8.98E-04	10.91599	2.61E-04	10.86751	1.28E-04	11.01045	4.79E-05	10.89729	2.75E-05
11.06525	0.00263	11.04633	0.00149	11.08803	2.23E-04	11.03879	9.30E-05	11.18398	4.73E-05	11.06904	2.80E-05
11.23694	0.00249	11.21773	8.21E-04	11.26007	2.12E-04	11.21006	1.53E-04	11.35751	4.34E-05	11.24079	2.36E-05
11.40862	0.0021	11.38912	0.00121	11.43211	2.27E-04	11.38134	1.71E-04	11.53104	5.44E-05	11.41253	2.76E-05
11.58031	0.00214	11.56052	0.00107	11.60415	3.09E-04	11.55262	9.68E-05	11.70457	4.98E-05	11.58428	2.90E-05
11.752	0.00182	11.73191	0.00117	11.7762	2.47E-04	11.72389	1.11E-04	11.8781	4.90E-05	11.75602	2.44E-05
11.92369	0.00198	11.9033	0.0011	11.94824	1.77E-04	11.89517	1.09E-04	12.05163	5.22E-05	11.92777	2.43E-05
12.09537	0.00265	12.0747	0.00107	12.12028	2.61E-04	12.06645	1.18E-04	12.22516	4.18E-05	12.09952	3.01E-05
12.26706	0.00184	12.24609	0.0011	12.29232	2.59E-04	12.23772	9.93E-05	12.39869	6.01E-05	12.27126	3.28E-05
12.43875	0.00225	12.41748	6.77E-04	12.46436	2.37E-04	12.409	9.45E-05	12.57222	4.48E-05	12.44301	2.88E-05
12.61043	0.0023	12.58888	8.75E-04	12.6364	2.54E-04	12.58028	1.14E-04	12.74575	4.98E-05	12.61475	2.97E-05
12.78212	0.00222	12.76027	8.86E-04	12.80844	2.27E-04	12.75155	1.31E-04	12.91928	5.28E-05	12.7865	3.61E-05
12.95381	0.00223	12.93167	0.00107	12.98048	2.21E-04	12.92283	1.30E-04	13.09281	3.74E-05	12.95825	2.81E-05
13.1255	0.00263	13.10306	0.00124	13.15252	1.96E-04	13.09411	1.10E-04	13.26634	5.96E-05	13.12999	2.86E-05
13.29718	0.00276	13.27445	9.77E-04	13.32456	2.16E-04	13.26538	1.06E-04	13.43987	6.78E-05	13.30174	3.33E-05
13.46887	0.00244	13.44585	0.00117	13.4966	3.13E-04	13.43666	8.44E-05	13.6134	8.33E-05	13.47349	2.59E-05
13.64056	0.0031	13.61724	0.0012	13.66864	2.93E-04	13.60794	1.15E-04	13.78693	5.11E-05	13.64523	2.01E-05
13.81225	0.00221	13.78864	9.72E-04	13.84069	1.84E-04	13.77921	1.28E-04	13.96046	7.33E-05	13.81698	3.24E-05
13.98393	0.00292	13.96003	0.00111	14.01273	2.77E-04	13.95049	1.06E-04	14.13399	5.76E-05	13.98872	1.96E-05
14.15562	0.00192	14.13142	0.00109	14.18477	3.41E-04	14.12177	1.18E-04	14.30752	4.71E-05	14.16047	2.24E-05
14.32731	0.00223	14.30282	0.00114	14.35681	2.71E-04	14.29304	1.48E-04	14.48105	4.10E-05	14.33222	2.37E-05
14.499	0.00178	14.47421	8.73E-04	14.52885	2.34E-04	14.46432	1.35E-04	14.65457	6.17E-05	14.50396	2.34E-05
14.67068	0.00169	14.6456	0.00137	14.70089	2.35E-04	14.6356	1.36E-04	14.8281	4.42E-05	14.67571	2.31E-05
14.84237	0.00263	14.817	0.0012	14.87293	1.95E-04	14.80687	1.58E-04			14.84745	2.90E-05
		14.98839	9.57E-04			14.97815	1.02E-04				

Data/Fig. 2f:

13.8μA		29.9μA		0.14mA		0.30mA		0.65mA		1.40mA	
Frequency (Hz)	Noise Power Density (Ω/√Hz)	Frequency (Hz)	Noise Power Density (Ω/√Hz)	Frequency (Hz)	Noise Power Density (Ω/√Hz)	Frequency (Hz)	Noise Power Density (Ω/√Hz)	Frequency (Hz)	Noise Power Density (Ω/√Hz)	Frequency (Hz)	Noise Power Density (Ω/√Hz)
0.07517	0.00119	0.07787	6.49E-04	0.08198	9.60E-04	0.07368	5.39E-04	0.07506	8.75E-04	0.07799	3.55E-04
0.2422	0.00116	0.25092	6.01E-04	0.26414	2.14E-04	0.2374	2.32E-04	0.24185	1.32E-04	0.25132	1.65E-04
0.40924	0.00141	0.42396	6.85E-04	0.44631	1.28E-04	0.40113	2.04E-04	0.40864	6.06E-05	0.42464	1.08E-04
0.57628	0.00108	0.59701	5.98E-04	0.62848	1.43E-04	0.56485	1.54E-04	0.57543	4.54E-05	0.59796	6.00E-05
0.74331	0.00125	0.77005	7.03E-04	0.81065	1.55E-04	0.72858	1.08E-04	0.74222	4.24E-05	0.77128	4.23E-05
0.91035	0.0011	0.9431	6.34E-04	0.99282	1.25E-04	0.8923	6.36E-05	0.90901	3.13E-05	0.9446	3.77E-05
1.07739	0.00101	1.11614	5.26E-04	1.17499	1.43E-04	1.05603	6.25E-05	1.0758	3.92E-05	1.11792	3.17E-05
1.24442	0.0013	1.28919	6.43E-04	1.35715	1.58E-04	1.21975	6.93E-05	1.24259	3.97E-05	1.29124	4.00E-05
1.41146	0.00129	1.46224	7.88E-04	1.53932	1.42E-04	1.38348	6.30E-05	1.40938	3.47E-05	1.46456	2.38E-05
1.5785	0.00129	1.63528	5.41E-04	1.72149	1.39E-04	1.5472	6.51E-05	1.57617	3.19E-05	1.63788	2.44E-05
1.74553	9.56E-04	1.80833	6.50E-04	1.90366	1.59E-04	1.71093	5.54E-05	1.74296	2.46E-05	1.8112	2.59E-05
1.91257	0.00132	1.98137	5.58E-04	2.08583	1.49E-04	1.87466	7.06E-05	1.90975	4.04E-05	1.98452	3.18E-05
2.07961	0.00156	2.15442	6.22E-04	2.26799	1.38E-04	2.03838	4.76E-05	2.07654	2.60E-05	2.15784	2.17E-05
2.24664	0.0019	2.32746	5.66E-04	2.45016	8.89E-05	2.20211	5.23E-05	2.24333	3.25E-05	2.33116	2.68E-05
2.41368	0.00104	2.50051	5.11E-04	2.63233	1.20E-04	2.36583	5.58E-05	2.41012	3.46E-05	2.50448	2.36E-05
2.58072	0.00111	2.67356	4.68E-04	2.8145	1.14E-04	2.52956	6.48E-05	2.57691	3.52E-05	2.67781	2.36E-05
2.74776	0.00121	2.8466	6.99E-04	2.99667	1.40E-04	2.69328	5.94E-05	2.7437	3.36E-05	2.85113	1.94E-05
2.91479	0.00138	3.01965	8.35E-04	3.17884	9.23E-05	2.85701	5.44E-05	2.91049	2.59E-05	3.02445	2.05E-05
3.08183	0.00128	3.19269	4.88E-04	3.361	9.89E-05	3.02073	6.62E-05	3.07728	3.34E-05	3.19777	2.65E-05
3.24887	0.00111	3.36574	6.01E-04	3.54317	1.21E-04	3.18446	6.52E-05	3.24407	2.42E-05	3.37109	2.35E-05
3.4159	0.00114	3.53878	6.41E-04	3.72534	1.50E-04	3.34818	7.58E-05	3.41086	3.22E-05	3.54441	2.63E-05
3.58294	0.00145	3.71183	6.61E-04	3.90751	1.13E-04	3.51191	5.01E-05	3.57765	2.52E-05	3.71773	2.49E-05
3.74998	0.00134	3.88488	6.62E-04	4.08968	1.53E-04	3.67563	4.78E-05	3.74444	3.69E-05	3.89105	2.18E-05
3.91701	0.00131	4.05792	5.69E-04	4.27185	1.27E-04	3.83936	5.04E-05	3.91123	2.68E-05	4.06437	2.04E-05
4.08405	0.00121	4.23097	7.63E-04	4.45401	1.33E-04	4.00308	7.22E-05	4.07802	2.40E-05	4.23769	2.27E-05
4.25109	0.0013	4.40401	4.94E-04	4.63618	1.17E-04	4.16681	4.70E-05	4.24481	2.36E-05	4.41101	1.92E-05
4.41812	7.84E-04	4.57706	6.71E-04	4.81835	1.53E-04	4.33054	4.48E-05	4.4116	3.09E-05	4.58433	1.99E-05
4.58516	0.00121	4.7501	5.98E-04	5.00052	1.61E-04	4.49426	5.75E-05	4.57839	2.87E-05	4.75765	2.49E-05
4.7522	0.00107	4.92315	5.56E-04	5.18269	9.56E-05	4.65799	5.01E-05	4.74518	2.78E-05	4.93097	2.05E-05
4.91923	0.00152	5.0962	4.98E-04	5.36485	1.32E-04	4.82171	5.81E-05	4.91197	3.67E-05	5.1043	1.32E-05
5.08627	0.00134	5.26924	6.73E-04	5.54702	1.45E-04	4.98544	6.75E-05	5.07876	2.92E-05	5.27762	1.61E-05
5.25331	0.00133	5.44229	7.03E-04	5.72919	9.99E-05	5.14916	6.04E-05	5.24555	2.40E-05	5.45094	2.24E-05
5.42034	0.00163	5.61533	5.85E-04	5.91136	1.84E-04	5.31289	5.65E-05	5.41234	3.92E-05	5.62426	2.52E-05
5.58738	0.0011	5.78838	6.23E-04	6.09353	9.22E-05	5.47661	4.68E-05	5.57913	3.94E-05	5.79758	1.36E-05
5.75442	0.00121	5.96142	6.44E-04	6.2757	1.51E-04	5.64034	5.73E-05	5.74592	3.11E-05	5.9709	2.48E-05
5.92145	0.0012	6.13447	5.78E-04	6.45786	1.26E-04	5.80406	7.53E-05	5.91271	2.76E-05	6.14422	2.27E-05
6.08849	0.00168	6.30752	5.20E-04	6.64003	9.60E-05	5.96779	7.27E-05	6.0795	3.00E-05	6.31754	2.00E-05
6.25553	0.00136	6.48056	4.93E-04	6.8222	1.38E-04	6.13151	6.03E-05	6.24629	3.64E-05	6.49086	2.05E-05
6.42256	0.00106	6.65361	6.10E-04	7.00437	1.68E-04	6.29524	4.81E-05	6.41308	2.84E-05	6.66418	1.73E-05

6.5896	0.00103	6.82665	5.32E-04	7.18654	9.39E-05	6.45896	6.07E-05	6.57987	3.26E-05	6.8375	1.94E-05
6.75664	0.00134	6.9997	5.90E-04	7.36871	1.54E-04	6.62269	6.10E-05	6.74666	3.68E-05	7.01082	1.33E-05
6.92367	0.00169	7.17274	5.29E-04	7.55087	1.17E-04	6.78642	5.69E-05	6.91345	3.37E-05	7.18414	1.65E-05
7.09071	0.0011	7.34579	5.58E-04	7.73304	1.43E-04	6.95014	6.68E-05	7.08024	2.07E-05	7.35746	2.41E-05
7.25775	9.42E-04	7.51884	4.75E-04	7.91521	1.34E-04	7.11387	5.70E-05	7.24703	3.16E-05	7.53079	2.28E-05
7.42479	0.00118	7.69188	4.41E-04	8.09738	1.39E-04	7.27759	6.92E-05	7.41382	2.97E-05	7.70411	1.59E-05
7.59182	0.00155	7.86493	5.76E-04	8.27955	1.14E-04	7.44132	5.72E-05	7.58061	2.66E-05	7.87743	1.92E-05
7.75886	0.00104	8.03797	6.22E-04	8.46172	1.08E-04	7.60504	5.86E-05	7.7474	3.63E-05	8.05075	2.18E-05
7.9259	0.00135	8.21102	3.92E-04	8.64388	1.80E-04	7.76877	6.04E-05	7.91419	4.17E-05	8.22407	2.29E-05
8.09293	0.0012	8.38406	4.63E-04	8.82605	1.37E-04	7.93249	5.57E-05	8.08098	3.49E-05	8.39739	2.19E-05
8.25997	0.00139	8.55711	8.14E-04	9.00822	1.16E-04	8.09622	6.06E-05	8.24777	3.08E-05	8.57071	1.82E-05
8.42701	0.00172	8.73016	6.18E-04	9.19039	9.63E-05	8.25994	7.92E-05	8.41456	2.66E-05	8.74403	1.46E-05
8.59404	0.00124	8.9032	6.60E-04	9.37256	1.17E-04	8.42367	5.36E-05	8.58135	4.32E-05	8.91735	1.53E-05
8.76108	9.17E-04	9.07625	5.37E-04	9.55472	1.15E-04	8.58739	7.26E-05	8.74814	2.03E-05	9.09067	2.13E-05
8.92812	9.27E-04	9.24929	6.12E-04	9.73689	1.12E-04	8.75112	7.78E-05	8.91493	3.48E-05	9.26399	2.12E-05
9.09515	0.00169	9.42234	5.72E-04	9.91906	9.52E-05	8.91485	6.14E-05	9.08172	3.53E-05	9.43731	2.37E-05
9.26219	0.00122	9.59538	6.57E-04	10.10123	1.27E-04	9.07857	6.13E-05	9.24851	2.72E-05	9.61063	2.70E-05
9.42923	0.0013	9.76843	4.47E-04	10.2834	1.37E-04	9.2423	6.09E-05	9.4153	3.30E-05	9.78395	1.74E-05
9.59626	0.00164	9.94148	6.26E-04	10.46557	1.36E-04	9.40602	5.34E-05	9.58209	3.86E-05	9.95728	2.17E-05
9.7633	0.00111	10.11452	6.23E-04	10.64773	1.29E-04	9.56975	6.49E-05	9.74888	3.42E-05	10.1306	2.17E-05
9.93034	0.00143	10.28757	5.85E-04	10.8299	1.79E-04	9.73347	4.82E-05	9.91567	2.63E-05	10.30392	2.17E-05
10.09737	0.00151	10.46061	6.44E-04	11.01207	1.40E-04	9.8972	6.08E-05	10.08246	2.63E-05	10.47724	3.30E-05
10.26441	0.00146	10.63366	5.34E-04	11.19424	1.22E-04	10.06092	6.88E-05	10.24925	3.39E-05	10.65056	2.21E-05
10.43145	0.0011	10.8067	6.65E-04	11.37641	1.09E-04	10.22465	6.87E-05	10.41604	3.87E-05	10.82388	2.53E-05
10.59848	0.00116	10.97975	6.63E-04	11.55858	1.22E-04	10.38837	6.30E-05	10.58283	2.47E-05	10.9972	1.66E-05
10.76552	0.00141	11.1528	5.64E-04	11.74074	1.25E-04	10.5521	6.97E-05	10.74962	2.63E-05	11.17052	2.69E-05
10.93256	0.00166	11.32584	6.35E-04	11.92291	1.08E-04	10.71582	5.34E-05	10.91641	3.74E-05	11.34384	2.08E-05
11.09959	0.0015	11.49889	5.65E-04	12.10508	1.31E-04	10.87955	5.55E-05	11.0832	3.20E-05	11.51716	2.28E-05
11.26663	0.00152	11.67193	7.55E-04	12.28725	9.14E-05	11.04327	5.45E-05	11.24999	2.83E-05	11.69048	1.66E-05
11.43367	0.00121	11.84498	7.19E-04	12.46942	1.37E-04	11.207	7.54E-05	11.41678	3.93E-05	11.8638	1.48E-05
11.6007	0.00122	12.01802	5.48E-04	12.65158	1.37E-04	11.37073	7.04E-05	11.58357	3.17E-05	12.03712	1.99E-05
11.76774	0.00177	12.19107	7.82E-04	12.83375	1.36E-04	11.53445	7.07E-05	11.75036	2.44E-05	12.21044	1.75E-05
11.93478	0.00139	12.36412	7.55E-04	13.01592	1.17E-04	11.69818	5.31E-05	11.91715	2.99E-05	12.38377	1.89E-05
12.10182	0.00144	12.53716	6.68E-04	13.19809	1.23E-04	11.8619	6.86E-05	12.08394	3.05E-05	12.55709	2.31E-05
12.26885	0.00134	12.71021	6.03E-04	13.38026	1.41E-04	12.02563	5.85E-05	12.25073	3.40E-05	12.73041	2.13E-05
12.43589	0.00118	12.88325	5.48E-04	13.56243	1.33E-04	12.18935	6.09E-05	12.41752	3.76E-05	12.90373	2.17E-05
12.60293	0.00112	13.0563	8.11E-04	13.74459	1.02E-04	12.35308	6.42E-05	12.58431	2.94E-05	13.07705	2.43E-05
12.76996	0.00128	13.22934	5.38E-04	13.92676	1.50E-04	12.5168	5.05E-05	12.75111	2.75E-05	13.25037	2.13E-05
12.937	0.00147	13.40239	5.14E-04	14.10893	1.72E-04	12.68053	6.52E-05	12.9179	3.41E-05	13.42369	1.84E-05
13.10404	8.41E-04	13.57544	7.12E-04	14.2911	1.44E-04	12.84425	7.75E-05	13.08469	3.27E-05	13.59701	2.39E-05
13.27107	0.0017	13.74848	6.58E-04	14.47327	1.65E-04	13.00798	6.75E-05	13.25148	3.74E-05	13.77033	2.30E-05
13.43811	0.0015	13.92153	7.79E-04	14.65544	1.39E-04	13.1717	7.49E-05	13.41827	3.33E-05	13.94365	2.72E-05

13.60515	0.00136	14.09457	7.99E-04	14.8376	1.25E-04	13.33543	6.19E-05	13.58506	4.39E-05	14.11697	2.33E-05
13.77218	0.00111	14.26762	5.47E-04			13.49915	5.91E-05	13.75185	3.48E-05	14.29029	2.76E-05
13.93922	0.00177	14.44066	7.57E-04			13.66288	7.60E-05	13.91864	3.00E-05	14.46361	2.22E-05
14.10626	0.00128	14.61371	6.05E-04			13.82661	7.22E-05	14.08543	1.87E-05	14.63693	1.53E-05
14.27329	0.00173	14.78676	6.65E-04			13.99033	5.98E-05	14.25222	2.85E-05	14.81026	2.60E-05
14.44033	0.00112	14.9598	3.69E-04			14.15406	6.89E-05	14.41901	3.05E-05	14.98358	2.28E-05
14.60737	0.00129					14.31778	8.69E-05	14.5858	2.49E-05		
14.7744	0.00138					14.48151	6.97E-05	14.75259	3.70E-05		
14.94144	0.00129					14.64523	7.76E-05	14.91938	3.18E-05		
						14.80896	5.04E-05				
						14.97268	6.55E-05				

Data/Fig. 3a:

Temperature (ºC)	MR (%)
20	4.73534
40	4.61586
60	4.36194
80	4.29694

Data/Fig. 3b:

Temperature (ºC)	MR (%)
20	0.4682
40	0.4716
60	0.4781
80	0.4625

Data/Fig. 4b:

Original		Recycled	
Magnetic field (mT)	MR (%)	Magnetic field (mT)	MR (%)
99.63779	0.02516	98.04259	0.03965
97.72162	0.03766	95.98103	0.05005
95.93271	0.04899	93.72946	0.06113
93.84365	0.05953	91.65881	0.07235
91.90554	0.07376	89.38354	0.08441
89.68732	0.09134	87.34524	0.09875
87.69482	0.10776	85.10837	0.1121
85.40294	0.12304	83.07322	0.12486
83.44266	0.13652	80.99234	0.13843
81.22059	0.158	78.75964	0.1527
79.23735	0.17886	76.79659	0.16758
77.14486	0.19582	74.65885	0.18182

75.10873	0.21863	72.6977	0.19928
72.94371	0.24386	70.67253	0.21654
71.01058	0.25929	68.61121	0.23567
68.82209	0.28103	66.69128	0.25383
66.83557	0.30775	64.56946	0.27425
64.86073	0.33378	62.55467	0.29656
62.865	0.35898	60.56086	0.32066
60.84542	0.38579	58.54924	0.34534
58.77576	0.41898	56.53084	0.37152
56.75761	0.45193	54.3748	0.39859
54.64183	0.48708	52.47745	0.42937
52.74655	0.52898	50.40611	0.46346
50.18671	0.56693	48.51658	0.49882
48.16822	0.61795	46.38427	0.5363

46.01384	0.66246	44.37986	0.57853
44.11351	0.71531	42.38564	0.62312
41.96096	0.77268	40.24351	0.67088
40.10329	0.8334	38.35546	0.72463
37.95798	0.89779	36.26233	0.78452
35.98892	0.96596	34.26057	0.85012
34.04146	1.04288	32.25155	0.92266
31.96699	1.13358	30.26145	1.0037
29.96087	1.22467	28.17826	1.0961
27.9643	1.32612	26.21255	1.1961
25.84728	1.43482	24.33453	1.30675
23.95374	1.55173	22.16714	1.42944
21.8306	1.68969	20.2452	1.56731
19.9123	1.84279	18.11431	1.72766
17.88285	2.01158	16.19585	1.90658
15.85273	2.20227	14.04987	2.10275
13.94536	2.4062	12.13829	2.31335
11.77875	2.63433	10.13998	2.54189
9.88603	2.88799	8.1534	2.7844
7.75153	3.14746	6.24558	3.03451
5.8329	3.42029	4.08266	3.29633
3.81561	3.68572	2.17443	3.5698
1.79585	3.94042	0.03968	3.86224
-0.20587	4.19415	-1.87317	4.23444
-2.24246	4.40423	-3.85834	4.49501
-4.24204	4.51284	-5.81091	4.58907
-6.22122	4.51214	-7.93229	4.4649
-8.23458	4.41764	-9.84577	4.14931
-10.1946	4.27214	-11.9467	3.7869
-12.2851	4.07892	-13.9117	3.42655
-14.2142	3.84323	-15.9154	3.08963
-16.2229	3.59341	-17.9155	2.75552
-18.3157	3.35586	-19.9064	2.44883
-20.1816	3.09925	-22.0062	2.15765
-22.2975	2.8494	-23.9673	1.91336
-24.1752	2.62545	-25.9204	1.69193
-26.3089	2.39576	-27.9363	1.49061
-28.2094	2.17246	-29.8983	1.31863
-30.3492	1.97646	-32.0142	1.17199
-32.2267	1.79429	-33.8867	1.04628
-34.3255	1.62736	-35.9642	0.93455
-36.195	1.47668	-37.8409	0.84021

-38.2849	1.34475	-39.9819	0.75551
-40.2526	1.22934	-41.9706	0.6844
-42.2825	1.12136	-43.9573	0.62538
-44.1846	1.0281	-45.945	0.57073
-46.3342	0.94495	-47.9417	0.51939
-48.2708	0.87152	-49.9244	0.47832
-50.2759	0.80607	-51.9211	0.43996
-52.262	0.74092	-53.9119	0.40474
-54.3816	0.68614	-55.8918	0.37208
-56.34	0.62787	-58.0103	0.34259
-58.2869	0.58079	-59.8917	0.31522
-60.3046	0.53411	-61.9007	0.28967
-62.3078	0.48723	-63.9031	0.26587
-64.2759	0.44679	-66.0263	0.24403
-66.2136	0.41204	-67.899	0.22444
-68.3387	0.37956	-70.049	0.20619
-70.2232	0.34604	-71.9387	0.18907
-72.3732	0.31396	-73.9333	0.17174
-74.2366	0.28757	-75.9623	0.15577
-76.3833	0.25636	-77.933	0.14303
-78.2914	0.23825	-79.9127	0.13073
-80.2869	0.21614	-81.9292	0.11749
-82.3642	0.1923	-84.012	0.10415
-84.3049	0.16137	-85.9716	0.09053
-86.3107	0.13833	-87.9724	0.07822
-88.3274	0.1203	-89.9779	0.06764
-90.32	0.10132	-91.9692	0.05521
-92.2721	0.09027	-93.7971	0.04629
-93.9066	0.07477	-94.6849	0.04317
-94.5655	0.06547	-94.6626	0.04434
-94.3301	0.06865	-93.8888	0.04914
-93.4871	0.07001	-92.5406	0.05531
-92.0949	0.08353	-90.8855	0.06454
-90.4134	0.09874	-89.0218	0.07486
-88.4867	0.10636	-86.7962	0.08669
-86.4029	0.12418	-84.6831	0.1003
-84.1335	0.14	-82.4063	0.11484
-82.055	0.15713	-80.1336	0.12941
-79.7295	0.17648	-77.8927	0.14519
-77.5693	0.1973	-75.6924	0.16213
-75.3801	0.217	-73.5103	0.17854
-73.2396	0.23671	-71.4061	0.19583

-71.077	0.2616	-69.1795	0.2149
-69.048	0.28603	-67.1989	0.23445
-66.8551	0.30792	-65.0039	0.25463
-64.7747	0.33838	-63.0664	0.27487
-62.7036	0.35957	-60.8879	0.29573
-60.6633	0.38758	-58.9191	0.31914
-58.5332	0.42691	-56.868	0.34527
-56.5149	0.45021	-54.8299	0.36751
-54.4889	0.46286	-52.8957	0.39527
-52.4639	0.4976	-50.7365	0.42701
-50.472	0.54261	-48.7809	0.46207
-48.3591	0.58569	-46.783	0.49639
-46.3614	0.63849	-44.6204	0.53423
-44.416	0.68631	-42.7188	0.57587
-42.2685	0.74279	-40.7297	0.6219
-40.3623	0.79913	-38.7285	0.67081
-38.2255	0.86199	-36.6128	0.72637
-36.303	0.93522	-34.614	0.78701
-34.1602	1.01072	-32.5911	0.85432
-32.2861	1.08514	-30.5995	0.92671
-30.1706	1.1689	-28.5371	1.00963
-28.2471	1.27193	-26.5745	1.10405
-26.0968	1.38725	-24.4401	1.20966
-24.2167	1.51267	-22.549	1.32301
-22.0793	1.64679	-20.4389	1.44846
-20.1059	1.8013	-18.4734	1.58761
-18.123	1.96713	-16.4506	1.7494
-16.0486	2.15641	-14.4099	1.93393
-14.0317	2.36814	-12.4	2.12996
-12.0366	2.5998	-10.4026	2.34399
-10.1228	2.84954	-8.41647	2.57472
-8.07033	3.11448	-6.31966	2.81872
-6.05801	3.38627	-4.43024	3.0742
-4.01917	3.64761	-2.30076	3.34352
-2.00198	3.90086	-0.39207	3.61754
0.0875	4.14246	1.74755	3.92471
1.96542	4.33948	3.6616	4.2889
4.10116	4.49934	5.8192	4.52966
6.00564	4.52507	7.7433	4.58324
8.11378	4.37587	9.70793	4.40177
10.08186	4.23588	11.86526	4.05702
12.07729	4.0056	13.78915	3.70314

14.20598	3.76364	15.79592	3.33461
16.09046	3.49187	17.78252	2.97954
18.20653	3.24591	19.67748	2.65684
20.07167	3.01497	21.84366	2.36179
22.17864	2.79034	23.76246	2.09689
24.13055	2.56142	25.91781	1.85606
26.13201	2.345	27.77789	1.63756
28.2408	2.13524	29.88912	1.44097
30.12402	1.95193	31.75393	1.27046
32.13938	1.78003	33.8421	1.12656
34.13679	1.61508	35.82484	1.00379
36.1406	1.47625	37.79365	0.90002
38.14814	1.35319	39.88644	0.80955
40.25239	1.23791	41.75348	0.73227
42.12934	1.15167	43.84446	0.66346
44.16281	1.06459	45.85334	0.60235
46.1781	0.98324	47.8491	0.55048
48.23901	0.91153	49.82949	0.50309
50.20927	0.8429	51.78216	0.46065
52.19196	0.78469	53.90366	0.42235
54.10378	0.72184	55.79549	0.38705
56.25667	0.66826	57.89665	0.35583
58.19183	0.61992	59.85572	0.32689
60.2915	0.57302	61.82945	0.30145
62.19602	0.52385	63.9413	0.27555
64.31325	0.48655	65.90162	0.25219
66.2985	0.44407	67.9037	0.23056
68.29892	0.40154	69.88904	0.21201
70.21051	0.36883	71.86381	0.1936
72.3279	0.33567	73.97803	0.17616
74.27223	0.30024	75.89049	0.15981
76.24898	0.27253	77.99406	0.14395
78.24111	0.24899	79.89444	0.1297
80.26643	0.22453	81.81311	0.11839
82.27085	0.20192	83.93712	0.10523
84.27859	0.1827	85.92041	0.09237
86.16627	0.15934	87.90942	0.0794
88.3182	0.13691	89.82372	0.06776
90.17707	0.118	91.94285	0.05525
92.31336	0.09874	93.93116	0.04365
94.19225	0.07201	95.94763	0.03264
96.30219	0.05857		

Data/Fig. 4c:

Original		Recycled	
Magnetic field (mT)	MR (%)	Magnetic field (mT)	MR (%)
32.66163	0.42815	26.07778	0.45002
32.58944	0.43085	26.02956	0.44891
32.40279	0.43039	25.97005	0.44946
32.15645	0.43379	25.90539	0.44942
31.83314	0.43676	25.78236	0.44981
31.45031	0.43877	25.52493	0.45082
31.05728	0.43617	25.15123	0.44872
30.67378	0.43825	24.60563	0.44765
30.22533	0.43519	23.9405	0.4485
29.82924	0.43419	23.26806	0.44733
29.37872	0.43322	22.54396	0.44652
28.98229	0.43421	21.76418	0.44515
28.54089	0.43399	20.71805	0.44378
28.14493	0.4325	19.84992	0.44201
27.70435	0.42492	18.9614	0.44118
27.29589	0.43106	17.7689	0.43797
26.89377	0.43616	16.78507	0.436
26.47793	0.43148	15.80786	0.43217
26.08686	0.4295	14.85062	0.42954
25.65566	0.43523	13.57692	0.4266
25.27694	0.43675	12.56129	0.42302
24.86592	0.4327	11.62343	0.41903
24.47491	0.43062	10.75764	0.41529
24.05703	0.43083	9.85154	0.41072
23.65704	0.42578	9.01133	0.40614
23.26217	0.42691	8.35262	0.40162
22.85441	0.42475	7.7406	0.39658
22.44626	0.42602	7.16046	0.39165
22.05415	0.42181	6.51299	0.38657
21.63648	0.42359	6.02459	0.38282
21.26384	0.43045	5.56258	0.37621
20.836	0.42418	5.11182	0.37024
20.46576	0.42367	4.60245	0.36585
20.02794	0.42385	4.19685	0.36097
19.63455	0.42422	3.81015	0.35605
19.23306	0.42085	3.43157	0.34987
18.84077	0.42348	3.07261	0.34559
18.41949	0.42749	2.61406	0.33753
18.02939	0.42431	2.25146	0.33251
17.62468	0.42166	1.9057	0.32379
17.20977	0.41852	1.4716	0.31631
16.81733	0.42276	1.1166	0.30957
16.41568	0.41699	0.76325	0.29844
16.03422	0.41655	0.39531	0.2835
15.59593	0.41675	-0.04093	0.26701
15.22173	0.41765	-0.38743	0.25214
14.79422	0.41159	-0.74242	0.23464
14.38701	0.41638	-1.10435	0.21458
14.00132	0.4258	-1.55588	0.18962
13.59062	0.41953	-1.91711	0.16381
13.19698	0.41838	-2.29465	0.13526
12.81804	0.42004	-2.68095	0.09547
12.39268	0.41504	-3.1505	0.05521
12.01206	0.41297	-3.52009	0.02954
11.60006	0.40764	-3.88857	0.00924
11.18743	0.40612	-4.30938	0.00125
10.78557	0.41225	-4.7069	0.03649
10.40105	0.40971	-5.07405	0.06685
9.96799	0.40835	-5.55119	0.11825
9.59137	0.40205	-5.92592	0.14493
9.16536	0.40333	-6.29751	0.18766
8.78959	0.40509	-6.67881	0.21496
8.38089	0.40528	-7.15993	0.23017
7.98785	0.40212	-7.53657	0.25038
7.56772	0.39824	-7.93352	0.27386
7.18435	0.39451	-8.30693	0.28238
6.77419	0.3992	-8.72408	0.2923
6.37566	0.39289	-9.14907	0.31106
5.95652	0.38985	-9.51995	0.32014
5.55967	0.38886	-9.9076	0.33387
5.14936	0.38836	-10.385	0.34365
4.76439	0.38448	-10.7649	0.34925
4.36634	0.38021	-11.2502	0.35717
3.94177	0.37685	-11.9726	0.36765
3.56598	0.36974	-12.628	0.37529
3.14141	0.36717	-13.3413	0.38179
2.76097	0.36381	-14.099	0.39245
2.34058	0.36183	-15.0968	0.3981
1.96057	0.35353	-15.9525	0.40391
1.54255	0.34493	-16.8243	0.40884
1.14844	0.34478	-17.9049	0.42667
0.74151	0.33236	-18.8183	0.43028

0.32372	0.31605	-19.7877	0.44169
-0.05105	0.30609	-20.7727	0.44414
-0.47224	0.29221	-21.9948	0.44682
-0.85279	0.2658	-22.9611	0.44989
-1.25545	0.24454	-23.9322	0.45319
-1.65373	0.20769	-24.8911	0.45455
-2.06421	0.16451	-26.1507	0.45671
-2.46485	0.10737	-27.1418	0.45763
-2.86919	0.05232	-28.1196	0.45919
-3.26009	1.55E-11	-29.0984	0.46014
-3.67246	0.05717	-30.3164	0.46134
-4.07699	0.07374	-31.2637	0.46279
-4.47969	0.11672	-32.1123	0.46275
-4.87738	0.13592	-32.6771	0.46287
-5.28168	0.17217	-33.0557	0.4633
-5.68533	0.19696	-33.1055	0.46301
-6.10379	0.21473	-32.9705	0.46278
-6.47711	0.22627	-32.6047	0.46243
-6.88456	0.25432	-32.131	0.46299
-7.27976	0.25844	-31.5122	0.46284
-7.68368	0.26651	-30.8281	0.46265
-8.09071	0.28282	-29.8229	0.46169
-8.5071	0.29439	-28.9533	0.46086
-8.88974	0.31237	-28.0504	0.46096
-9.29681	0.33147	-27.0791	0.45986
-9.69271	0.33073	-26.0898	0.4599
-10.0924	0.33685	-24.8405	0.45894
-10.5194	0.35013	-23.8123	0.45711
-10.8818	0.35825	-22.7869	0.45521
-11.3184	0.3619	-21.5718	0.45464
-11.692	0.36548	-20.4034	0.45253
-12.0735	0.36668	-19.3427	0.45082
-12.5037	0.37142	-18.0486	0.44775
-12.8898	0.37094	-17.0369	0.44525
-13.2928	0.37528	-15.9993	0.44219
-13.7231	0.39962	-14.9887	0.43936
-14.0972	0.40369	-13.8495	0.43508
-14.5203	0.40075	-13.0482	0.43222
-14.9128	0.40211	-12.3186	0.42812
-15.3124	0.40775	-11.4625	0.42574
-15.7256	0.40503	-10.8731	0.42075
-16.0952	0.40503	-10.3036	0.41824

-16.5182	0.40696	-9.64692	0.41517
-16.9036	0.40854	-9.18299	0.41278
-17.3257	0.4117	-8.71337	0.40857
-17.715	0.41007	-8.27983	0.40544
-18.1344	0.41908	-7.8141	0.40336
-18.5081	0.41679	-7.40057	0.39969
-18.9223	0.41892	-6.96254	0.39603
-19.3361	0.41787	-6.59372	0.39293
-19.729	0.41726	-6.2195	0.39017
-20.1228	0.42102	-5.86343	0.38678
-20.5217	0.42139	-5.50532	0.38193
-20.9421	0.42453	-5.06853	0.37781
-21.3195	0.42234	-4.72122	0.37292
-21.7423	0.42385	-4.37046	0.36807
-22.1406	0.42542	-3.94503	0.36426
-22.5333	0.42354	-3.59424	0.35948
-22.942	0.42646	-3.24021	0.35297
-23.3199	0.42472	-2.88257	0.34724
-23.7395	0.42685	-2.41664	0.34071
-24.1553	0.43054	-2.06675	0.3334
-24.5296	0.42921	-1.69577	0.32263
-24.9518	0.4346	-1.32115	0.31171
-25.3348	0.43422	-0.93337	0.29291
-25.7372	0.43373	-0.50945	0.27892
-26.1287	0.43196	-0.13016	0.2632
-26.5512	0.43279	0.22174	0.24872
-26.9527	0.43313	0.6921	0.23418
-27.354	0.42759	1.05723	0.21309
-27.7458	0.43049	1.42927	0.19974
-28.151	0.43829	1.85392	0.18048
-28.556	0.43126	2.28969	0.14462
-28.949	0.42932	2.67136	0.12148
-29.7374	0.43669	3.04428	0.07335
-30.1616	0.43397	3.4239	0.02835
-30.5419	0.43188	3.88324	0
-30.9649	0.42842	4.24467	0.00486
-31.2426	0.43224	4.63309	0.05941
-31.3467	0.43501	5.06912	0.12234
-31.2401	0.44005	5.49264	0.15707
-31.0384	0.43433	5.86753	0.18401
-30.7201	0.43192	6.28812	0.19779
-30.3582	0.43354	6.70841	0.20605

-29.9301	0.42799	7.09134	0.2222
-29.5118	0.42767	7.49794	0.2749
-29.0848	0.43057	7.99256	0.29963
-28.6249	0.42993	8.69058	0.32577
-28.1815	0.42885	9.31328	0.34209
-27.7484	0.42685	10.09115	0.35686
-27.3338	0.4281	10.91093	0.36766
-26.8747	0.43066	11.68816	0.38265
-26.4578	0.42795	12.70939	0.39217
-26.0355	0.4286	13.60383	0.40068
-25.6243	0.42407	14.53211	0.40823
-25.2115	0.42622	15.49726	0.41964
-24.815	0.42077	16.67227	0.42563
-24.4013	0.42791	17.62629	0.43136
-23.9991	0.42235	18.60455	0.43532
-23.5986	0.42345		
-23.1949	0.42168		
-22.7891	0.42655		
-22.3911	0.42617		
-21.9645	0.42345		
-21.5952	0.42409		
-21.1642	0.42338		
-20.7866	0.41954		
-20.3797	0.41621		
-19.9632	0.41725		
-19.5726	0.41433		
-19.1889	0.42203		
-18.7624	0.41569		
-18.3778	0.41936		
-17.9504	0.41499		
-17.5853	0.41098		
-17.1345	0.41284		
-16.7566	0.41602		
-16.3498	0.41345		
-15.9485	0.41014		
-15.5298	0.41762		
-15.1539	0.41231		
-14.7304	0.40428		
-14.332	0.40832		
-13.9284	0.40317		
-13.5261	0.4087		
-13.1325	0.40184		

-12.7142	0.40079		
-12.3173	0.39765		
-11.9208	0.40406		
-11.5383	0.40304		
-11.1043	0.39813		
-10.7251	0.39549		
-10.2989	0.39437		
-9.92576	0.39551		
-9.50954	0.39443		
-9.08275	0.40071		
-8.70594	0.38853		
-8.3394	0.38774		
-7.89313	0.39006		
-7.52354	0.38398		
-7.08786	0.38451		
-6.71083	0.38068		
-6.28526	0.38217		
-5.91914	0.37717		
-5.48209	0.37138		
-5.08895	0.364		
-4.68661	0.36363		
-4.28676	0.36189		
-3.88822	0.36403		
-3.46991	0.34962		
-3.09099	0.35052		
-2.67054	0.33856		
-2.26816	0.32843		
-1.87699	0.31678		
-1.47201	0.30611		
-1.07057	0.30087		
-0.66862	0.28251		
-0.28185	0.2685		
0.14026	0.23892		
0.53808	0.21002		
0.93879	0.18158		
1.36046	0.14327		
1.73145	0.07676		
2.16702	0.01923		
2.5404	0.01967		
2.96599	0.07038		
3.34274	0.10126		
3.77632	0.13074		

4.15333	0.17079		
4.55408	0.19226		
4.95552	0.21999		
5.36101	0.23512		
5.75389	0.25129		
6.16034	0.27786		
6.58318	0.28723		
6.96142	0.30533		
7.38673	0.32623		
7.75153	0.33013		
8.167	0.34603		
8.58685	0.35004		
8.95323	0.36177		
9.37944	0.35864		
9.76258	0.37259		
10.18269	0.36868		
10.56024	0.36893		
10.98382	0.38292		
11.38092	0.38221		
11.79502	0.38596		
12.19212	0.38665		
12.56389	0.39214		
12.98134	0.39356		
13.38423	0.39584		
13.78075	0.39752		
14.20342	0.40035		
14.5792	0.40092		
15.006	0.39807		
15.37974	0.40551		
15.80964	0.40049		
16.17552	0.40915		
16.59291	0.41242		
16.99832	0.41085		
17.39659	0.41283		

17.81316	0.41052		
18.19403	0.41086		
18.60292	0.41448		
18.98718	0.41405		
19.41896	0.41401		
19.8083	0.4159		
20.20475	0.41498		
20.62525	0.40776		
21.01038	0.41492		
21.41938	0.42221		
21.80472	0.413		
22.23396	0.42378		
22.62518	0.42208		
23.0193	0.42057		
23.41184	0.41434		
23.80924	0.42489		
24.21565	0.42444		
24.61663	0.42651		
25.0332	0.43165		
25.42476	0.42451		
25.82803	0.4263		
26.24225	0.42586		
26.61151	0.42944		
27.03835	0.4248		
27.42234	0.42915		
27.8234	0.42952		
28.23326	0.42943		
28.62766	0.43008		
29.0174	0.42998		
29.42892	0.42743		
29.83776	0.42688		
30.23079	0.42988		
30.63623	0.43128		

Data/Fig. 5c:

flat		15mm		20mm		30mm	
Magnetic field (mT)	MR (%)	Magnetic field (mT)	MR (%)	Magnetic field (mT)	MR (%)	Magnetic field (mT)	MR (%)
107.0406	0	107.2078	0.01611	106.1758	0.00365	106.4523	0
106.3317	0.00212	106.8618	0.01624	105.9094	0.00416	106.1166	0.00155
105.3299	0.00634	106.5688	0.01676	105.5446	0.00366	105.737	0.00307
103.8242	0.01276	106.1054	0.01738	105.0319	0.00437	105.2788	0.0056

102.1558	0.01927	105.3612	0.02004	104.272	0.00616	104.6783	0.00806
100.1052	0.02721	104.4471	0.02334	103.109	0.00806	103.8553	0.01112
98.11107	0.03468	102.9843	0.02774	101.9771	0.01173	103.0631	0.01405
96.01811	0.04319	101.6666	0.0326	100.3873	0.0157	102.2648	0.01737
93.74545	0.05199	100.2255	0.03836	98.91707	0.02063	101.229	0.02038
91.5724	0.06183	98.33746	0.04466	97.32957	0.02568	100.3407	0.02346
89.44805	0.07204	96.72002	0.05079	95.6653	0.03305	99.39268	0.02723
87.21664	0.08161	95.05211	0.05836	93.90017	0.03944	98.19015	0.03116
85.2078	0.09236	93.00997	0.06607	91.58704	0.04639	97.1977	0.03507
82.97878	0.10223	90.9307	0.07428	89.68698	0.05356	96.20374	0.03924
80.9805	0.11295	89.01477	0.08309	87.75363	0.06082	95.21041	0.04332
78.76776	0.12429	87.03658	0.09233	85.32066	0.06876	93.94002	0.04792
76.80995	0.13568	84.62599	0.0989	83.33641	0.07703	92.93647	0.05251
74.65468	0.14898	82.68887	0.10703	81.30853	0.08536	91.91369	0.05712
72.62845	0.16143	80.69034	0.11526	79.24316	0.09582	90.63434	0.06167
70.60751	0.17442	78.11229	0.12667	76.72604	0.10617	89.62155	0.06605
68.63453	0.18909	76.05074	0.13925	74.72999	0.11827	88.59516	0.07094
66.67062	0.20432	73.97938	0.15183	72.69436	0.12997	87.34258	0.07563
64.51854	0.22073	71.90099	0.16446	70.16348	0.14277	86.36119	0.08067
62.61898	0.23829	69.34551	0.17747	68.18575	0.15565	85.34403	0.08602
60.45803	0.25629	67.34259	0.19192	66.18954	0.16997	84.27745	0.09094
58.47822	0.27592	65.30295	0.20676	64.15929	0.18587	83.12486	0.09598
56.4581	0.29547	63.27532	0.22233	61.66483	0.20228	82.08268	0.10138
54.41898	0.31876	61.31428	0.24071	59.64768	0.2195	80.81706	0.10682
52.36998	0.34258	58.85641	0.25911	57.66391	0.23852	79.83972	0.11249
50.27091	0.36946	56.91253	0.27881	55.45804	0.25945	78.82119	0.11773
48.39155	0.39625	54.9156	0.30122	53.25604	0.28062	77.6144	0.12352
46.27637	0.42402	52.47246	0.32545	51.26367	0.30417	76.64168	0.1292
44.39019	0.45621	50.55084	0.35116	49.24557	0.33005	75.7254	0.13542
42.25665	0.49172	48.60256	0.37764	46.80665	0.35739	74.5529	0.14142
40.36857	0.53045	46.19873	0.40654	44.91353	0.38891	73.60136	0.14765
38.22741	0.57211	44.26773	0.43788	43.00883	0.42106	72.64298	0.15418
36.21436	0.61935	42.32058	0.47442	41.03153	0.45677	71.58706	0.16046
34.24182	0.67077	39.91952	0.51416	38.77805	0.49535	70.5553	0.1666
32.16234	0.73033	38.00636	0.5563	36.63884	0.53715	69.613	0.17329
30.17432	0.79393	36.07126	0.6015	34.72597	0.58438	68.66686	0.18074
28.19044	0.86424	34.15138	0.65312	32.82945	0.63857	67.69555	0.18814
26.27375	0.94356	31.74262	0.70786	30.52347	0.69518	66.55084	0.19545
24.12146	1.03336	29.83115	0.77001	28.60776	0.75984	65.6084	0.2029
22.12241	1.13475	27.91852	0.83772	26.6781	0.83306	64.67504	0.21039
20.13563	1.25247	25.96315	0.91599	24.74991	0.91463	63.49688	0.21897
18.12927	1.387	23.55958	1.00681	22.3309	1.00788	62.55407	0.22786

16.12216	1.54221	21.66082	1.10952	20.40501	1.11594	61.60382	0.23648
14.21295	1.71952	19.76695	1.23183	18.52373	1.24108	60.44427	0.24551
12.09926	1.92008	17.82568	1.36681	16.4038	1.38362	59.53603	0.25483
10.11827	2.1465	15.91077	1.52298	14.28824	1.54536	58.5833	0.26473
8.15734	2.38352	13.53957	1.703	12.40506	1.72833	57.44975	0.27427
6.1209	2.63291	11.65946	1.90415	10.51532	1.93272	56.51423	0.28503
4.12162	2.89026	9.77502	2.12665	8.40117	2.14977	55.59195	0.29572
2.1312	3.16066	7.85659	2.35399	6.25654	2.37878	54.37464	0.30695
0.05009	3.46156	5.46977	2.60136	4.33158	2.62074	53.44496	0.31839
-1.89381	3.87162	3.55612	2.857	2.4441	2.86817	52.51524	0.33071
-3.96554	4.13671	1.66665	3.11987	0.08029	3.13343	51.45371	0.3433
-5.83524	4.22009	-0.22373	3.44104	-1.83144	3.49516	50.38726	0.35604
-7.94152	4.09505	-2.58527	3.79075	-3.7092	3.76437	49.42974	0.36926
-9.81886	3.79563	-4.47945	3.99483	-5.59107	3.89569	48.37481	0.38307
-11.971	3.42034	-6.39032	4.01683	-7.56234	3.83488	47.3314	0.3978
-13.8989	3.04058	-8.27382	3.8427	-9.96824	3.61151	46.38748	0.41243
-15.9189	2.67411	-10.683	3.52614	-11.7859	3.291	45.41839	0.42781
-17.9415	2.34232	-12.5374	3.1689	-13.8507	2.94636	44.21899	0.4446
-19.9198	2.05121	-14.4536	2.80517	-15.9244	2.60322	43.26365	0.46171
-21.883	1.79139	-16.3657	2.4703	-17.8101	2.29626	42.3167	0.47903
-23.9581	1.55972	-18.5103	2.16325	-19.6804	2.01125	41.37756	0.49828
-25.8478	1.35918	-20.6067	1.89438	-22.0714	1.76317	40.22861	0.51805
-27.9904	1.19138	-22.5285	1.6555	-23.9187	1.53867	39.29463	0.53909
-29.8528	1.04808	-24.4086	1.44124	-25.8068	1.34607	38.35152	0.56061
-31.9811	0.92645	-26.3033	1.25712	-27.7425	1.16826	37.29367	0.58364
-33.9328	0.8234	-28.6248	1.09561	-30.1471	1.02171	36.21401	0.60761
-35.9096	0.7374	-30.4913	0.96105	-32.1026	0.89771	35.25269	0.63308
-38.0092	0.66735	-32.3598	0.84584	-33.9406	0.79479	34.2965	0.65965
-39.9921	0.60602	-34.5166	0.75039	-35.8462	0.70707	33.22761	0.68735
-41.971	0.55275	-36.6018	0.67087	-37.7694	0.63024	32.14555	0.71656
-43.9601	0.50559	-38.4898	0.60489	-40.1875	0.56734	31.18477	0.74759
-45.9292	0.46851	-40.3771	0.54778	-42.0991	0.51425	30.24413	0.7805
-47.9416	0.43408	-42.5378	0.4993	-43.947	0.46798	29.0815	0.8153
-49.9131	0.40092	-44.6241	0.45579	-45.7681	0.4266	28.15498	0.85091
-51.8674	0.36936	-46.4781	0.41935	-48.1264	0.39102	27.11577	0.88901
-53.8519	0.34386	-48.3749	0.38477	-49.9487	0.35834	26.07536	0.93128
-56.0145	0.32224	-50.7006	0.35447	-51.7724	0.32836	25.12686	0.97499
-57.873	0.29948	-52.5497	0.32574	-53.6798	0.30175	24.16015	1.02143
-59.9916	0.27869	-54.4547	0.30068	-56.0827	0.27832	23.21117	1.07139
-61.9521	0.26003	-56.3422	0.27693	-57.9613	0.25624	22.05027	1.12461
-63.9517	0.24202	-58.7359	0.25525	-59.889	0.23548	21.12158	1.18012
-65.9573	0.22575	-60.6071	0.23468	-61.9438	0.217	20.07824	1.2415

-67.938	0.21023	-62.4567	0.21558	-64.0617	0.19895	19.02927	1.30572
-70.021	0.194	-64.743	0.1975	-65.9121	0.18235	18.10002	1.37482
-71.9574	0.18049	-66.5976	0.18128	-67.8021	0.16749	17.04223	1.45038
-73.929	0.16894	-68.4919	0.16719	-70.1589	0.15147	16.02438	1.52973
-75.9407	0.15682	-70.4156	0.1513	-72.0104	0.13794	15.11628	1.61487
-77.9549	0.14507	-72.5332	0.13771	-73.8613	0.12588	13.93795	1.71055
-79.8435	0.13246	-74.6183	0.12581	-75.7433	0.11331	12.91018	1.81732
-82.0157	0.12134	-76.4812	0.11419	-78.1661	0.10169	11.85765	1.93395
-83.9714	0.11126	-78.7381	0.10331	-80.0579	0.09047	10.7917	2.06017
-86.0661	0.10193	-80.621	0.0936	-81.9712	0.07961	9.58688	2.19305
-87.9755	0.09296	-82.5146	0.08233	-83.8962	0.06844	8.38742	2.3303
-90.0628	0.08616	-84.4187	0.07219	-86.0532	0.0583	7.33901	2.46032
-91.9085	0.07815	-86.4447	0.06211	-88.1653	0.04987	6.31679	2.59918
-93.7165	0.06999	-88.6163	0.05269	-90.0154	0.04136	5.33392	2.7338
-94.4466	0.06665	-90.4308	0.04538	-91.6527	0.03454	4.10655	2.86977
-94.3734	0.0654	-91.7307	0.04045	-93.1183	0.03056	3.1207	3.00766
-93.5531	0.06985	-92.649	0.03728	-93.7664	0.028	2.16348	3.14479
-92.2659	0.07464	-93.0269	0.03651	-94.013	0.02775	1.11237	3.28263
-90.6334	0.08284	-92.9451	0.03712	-93.8383	0.02986	0.09669	3.426
-88.7364	0.09104	-92.5182	0.03845	-93.2987	0.03316	-0.80486	3.58495
-86.71	0.0988	-91.6786	0.04253	-92.5238	0.03539	-1.68409	3.79009
-84.5641	0.10982	-90.6774	0.04683	-91.5006	0.03982	-2.66972	3.98376
-82.3733	0.12058	-89.5011	0.05306	-90.053	0.04403	-3.61557	4.11785
-80.054	0.13064	-87.775	0.05886	-88.4431	0.05036	-4.41266	4.22975
-78.0072	0.14141	-86.2127	0.06508	-86.9016	0.05707	-5.47303	4.29857
-75.7007	0.15422	-84.5791	0.07386	-85.1246	0.0644	-6.30651	4.31847
-73.6145	0.16555	-82.7666	0.08263	-83.2886	0.07353	-7.15767	4.29763
-71.36	0.17751	-80.3569	0.09157	-81.3013	0.08336	-8.11981	4.23089
-69.3374	0.19166	-78.3933	0.10198	-79.0436	0.09371	-9.10148	4.12284
-67.1884	0.20685	-76.3171	0.11278	-76.9712	0.10418	-9.94062	3.98202
-65.1258	0.22134	-74.3127	0.12353	-74.5171	0.11671	-10.9195	3.81668
-62.9561	0.23823	-71.7466	0.13644	-72.4253	0.13013	-11.8885	3.63929
-61.0319	0.25455	-69.6503	0.14953	-70.3123	0.14392	-12.7686	3.44834
-58.8472	0.27198	-67.5064	0.16425	-67.8955	0.15843	-13.6814	3.25813
-56.9365	0.2902	-65.3412	0.1795	-65.5528	0.17536	-14.5853	3.06954
-54.7795	0.31154	-63.2058	0.19547	-63.3784	0.19192	-15.7608	2.8836
-52.8391	0.33404	-61.0177	0.21367	-61.2513	0.21061	-16.6818	2.70404
-50.7092	0.35792	-58.5371	0.2329	-58.9563	0.22955	-17.6045	2.53904
-48.7113	0.38214	-56.1613	0.25328	-56.6487	0.25013	-18.6456	2.3754
-46.6945	0.40974	-54.0546	0.27357	-54.5928	0.2733	-19.6816	2.22019
-44.7039	0.43972	-51.9523	0.30197	-52.5608	0.29715	-20.6204	2.07472
-42.7313	0.4736	-49.8981	0.32854	-49.9898	0.32323	-21.5851	1.94127

-40.5522	0.51118	-47.3776	0.35698	-47.9917	0.3522	-22.6296	1.81502
-38.6585	0.54952	-45.3725	0.38761	-45.9863	0.38262	-23.6643	1.69452
-36.5434	0.5932	-43.3616	0.42233	-43.6573	0.41681	-24.583	1.57965
-34.6314	0.64172	-41.2513	0.4606	-41.3342	0.45317	-25.7176	1.47466
-32.5127	0.69443	-38.7471	0.49967	-39.3351	0.495	-26.6373	1.37536
-30.5941	0.75417	-36.7527	0.54472	-37.3022	0.53828	-27.5774	1.28691
-28.4822	0.82013	-34.7569	0.59469	-35.3644	0.58791	-28.5458	1.20366
-26.609	0.89435	-32.5394	0.65043	-32.9635	0.64215	-29.6174	1.12509
-24.4359	0.97863	-30.3528	0.71092	-30.9782	0.70278	-30.6831	1.05326
-22.4311	1.07321	-28.4035	0.77975	-28.9638	0.76976	-31.619	0.98893
-20.4502	1.18394	-26.459	0.8565	-27.0308	0.84463	-32.564	0.92932
-18.4207	1.30751	-24.4736	0.94312	-24.5853	0.92884	-33.7337	0.87426
-16.3998	1.45154	-22.0391	1.04309	-22.6472	1.02323	-34.6721	0.82414
-14.2907	1.61718	-20.1088	1.15618	-20.6854	1.13443	-35.6257	0.77816
-12.324	1.80366	-18.2081	1.28533	-18.7221	1.25976	-36.5659	0.73543
-10.436	2.01394	-16.2946	1.43438	-16.8049	1.40356	-37.7594	0.69578
-8.31532	2.24563	-13.8964	1.60625	-14.4397	1.56941	-38.6958	0.65857
-6.33456	2.48955	-12.0081	1.80528	-12.5868	1.75422	-39.6116	0.62671
-4.30254	2.74167	-10.1596	2.01951	-10.6634	1.96921	-40.7882	0.5951
-2.29497	3.00636	-7.83527	2.25003	-8.31211	2.19051	-41.7486	0.56683
-0.31454	3.28147	-5.99194	2.4902	-6.50101	2.42363	-42.6723	0.53988
1.68379	3.62346	-4.10325	2.74325	-4.21802	2.66296	-43.7037	0.51545
3.68128	3.98559	-1.70881	3.00486	-2.3408	2.91243	-44.748	0.49188
5.83524	4.15971	0.17309	3.3051	-0.46698	3.19444	-45.6767	0.47078
7.73652	4.12551	2.0925	3.66751	1.90987	3.53037	-46.601	0.45131
9.74471	3.91254	3.99088	3.88364	3.74456	3.7183	-47.7758	0.43116
11.73125	3.57465	6.39032	3.91018	5.59107	3.74737	-48.6917	0.4123
13.78643	3.19571	8.37645	3.77301	7.74059	3.61647	-49.8217	0.39545
15.84121	2.82129	10.36854	3.49343	9.83731	3.37331	-50.7549	0.37851
17.83183	2.47761	12.31553	3.1411	11.7381	3.05475	-51.7738	0.36363
19.8296	2.17178	14.20783	2.78302	13.63717	2.72504	-52.7958	0.34993
21.75817	1.8976	16.52174	2.44442	15.98981	2.40785	-53.7413	0.33662
23.89761	1.65545	18.41735	2.13796	17.84054	2.11981	-54.6887	0.32214
25.91295	1.44501	20.30378	1.86957	19.712	1.86077	-55.843	0.30926
27.88086	1.26105	22.44846	1.62865	22.06727	1.62625	-56.7602	0.29684
29.8068	1.10629	24.54855	1.41361	23.95981	1.41824	-57.6881	0.28543
31.94116	0.97871	26.42291	1.22738	25.85624	1.23576	-58.8565	0.2742
33.92294	0.86686	28.30423	1.06333	27.98952	1.0749	-59.8054	0.26296
35.93387	0.77169	30.62434	0.92725	30.08348	0.9432	-60.7614	0.25189
37.84266	0.69351	32.51527	0.81052	31.96822	0.82915	-61.6738	0.24182
39.97883	0.62828	34.41525	0.71441	33.87166	0.72759	-62.8589	0.23257
41.85391	0.57313	36.32372	0.63455	36.20654	0.64636	-63.7854	0.2221

43.95901	0.52456	38.66401	0.56527	38.0787	0.57702	-64.7332	0.2131
46.03695	0.48165	40.55714	0.50783	39.90068	0.51378	-65.9349	0.20385
47.9444	0.44385	42.46393	0.45742	42.20226	0.46198	-66.8422	0.19498
49.93733	0.41	44.34374	0.41414	44.08336	0.41693	-67.8148	0.1862
51.92913	0.3804	46.71502	0.37657	45.96456	0.37799	-68.7352	0.17857
54.04084	0.3531	48.59188	0.34155	48.12833	0.34338	-69.9003	0.17094
55.91132	0.3282	50.50786	0.31	50.26334	0.31145	-70.8463	0.16307
58.04245	0.30434	52.61897	0.28092	52.16458	0.28308	-71.8085	0.15541
59.91087	0.2834	54.73593	0.25671	54.08389	0.25656	-72.8226	0.14764
61.99428	0.26396	56.61945	0.23101	56.2023	0.23034	-74.0578	0.14109
63.88356	0.24721	58.52546	0.20905	58.34696	0.20905	-75.0766	0.13407
65.99478	0.23041	60.85168	0.19036	60.25934	0.18946	-76.0575	0.12725
67.88182	0.21283	62.6865	0.17374	62.11751	0.16885	-77.0121	0.12052
70.03434	0.19799	64.57238	0.15752	64.21696	0.15051	-78.2127	0.11457
71.90336	0.18364	66.89994	0.14152	66.42289	0.1341	-79.1515	0.10865
74.02094	0.16991	68.75061	0.12652	68.30672	0.11902	-80.1313	0.10264
75.88946	0.15755	70.65692	0.11352	70.19943	0.10572	-81.0503	0.0976
77.98961	0.1462	72.56689	0.10167	72.13106	0.09127	-82.16	0.09206
79.94878	0.13414	74.69582	0.0885	74.28839	0.07907	-83.1082	0.08672
81.95526	0.12423	76.8131	0.07526	76.43955	0.06659	-84.0528	0.08183
84.06387	0.11322	78.72103	0.06547	78.36899	0.05513	-85.0917	0.07709
86.04824	0.10485	80.59722	0.05552	80.28121	0.04706	-86.1494	0.07206
88.01196	0.09306	82.70984	0.04662	82.35138	0.03703	-87.1319	0.06757
90.10639	0.0836	84.87835	0.03779	84.40019	0.02852	-88.0553	0.06253
91.99841	0.07418	86.78739	0.02822	86.23958	0.01837	-89.0556	0.05776
94.01285	0.06736	88.63897	0.01783	88.12714	0.00942	-90.3484	0.05201
96.04149	0.05928	90.60021	0.00863	90.48325	0	-91.4659	0.04632
98.02361	0.05262	92.9754	0			-92.5239	0.04196
						-93.708	0.03721
						-94.6763	0.03404
						-95.2336	0.03228
						-95.6301	0.03149
						-95.7569	0.03187
						-95.6314	0.03273
						-95.3471	0.03391
						-94.9511	0.03637
						-94.2023	0.03925
						-93.5533	0.04228
						-92.8149	0.04584
						-91.977	0.04983
						-90.8855	0.05408
						-89.9424	0.05807

						-89.0489	0.0625
						-87.7957	0.06789
						-86.744	0.07277
						-85.596	0.07836
						-84.4478	0.08419
						-83.3963	0.08995
						-82.3476	0.09533
						-81.0033	0.10156
						-79.9347	0.10768
						-78.8715	0.11327
						-77.8269	0.11961
						-76.5343	0.12575
						-75.5314	0.13201
						-74.5046	0.13822
						-73.2434	0.14528
						-72.2215	0.1521
						-71.2321	0.15878
						-70.1299	0.16574
						-69.0844	0.17279
						-67.9278	0.18056
						-66.95	0.18836
						-66.0051	0.19612
						-64.7316	0.20434
						-63.751	0.21306
						-62.7974	0.22196
						-61.5839	0.23107
						-60.641	0.23995
						-59.7221	0.24908
						-58.5281	0.25936
						-57.5971	0.26946
						-56.6371	0.28012
						-55.477	0.29158
						-54.538	0.30283
						-53.5995	0.31456
						-52.5082	0.32719
						-51.4252	0.34009
						-50.4751	0.35258
						-49.5265	0.36629
						-48.3368	0.38093
						-47.3783	0.39567
						-46.4396	0.41119
						-45.2514	0.42726

						-44.3285	0.444
						-43.369	0.46149
						-42.2803	0.4788
						-41.2077	0.49842
						-40.2368	0.5191
						-39.2927	0.54061
						-38.2399	0.56309
						-37.1517	0.58694
						-36.2125	0.61143
						-35.259	0.63692
						-34.0755	0.66365
						-33.147	0.693
						-32.2315	0.72299
						-31.0438	0.75503
						-30.1089	0.78915
						-29.0769	0.8242
						-28.0211	0.86214
						-27.061	0.9023
						-26.0962	0.94424
						-24.9337	0.98976
						-24.0056	1.03663
						-23.0385	1.08785
						-22.0902	1.1436
						-20.8894	1.20174
						-19.9699	1.26241
						-19.0308	1.32775
						-17.8467	1.401
						-16.8543	1.47822
						-15.9086	1.55934
						-14.9896	1.64698
						-13.8207	1.74153
						-12.8842	1.84162
						-11.9382	1.9474
						-10.8912	2.06181
						-9.78307	2.18053
						-8.8034	2.30182
						-7.84121	2.42386
						-6.89236	2.55191
						-5.80895	2.67961
						-4.73943	2.80759
						-3.78196	2.94219
						-2.83118	3.07643

						-1.64737	3.21818
						-0.70719	3.35962
						0.21807	3.52022
						1.17212	3.71741
						2.35189	3.92997
						3.27506	4.08885
						4.21683	4.1992
						5.37512	4.26028
						6.30651	4.26615
						7.2363	4.22692
						8.41015	4.13721
						9.35236	4.00459
						10.29675	3.83674
						11.24775	3.65103
						12.347	3.45955
						13.40356	3.26003
						14.33861	3.06531
						15.48506	2.8714
						16.42305	2.6883
						17.36279	2.51761
						18.29435	2.35727
						19.4693	2.20178
						20.41108	2.0601
						21.35182	1.92266
						22.54337	1.79837
						23.50589	1.67759
						24.46507	1.5663
						25.49477	1.45995
						26.53475	1.36126
						27.47196	1.26949
						28.52356	1.19002
						29.57436	1.11269
						30.52577	1.04206
						31.47802	0.97686
						32.55185	0.91753
						33.62297	0.8623
						34.55301	0.81183
						35.49195	0.76721
						36.6764	0.72637
						37.6024	0.68907
						38.56291	0.65522
						39.52083	0.6228

						40.69434	0.59277
						41.61642	0.56453
						42.53671	0.53732
						43.69805	0.51382
						44.63333	0.49034
						45.5932	0.46846
						46.54512	0.44833
						47.73214	0.42989
						48.67551	0.41215
						49.61005	0.39501
						50.54573	0.37898
						51.63462	0.36403
						52.71855	0.35014
						53.6622	0.33632
						54.61677	0.32341
						55.56488	0.31109
						56.74409	0.29942
						57.71601	0.28793
						58.62343	0.27691
						59.69068	0.26625
						60.75268	0.25559
						61.71327	0.24597
						62.67657	0.23559
						63.6233	0.22659
						64.78694	0.21797
						65.70553	0.20855
						66.64687	0.19927
						67.83654	0.19104
						68.77968	0.18303
						69.69303	0.1756
						70.84931	0.16874
						71.75806	0.1614
						72.71333	0.15417
						73.68228	0.14751
						74.91469	0.14102
						75.87741	0.13484
						76.85773	0.12833
						77.77993	0.12264
						78.93056	0.11685
						79.84665	0.11133
						80.98923	0.1055
						81.93682	0.10002

						82.88124	0.09447
						83.79923	0.08944
						84.9619	0.08429
						85.86759	0.07924
						86.7876	0.07453
						87.96266	0.06952
						88.90478	0.06481
						89.84692	0.06115
						90.88601	0.0563
						92.00379	0.05216
						92.92617	0.04784
						93.84404	0.04385
						94.80118	0.03946
						96.00763	0.03587
						96.91053	0.03164

Data/Fig. 5d:

Radius (mm)	MR (%)
35	4.22
30	4.32
20	3.9
15	4.02

Data/Fig. 5e:

Degree (˚)	Resistance (Ω)
0	44.60143
7.57952	44.6855
11.45916	44.79382
14.88588	44.92724
18.11852	45.05025
21.24584	45.19491
24.30854	45.35537
27.32835	45.51261
30.31808	45.67821
33.28584	45.78785
36.23703	45.9036

Data/Fig. 5f:

Time (s)	MR (%)
0	0.05031
0.20047	0.0493
0.40193	0.05208

0.6014	0.05275
0.80186	0.05079
1.00233	0.05228
1.20279	0.05164

1.40226	0.05061
1.60273	0.04908
1.80219	0.05135
2.00365	0.04903

2.20518	0.05098
2.40558	0.04977
2.6061	0.04904
2.80663	0.04709

3.00698	0.0454
3.20644	0.04683
3.40691	0.04719
3.60637	0.05305

3.80684	0.04294
4.00631	0.04891
4.20777	0.04566
4.40823	0.0468
4.60876	0.04641
4.80919	0.04977
5.00863	0.04575
5.21009	0.04867
5.41056	0.04337
5.61202	0.04878
5.81154	0.04638
6.01295	0.04461
6.21242	0.04583
6.41388	0.0493
6.61336	0.04637
6.81384	0.04662
7.01428	0.04624
7.21575	0.04687
7.4162	0.04869
7.61668	0.04548
7.81713	0.04801
8.0166	0.04445
8.21806	0.04869
8.41753	0.0478
8.618	0.0443
8.81846	0.04389
9.01998	0.0442
9.22041	0.04122
9.42088	0.04236
9.62131	0.04127
9.82277	0.04022
10.02224	0.04709
10.22271	0.04504
10.42318	0.04574
10.62267	0.0415
10.82312	0.04441
11.02257	0.04177
11.22304	0.04748
11.42257	0.04437
11.62297	0.04427
11.82343	0.04666
12.0249	0.04558

12.22439	0.04737
12.42489	0.04757
12.62429	0.04987
12.82478	0.0474
13.02429	0.04883
13.22575	0.04555
13.42622	0.04825
13.62563	0.04896
13.82708	0.04689
14.02755	0.04609
14.22805	0.047
14.42751	0.05006
14.62894	0.04918
14.82941	0.04993
15.02987	0.04519
15.22934	0.04574
15.43087	0.04653
15.63127	0.05073
15.83276	0.04833
16.03419	0.05002
16.23366	0.04765
16.43515	0.05175
16.63559	0.04832
16.83605	0.04665
17.03651	0.04887
17.23798	0.04487
17.43844	0.04891
17.6399	0.04823
17.84037	0.0476
18.04085	0.0486
18.2413	0.05104
18.44177	0.04727
18.64229	0.04975
18.84275	0.04657
19.04416	0.04555
19.24469	0.04712
19.44508	0.04789
19.64557	0.04898
19.84601	0.05008
20.04649	0.04908
20.24794	0.05119
20.44843	0.04904

20.64987	0.05222
20.84933	0.04899
21.05082	0.05019
21.25026	0.05013
21.45073	0.04748
21.65026	0.04905
21.85066	0.04902
22.05112	0.0477
22.25159	0.04942
22.45205	0.04761
22.65252	0.0478
22.85398	0.04755
23.05444	0.04764
23.25591	0.04842
23.45538	0.04585
23.65684	0.04882
23.8573	0.04971
24.05877	0.05008
24.25929	0.04947
24.45972	0.04926
24.66016	0.04679
24.85963	0.04805
25.06109	0.04654
25.26055	0.04894
25.46202	0.04704
25.66148	0.05014
25.86195	0.05044
26.06241	0.04419
26.26288	0.04387
26.46334	0.0444
26.66381	0.04753
26.86427	0.04381
27.06374	0.04485
27.2652	0.0469
27.46567	0.0447
27.66713	0.04658
27.86659	0.0468
28.06806	0.044
28.26852	0.04878
28.46899	0.04283
28.66852	0.04416
28.86992	0.0437

29.07038	0.04618
29.27185	0.04545
29.47231	0.04606
29.67277	0.0436
29.87423	0.04737
30.07372	0.04409
30.27417	0.04537
30.47463	0.0435
30.67609	0.04276
30.87556	0.04717
31.07602	0.0439
31.27649	0.04824
31.47696	0.04379
31.67642	0.04077
31.87788	0.0417
32.07835	0.04092
32.27882	0.04074
32.48028	0.04177
32.68074	0.03977
32.88121	0.04176
33.08167	0.04201
33.28213	0.04018
33.4816	0.03914
33.68207	0.04315
33.88253	0.04394
34.083	0.03745
34.28346	0.04164
34.48493	0.04138
34.68539	0.03823
34.88685	0.04116
35.08632	0.04275
35.28778	0.04148
35.48924	0.03932
35.68971	0.03716
35.89117	0.04502
36.09163	0.04199
36.2921	0.03854
36.49156	0.03907
36.69303	0.03955
36.89353	0.03813
37.09396	0.03773
37.29348	0.043

37.49489	0.03973
37.69535	0.04181
37.89581	0.03758
38.09728	0.04002
38.29689	0.03857
38.49823	0.03964
38.69867	0.0393
38.89914	0.03798
39.09965	0.0417
39.30009	0.04021
39.50056	0.03964
39.70199	0.04014
39.90146	0.03789
40.10192	0.03511
40.30239	0.03735
40.50285	0.03604
40.70233	0.0401
40.90279	0.03834
41.10325	0.04125
41.30372	0.03819
41.50418	0.04138
41.70465	0.03654
41.90611	0.04026
42.10557	0.04131
42.30609	0.04063
42.50551	0.04134
42.70597	0.03871
42.90644	0.03991
43.10691	0.04082
43.30737	0.04269
43.50789	0.04371
43.7083	0.03742
43.90876	0.03951
44.10922	0.04535
44.30969	0.04208
44.51121	0.0422
44.71062	0.04636
44.91108	0.04651
45.11155	0.04635
45.31301	0.04645
45.51248	0.04782
45.71301	0.05012

45.91245	0.04969
46.11287	0.05958
46.31334	0.0576
46.5138	0.0771
46.71527	0.32043
46.91473	0.69789
47.1152	0.97894
47.31467	1.18835
47.51613	1.21266
47.71659	1.22032
47.91706	1.22714
48.11655	1.22225
48.31699	1.22962
48.51646	1.23082
48.71792	1.23065
48.91741	1.22838
49.11785	1.23142
49.31931	1.23414
49.51981	1.23155
49.72124	1.23054
49.92178	1.23041
50.12319	1.2375
50.32366	1.23344
50.52509	1.23566
50.72456	1.23416
50.92502	1.23235
51.12449	1.24039
51.32595	1.23292
51.5265	1.23649
51.72794	1.23477
51.92738	1.23499
52.12782	1.23499
52.32939	1.23989
52.52974	1.23677
52.7312	1.24217
52.93167	1.23938
53.1332	1.23968
53.33261	1.23953
53.53309	1.24269
53.73353	1.23913
53.93403	1.24385
54.13447	1.23913

54.33598	1.23796
54.53538	1.2371
54.73685	1.24191
54.93731	1.23816
55.1368	1.24246
55.33824	1.24264
55.53871	1.24305
55.73917	1.23797
55.93864	1.44864
56.1401	1.82567
56.33957	2.08526
56.54103	2.3752
56.7415	2.71317
56.94196	2.9104
57.14143	2.96715
57.34289	2.98313
57.54338	3.01473
57.74282	3.02291
57.94329	3.04719
58.14375	3.0644
58.34421	3.07713
58.54468	3.08426
58.74616	3.08311
58.94568	3.09002
59.14707	3.08931
59.34657	3.09006
59.548	3.09619
59.74747	3.10017
59.94893	3.10094
60.14939	3.10404
60.34986	3.10914
60.55033	3.10713
60.75079	3.10773
60.95131	3.10996
61.15072	3.11117
61.35125	3.10996
61.55068	3.11448
61.75212	3.11324
61.95158	3.11694
62.15304	3.11182
62.35255	3.11429
62.553	3.1159

62.75244	3.1183
62.95291	3.11773
63.15343	3.11675
63.35484	3.12098
63.5563	3.12251
63.75676	3.11772
63.95723	3.11909
64.15669	3.11762
64.35816	3.12155
64.55762	3.12285
64.75815	3.12446
64.95761	3.12718
65.15902	3.12748
65.35951	3.12853
65.55995	3.12454
65.76141	3.12659
65.96187	3.12963
66.16234	3.12826
66.3628	3.12878
66.56327	3.1299
66.76374	3.13331
66.9642	3.13076
67.16466	3.08782
67.36513	3.01649
67.56459	2.97428
67.76606	2.96945
67.96652	2.97168
68.16798	2.97028
68.36745	2.97068
68.56793	2.97295
68.76938	2.96646
68.96887	2.96701
69.17031	2.96717
69.36984	2.96968
69.57024	2.96639
69.76971	2.96865
69.97117	2.9647
70.17163	2.96864
70.3721	2.96927
70.57256	2.97057
70.77403	2.97007
70.97349	2.96774

71.17396	2.96848
71.37449	2.96603
71.57601	2.96488
71.77638	2.96582
71.97681	2.9694
72.17728	2.96706
72.37675	2.96564
72.57821	2.96503
72.77767	2.96237
72.97916	2.96414
73.1796	2.96327
73.38007	2.96201
73.57959	2.96
73.78003	2.96
73.98046	2.96213
74.18093	2.96291
74.38239	2.95998
74.58285	2.95941
74.78432	2.95791
74.98378	2.95741
75.18525	2.9583
75.38571	2.95661
75.58617	2.95777
75.78564	2.96103
75.98613	2.958
76.18663	2.9589
76.38704	2.96009
76.58651	2.96043
76.787	2.96292
76.98744	2.96353
77.1879	2.96039
77.38839	2.96421
77.58883	2.96107
77.78929	2.81325
77.98876	2.6398
78.19022	2.49255
78.39069	2.3727
78.59221	2.32512
78.79261	2.32513
78.99407	2.31979
79.19454	2.32009
79.39503	2.31308

79.59453	2.31676
79.79593	2.31806
79.99746	2.31622
80.19792	2.31632
80.39833	2.31475
80.59782	2.31331
80.79826	2.31366
80.99875	2.31301
81.20018	2.31498
81.40065	2.31274
81.60217	2.31331
81.80158	2.31104
82.00204	2.31374
82.20251	2.31186
82.40397	2.31004
82.60543	2.30982
82.80495	2.30584
83.00639	2.31081
83.20586	2.30983
83.40629	2.30949
83.60579	2.30532
83.80725	2.30957
84.00775	2.30676
84.20915	2.30703
84.40862	2.30503
84.61011	2.30495
84.80955	2.30707
85.01001	2.30392
85.21048	2.30316
85.41094	2.30263
85.6124	2.30436
85.8119	2.30038
86.01333	2.30425
86.2128	2.30243
86.41327	2.30221
86.61373	2.30065
86.81519	2.30478
87.01571	2.30188
87.21712	2.3002
87.41659	2.2987
87.61805	2.2994
87.81751	2.30359

88.01798	2.29888
88.21844	2.3
88.41794	2.30108
88.61843	2.29923
88.81784	2.30041
89.01837	2.30011
89.2178	2.297
89.41924	2.298
89.6197	2.29542
89.82117	2.29881
90.02063	2.29721
90.22209	2.29755
90.4216	2.29819
90.62302	2.29654
90.82349	2.29925
91.02395	2.29425
91.22541	2.297
91.42488	2.2982
91.62537	2.29705
91.82481	2.29535
92.02528	2.29552
92.22477	2.29543
92.42624	2.29523
92.62668	2.29679
92.82813	2.29387
93.02866	2.29641
93.22909	2.29378
93.42853	2.29536
93.62903	2.29649
93.82946	2.29366
94.02896	2.29567
94.23039	2.29543
94.42986	2.296
94.63032	2.29208
94.82982	2.29448
95.03125	2.29729
95.2308	2.29317
95.43125	2.29769
95.63068	2.29638
95.83214	2.29393
96.03264	2.29383
96.23404	2.29767

96.4355	2.30206
96.63599	2.29486
96.83649	2.29652
97.03595	2.29502
97.2364	2.29619
97.43583	2.29707
97.63735	2.29631
97.83776	2.29786
98.03825	2.28062
98.23769	1.84581
98.43815	1.56498
98.63862	1.44843
98.83908	1.18703
99.03855	0.82945
99.23902	0.52649
99.43948	0.43874
99.63898	0.35751
99.84041	0.28648
100.0399	0.21841
100.2413	0.19633
100.4418	0.19853
100.6433	0.19089
100.8438	0.19206
101.0443	0.18993
101.2437	0.18515
101.4441	0.18625
101.6436	0.18908
101.8452	0.18694
102.0465	0.18338
102.247	0.18357
102.4485	0.18292
102.6489	0.18059
102.8504	0.18356
103.0509	0.18724
103.2513	0.18435
103.4508	0.18798
103.6522	0.18563
103.8527	0.18475
104.0532	0.18177
104.2536	0.18063
104.4541	0.18701
104.6536	0.18261

104.854	0.18376
105.0555	0.18466
105.256	0.18209
105.4564	0.18351
105.6559	0.18312
105.8573	0.18188
106.0568	0.18153
106.2573	0.18085
106.4568	0.17651
106.6572	0.1809
106.8577	0.18399
107.0591	0.1803
107.2597	0.17586
107.4601	0.1794
107.6605	0.17855
107.86	0.17982
108.0605	0.17789
108.2599	0.18236
108.4604	0.18271
108.6609	0.17796
108.8623	0.18011
109.0618	0.17446
109.2623	0.18021
109.4627	0.18054
109.6642	0.17768
109.8637	0.18046
110.0641	0.17854
110.2636	0.1745
110.4642	0.17791
110.6656	0.17715
110.866	0.18099
111.0664	0.17569
111.2669	0.18174
111.4674	0.17849
111.6668	0.18004
111.8673	0.17866
112.0668	0.17683
112.2682	0.17576
112.4677	0.17419
112.6682	0.17623
112.8676	0.17845
113.0681	0.17747

113.2696	0.17558
113.47	0.17873
113.6715	0.17371
113.8709	0.17289
114.0724	0.17354
114.2729	0.17763
114.4733	0.1744
114.6728	0.17352
114.8743	0.17319
115.0737	0.18069
115.2742	0.17514
115.4737	0.17427
115.6741	0.17634
115.8736	0.17335
116.0751	0.17554
116.2765	0.17562
116.477	0.17714
116.6775	0.17654
116.8769	0.17954
117.0784	0.17474
117.2779	0.17576
117.4793	0.17873
117.6798	0.17582
117.8832	0.17798
118.0837	0.17577
118.2842	0.17643
118.4836	0.17769
118.6851	0.18012
118.8856	0.17409
119.085	0.17821
119.2855	0.17848
119.486	0.17661
119.6864	0.17627
119.8859	0.17109
120.0873	0.17625
120.2868	0.17763
120.4883	0.1825
120.6888	0.21425
120.8892	0.36828
121.0897	0.47783
121.2902	0.58354
121.4906	0.70704

121.6901	0.75442
121.8915	0.76996
122.091	0.78817
122.2925	0.81516
122.4919	0.81564
122.6934	0.81825
122.8939	0.81872
123.0954	0.82054
123.2958	0.8198
123.4973	0.81817
123.6968	0.82291
123.8982	0.82359
124.0986	0.82344
124.3001	0.82156
124.5016	0.82246
124.7011	0.82286
124.9025	0.82042
125.103	0.82363
125.3044	0.82572
125.5039	0.82338
125.7044	0.82156
125.9048	0.82347
126.1063	0.823
126.3058	0.82231
126.5063	0.82259
126.7067	0.82054
126.9082	0.82104
127.1086	0.81987
127.3091	0.82175
127.5105	0.82036
127.71	0.81927
127.9105	0.82008
128.1109	0.8208
128.3124	0.82124
128.5119	0.8203
128.7123	0.82059
128.9128	0.82232
129.1133	0.82234
129.3127	0.82475
129.5142	0.82434
129.7147	0.82439
129.9151	0.8273

130.1156	0.827
130.3161	0.82633
130.5175	0.83044
130.717	0.82902
130.9184	0.82881
131.1189	0.82805
131.3194	0.82843
131.5198	0.82815
131.7213	0.82821
131.9208	0.81727
132.1212	0.79537
132.3207	0.74498
132.5212	0.71595
132.7216	0.674
132.9221	0.638
133.1226	0.58871
133.322	0.55039
133.5235	0.53821
133.7239	0.53162
133.9254	0.52442
134.1249	0.51186
134.3253	0.50053
134.5248	0.50012
134.7263	0.50111
134.9267	0.50391
135.1272	0.5016
135.3267	0.50195
135.5272	0.50128
135.7276	0.5005
135.9281	0.49832
136.1295	0.5036
136.329	0.50231
136.5295	0.49993
136.7289	0.50013
136.9294	0.49959
137.1289	0.49997
137.3294	0.5018
137.5288	0.50197
137.7302	0.50142
137.9307	0.49909
138.1312	0.50072
138.3317	0.50391

138.5311	0.50119
138.7326	0.50375
138.932	0.50044
139.1325	0.50313
139.332	0.50371
139.5334	0.50296
139.7339	0.50439
139.9354	0.50346
140.1358	0.50298
140.3363	0.50565
140.5367	0.50618
140.7392	0.50397
140.9397	0.50335
141.1391	0.50505
141.3406	0.50628
141.5401	0.50274
141.7405	0.50394
141.94	0.50996
142.1415	0.50791
142.3409	0.50675
142.5414	0.50988
142.7409	0.50907
142.9413	0.50571
143.1408	0.50443
143.3423	0.51124
143.5428	0.50568
143.7442	0.50595
143.9447	0.51917
144.1461	0.77713
144.3466	1.22038
144.547	1.45265
144.7475	1.64778
144.948	2.00104
145.1494	2.3817
145.3489	2.56126
145.5504	2.57721
145.7508	2.58976
145.9523	2.59389
146.1518	2.60024
146.3523	2.60281
146.5527	2.60502
146.7521	2.6037

146.9526	2.60582
147.1521	2.61007
147.3535	2.60839
147.554	2.61015
147.7545	2.61036
147.9549	2.61528
148.1554	2.61214
148.3549	2.61546
148.5553	2.61593
148.7548	2.61314
148.9563	2.61737
149.1557	2.61301
149.3573	2.62039
149.5577	2.61741
149.7582	2.61527
149.9596	2.61846
150.1601	2.6195
150.3615	2.61898
150.562	2.62335
150.7624	2.61864
150.9619	2.61891
151.1624	2.62226
151.3618	2.6205
151.5623	2.62642
151.7618	2.62285
151.9632	2.62817
152.1638	2.6288
152.3652	2.62685
152.5657	2.62849
152.7661	2.62418
152.9666	2.62813
153.167	2.62615
153.3675	2.63065
153.5669	2.62673
153.7684	2.63139
153.9689	2.633
154.1693	2.63686
154.3698	2.63167
154.5713	2.63363
154.7707	2.63512
154.9722	2.63336
155.1727	2.63311

155.3732	2.63679
155.5736	2.63438
155.7741	2.62952
155.9756	2.61949
156.175	2.43597
156.3764	2.2454
156.5759	2.08228
156.7764	1.94374
156.9758	1.88677
157.1773	1.87603
157.3768	1.88155
157.5772	1.87231
157.7777	1.87377
157.9772	1.87079
158.1776	1.87038
158.3772	1.87064
158.5786	1.86943
158.778	1.87044
158.9795	1.86946
159.179	1.86978
159.3804	1.86362
159.5799	1.86747
159.7804	1.86548
159.9798	1.86537
160.1803	1.86061
160.3798	1.86761
160.5812	1.8659
160.7827	1.86327
160.9831	1.86373
161.1846	1.85996
161.3841	1.86343
161.5845	1.86249
161.784	1.86296
161.9855	1.86131
162.1849	1.86314
162.3864	1.86135
162.5869	1.86085
162.7873	1.86004
162.9878	1.8594
163.1883	1.86314
163.3887	1.8613
163.5882	1.86313

163.7887	1.85849
163.9881	1.85757
164.1886	1.85774
164.3881	1.85823
164.5895	1.85789
164.79	1.85808
164.9904	1.85802
165.1899	1.85765
165.3914	1.8565
165.5918	1.85969
165.7933	1.85689
165.9938	1.85944
166.1933	1.85934
166.3937	1.85792
166.5942	1.85715
166.7946	1.85761
166.9941	1.85621
167.1946	1.85674
167.394	1.85949
167.5945	1.85735
167.795	1.85976
167.9964	1.85916
168.1959	1.85799
168.3973	1.85965
168.5969	1.85887
168.7983	1.85588
168.9987	1.85784
169.1982	1.85684
169.3987	1.85766
169.5992	1.85665
169.7996	1.857
170.0011	1.85476
170.2015	1.85776
170.402	1.85651
170.6025	1.85935
170.8029	1.85503
171.0044	1.85794
171.2039	1.85891
171.4043	1.86055
171.6058	1.85572
171.8052	1.86163
172.0067	1.85717

172.2072	1.8593
172.4086	1.8596
172.6091	1.85745
172.8106	1.85891
173.01	1.86054
173.2105	1.86424
173.41	1.8628
173.6115	1.85916
173.8119	1.86154
174.0134	1.86302
174.2138	1.86601
174.4143	1.86446
174.6147	1.86623
174.8152	1.86744
175.0157	1.86461
175.2162	1.86183
175.4166	1.85987
175.6171	1.69465
175.8185	1.43006
176.018	1.27369
176.2195	1.15883
176.4199	0.97737
176.6214	0.68097
176.8209	0.53548
177.0223	0.43366
177.2228	0.37409
177.4232	0.36325
177.6237	0.36079
177.8232	0.35971
178.0246	0.36085
178.2241	0.35502
178.4256	0.32248
178.625	0.30864
178.8255	0.31443
179.026	0.31093
179.2274	0.30868
179.428	0.30778
179.6294	0.30418
179.8298	0.31026
180.0293	0.30871
180.2298	0.30502
180.4303	0.30385

180.6317	0.30614
180.8311	0.30762
181.0316	0.30575
181.2311	0.30692
181.4326	0.30708
181.633	0.30505
181.8335	0.30329
182.0329	0.30869
182.2334	0.3064
182.4339	0.3068
182.6343	0.30475
182.8348	0.30416
183.0353	0.30544
183.2358	0.30368
183.4362	0.30015
183.6367	0.30495
183.8372	0.30153
184.0376	0.30095
184.2381	0.30043
184.4395	0.2996
184.639	0.29814
184.8394	0.29862
185.0389	0.30168
185.2394	0.29955
185.4398	0.30021
185.6413	0.29909
185.8418	0.30002
186.0423	0.2988
186.2437	0.29679
186.4432	0.29808
186.6437	0.30064
186.8431	0.29854
187.0446	0.2961
187.244	0.2992
187.4445	0.30054
187.644	0.2974
187.8454	0.29937
188.0459	0.29905
188.2464	0.29762
188.4468	0.29618
188.6473	0.29978
188.8487	0.29696

189.0492	0.29704
189.2507	0.29624
189.4511	0.29461
189.6526	0.29595
189.8531	0.29765
190.0535	0.29408
190.253	0.29616
190.4544	0.29422
190.6549	0.29677
190.8564	0.29489
191.0568	0.2975
191.2564	0.29712
191.4578	0.29717
191.6582	0.29761
191.8597	0.29572
192.0592	0.29617
192.2597	0.29924
192.4591	0.29689
192.6606	0.29617
192.86	0.29728
193.0605	0.30136
193.26	0.29785
193.4614	0.29834
193.6619	0.29741
193.8623	0.30201
194.0638	0.2981
194.2633	0.29952
194.4647	0.30024
194.6642	0.30341
194.8657	0.30121
195.0651	0.30006
195.2666	0.30231
195.4671	0.30388
195.6675	0.30388
195.868	0.30129
196.0685	0.30069
196.2689	0.30442
196.4704	0.30505
196.6708	0.30632
196.8703	0.31066
197.0708	0.37202
197.2712	0.50766

197.4717	0.59226
197.6722	0.69115
197.8736	0.77243
198.0741	0.86568
198.2756	0.90403
198.475	0.90572
198.6765	0.90448
198.876	0.90567
199.0765	0.90563
199.2759	0.90517
199.4774	0.90997
199.6778	0.90553
199.8773	0.91024
200.0788	0.90584
200.2792	0.9064
200.4797	0.90995
200.6791	0.90684
200.8806	0.90794
201.0811	0.90294
201.2825	0.90513
201.483	0.90721
201.6835	0.90966
201.8829	0.90675
202.0844	0.90864
202.2859	0.90934
202.4863	0.90674
202.6878	0.90628
202.8873	0.90469
203.0887	0.90529
203.2892	0.90345
203.4897	0.90628
203.6891	0.90449
203.8906	0.9067
204.091	0.90781
204.2925	0.90569
204.493	0.90507
204.6935	0.90637
204.8949	0.9027
205.0944	0.90309
205.2948	0.90777
205.4953	0.90632
205.6958	0.90767

205.8952	0.90502
206.0957	0.90995
206.2952	0.90752
206.4966	0.90495
206.6971	0.90716
206.8985	0.95371
207.098	1.12316
207.2985	1.17829
207.4989	1.18168
207.6994	1.18973
207.9009	1.18602
208.1004	1.1854
208.3018	1.18732
208.5033	1.18699
208.7047	1.18793
208.9042	1.19218
209.1056	1.19201
209.3051	1.19003
209.5066	1.18973
209.707	1.18921
209.9075	1.18855
210.107	1.18931
210.3074	1.19014
210.5079	1.19277
210.7074	1.19148
210.9079	1.19195
211.1074	1.19143
211.3088	1.18904
211.5083	1.18647
211.7087	1.18759
211.9082	1.19049
212.1086	1.18899
212.3081	1.192
212.5096	1.18917
212.7101	1.18893
212.9115	1.19092
213.1119	1.18845
213.3134	1.19377
213.5129	1.19365
213.7143	1.19486
213.9148	1.19382
214.1143	1.1922

214.3149	1.1962
214.5143	1.19621
214.7157	1.19395
214.9162	1.19651
215.1176	1.19336
215.3171	1.191
215.5185	1.19178
215.718	1.19472
215.9185	1.19288
216.1199	1.19266
216.3194	1.1939
216.5199	1.19271
216.7193	1.19688
216.9208	1.19904
217.1212	1.19215
217.3227	1.19239
217.5222	1.19647
217.7237	1.19495
217.9231	1.19581
218.1236	1.19397
218.323	1.19619
218.5245	1.19743
218.724	1.19803
218.9254	1.33049
219.1269	1.47435
219.3274	1.80663
219.5278	1.85833
219.7273	1.86491
219.9288	1.86753
220.1283	1.86883
220.3287	1.86952
220.5291	1.87023
220.7296	1.87107
220.9291	1.8732
221.1305	1.87478
221.331	1.87542
221.5315	1.87449
221.7319	1.8763
221.9324	1.87702
222.1329	1.87225
222.3323	1.87715
222.5328	1.88

222.7323	1.87892
222.9327	1.87806
223.1322	1.879
223.3327	1.88048
223.5331	1.88154
223.7337	1.88095
223.9331	1.88442
224.1336	1.89079
224.333	1.88815
224.5334	1.88892
224.7349	1.8895
224.9354	1.88525
225.1358	1.88695
225.3363	1.88792
225.5378	1.88742
225.7372	1.88964
225.9387	1.88776
226.1392	1.88396
226.3406	1.88493
226.5411	1.88543
226.7416	1.88736
226.942	1.88534
227.1435	1.8851
227.3439	1.88019
227.5444	1.88225
227.7459	1.88644
227.9453	1.88596
228.1468	1.88582
228.3463	1.88728
228.5467	1.88543
228.7472	1.88847
228.9477	1.88656
229.1481	1.88392
229.3496	1.88651
229.5491	1.88738
229.7496	1.88526
229.95	1.88812
230.1495	1.88467
230.3509	1.88588
230.5514	1.8869
230.7528	1.8891
230.9533	1.88849

231.1548	1.92667
231.3552	2.06328
231.5567	2.13853
231.7572	2.19015
231.9587	2.22131
232.1581	2.28919
232.3596	2.30089
232.559	2.305
232.7595	2.30935
232.961	2.31095
233.1614	2.31694
233.3629	2.31582
233.5633	2.31968
233.7648	2.3211
233.9653	2.3201
234.1657	2.32368
234.3652	2.32683
234.5667	2.32032
234.7661	2.32226
234.9666	2.32186
235.1661	2.32102
235.3675	2.32123
235.568	2.32495
235.7684	2.32312
235.969	2.32085
236.1684	2.32376
236.3688	2.32549
236.5683	2.32183
236.7688	2.32183
236.9683	2.32964
237.1697	2.32605
237.3702	2.32377
237.5706	2.32136
237.7701	2.32222
237.9716	2.32376
238.172	2.32523
238.3725	2.32282
238.573	2.32742
238.7734	2.32435
238.9739	2.32074
239.1744	2.32341
239.3748	2.32874

239.5743	2.32635
239.7748	2.32771
239.9742	2.3296
240.1757	2.33171
240.3751	2.33189
240.5766	2.33436
240.7761	2.33345
240.9775	2.33154
241.178	2.334
241.3785	2.33313
241.58	2.33304
241.7804	2.33316
241.9809	2.33533
242.1804	2.3332
242.3808	2.33379
242.5803	2.33019
242.7817	2.33315
242.9812	2.32969
243.1827	2.33411
243.3831	2.33463
243.5836	2.33453
243.783	2.3349
243.9845	2.33611
244.185	2.33502
244.3854	2.33665
244.5859	2.3293
244.7864	2.33267
244.9868	2.33423
245.1864	2.33439
245.3878	2.33811
245.5883	2.33584
245.7897	2.3377
245.9892	2.33713
246.1906	2.3359
246.3911	2.33503
246.5916	2.33432
246.793	2.33263
246.9925	2.33603
247.1939	2.33409
247.3935	2.33189
247.5949	2.33477
247.7944	2.33129

247.9948	2.33032
248.1953	2.33365
248.3958	2.32853
248.5952	2.32959
248.7957	2.32999
248.9951	2.33512
249.1966	2.32922
249.3971	2.33286
249.5966	2.33222
249.797	2.33326
249.9965	2.32751
250.1979	2.33079
250.3974	2.32899
250.5988	2.33293
250.7983	2.33108
250.9988	2.33114
251.1982	2.33363
251.3997	2.33257
251.5992	2.33529
251.7996	2.33508
252.0001	2.33554
252.2016	2.3068
252.403	1.85094
252.6025	1.60749
252.803	1.208
253.0024	0.86417
253.2039	0.6766
253.4044	0.39713
253.6048	0.12336
253.8053	0.02977
254.0058	0.00995
254.2062	0.00692
254.4077	0.00975
254.6081	0.01064
254.8086	0.00854
255.0091	0.00819
255.2096	0.00551
255.41	0.00631
255.6095	0.00964
255.8109	0.00967
256.0104	0.00883
256.2109	0.00944

256.4113	0.01025
256.6118	0.00735
256.8123	0.0086
257.0127	0.00973
257.2122	0.00744
257.4136	0.00625
257.6141	0.00884
257.8156	0.0042
258.017	0.00833
258.2175	0.00795
258.419	0.005
258.6184	0.00566
258.8199	0.00584
259.0194	0.00974
259.2208	0.00918
259.4203	0.00642
259.6218	0.00778
259.8212	0.00773
260.0217	0.0058
260.2222	0.0065
260.4236	0.00765
260.6251	0.00729
260.8255	0.00719
261.026	0.00554
261.2255	0.01008
261.426	0.00833
261.6254	0.00779
261.8259	0.00563
262.0253	0.00817
262.2268	0.01021
262.4263	0.00949
262.6267	0.0107
262.8272	0.01385
263.0277	0.01377
263.2281	0.01014
263.4286	0.01486
263.6301	0.01102
263.8295	0.01305
264.03	0.01171

264.2305	0.01149
264.4309	0.01287
264.6304	0.01552
264.8308	0.0149
265.0303	0.00943
265.2308	0.01229
265.4302	0.01531
265.6307	0.01418
265.8302	0.01525
266.0306	0.01134
266.2311	0.01259
266.4306	0.01357
266.632	0.00823
266.8325	0.0098
267.033	0.01223
267.2344	0.0097
267.4359	0.01027
267.6364	0.00982
267.8378	0.01092
268.0373	0.01181
268.2387	0.01387
268.4382	0.009
268.6397	0.00911
268.8401	0.0128
269.0406	0.01042
269.2411	0.01401
269.4415	0.01067
269.642	0.00868
269.8415	0.00862
270.0419	0.00783
270.2425	0.00933
270.4439	0.01083
270.6443	0.00612
270.8448	0.0064
271.0443	0.0075
271.2447	0.00786
271.4442	0.01034
271.6456	0.01081
271.8471	0.00678

272.0476	0.01167
272.249	0.00914
272.4485	0.01147
272.649	0.01053
272.8484	0.01209
273.0499	0.0127
273.2504	0.01166
273.4508	0.01343
273.6503	0.01234
273.8518	0.0151
274.0522	0.01119
274.2537	0.01321
274.4541	0.01243
274.6536	0.01245
274.8551	0.01226
275.0555	0.01277
275.257	0.01603
275.4565	0.01237
275.6569	0.01475
275.8564	0.01295
276.0569	0.00922
276.2564	0.01257
276.4568	0.01238
276.6573	0.01175
276.8577	0.01142
277.0572	0.01412
277.2587	0.01078
277.4601	0.01679
277.6596	0.01066
277.861	0.01312
278.0615	0.01044
278.262	0.01177
278.4615	0.01204
278.6619	0.01409
278.8614	0.01235
279.0628	0.0133
279.2633	0.01215
279.4638	0.00964
279.6632	0.00943

279.8647	0.0087
280.0652	0.0142
280.2656	0.01355
280.4661	0.0129
280.6656	0.01112
280.867	0.01137
281.0675	0.01344
281.268	0.00835
281.4674	0.01202
281.6679	0.01325
281.8683	0.01101
282.0688	0.0102
282.2693	0.00918
282.4707	0.00727
282.6702	0.0091
282.8717	0.00961
283.0722	0.00738
283.2716	0.00589
283.4731	0.01048
283.6735	0.00663
283.875	0.00622
284.0755	0.00832
284.277	0.00494
284.4774	0.00776
284.6788	0.00952
284.8793	0.00843
285.0798	0.00998
285.2802	0.00867
285.4817	0.00914
285.6822	0.0051
285.8826	0.00546
286.0832	0.00662
286.2826	0.00724
286.484	0.00719
286.6845	0.00735
286.886	0.00615
287.0864	0.00842
287.2879	0.01335
287.4873	0.00636

287.6878	0.00719
287.8883	0.00719
288.0897	0.00711
288.2892	0.00681
288.4907	0.00751
288.6912	0.00901
288.8906	0.00729
289.0921	0.00607
289.2925	0.00167
289.494	0.01087
289.6935	0.00764
289.8949	0.01173
290.0954	0.00624
290.2968	0.01013
290.4973	0.01119
290.6978	0.00942
290.8972	0.01466
291.0987	0.01526
291.3002	0.01631
291.5006	0.01716
291.7021	0.01186
291.9036	0.01333
292.105	0.00514
292.3045	0.00743
292.5049	0.00576
292.7054	9.55E-04
292.9069	0.0039
293.1073	0.00353
293.3088	0.00199
293.5093	0.00195
293.7107	0.00422
293.9112	5.15E-04
294.1117	2.13E-04
294.3131	0
